# Subacute effects of ketamine on neural correlates of reward processing

**DOI:** 10.1038/s41398-026-04485-6

**Published:** 2026-09-25

**Authors:** Elisa Briem, Peter Stöhrmann, Gregor Dörl, Christian Milz, Gabriel Schlosser, Manfred Klöbl, Murray Reed, Matthieu Atger, Clemens Schmidt, Samantha Graf, Maximilian Kathofer, Godber Mathis Godbersen, Julia Sophia Crone, Rupert Lanzenberger, Marie Spies

**Affiliations:** 1 Department of Psychiatry and Psychotherapy, Medical University of Vienna, Vienna, Austria; 2 Comprehensive Center for Clinical Neurosciences and Mental Health (C3NMH), Medical University of Vienna, Vienna, Austria; 3 Vienna Cognitive Science Hub, University of Vienna, Vienna, Austria; 4 Faculty of Psychology, University of Vienna, Vienna, Austria; 5 Department of Psychology & Centre for Cognitive Neuroscience, University of Salzburg, Salzburg, Austria

**Keywords:** Neuroscience, Biomarkers, Depression

## Abstract

Ketamine induces a sequence of temporally distinct effects on brain function, yet its impact on reward processing beyond the acute post-administration phase remains unclear. In this single-blind, placebo-controlled, crossover study, 28 healthy participants received 0.5 mg/kg racemic ketamine or placebo via a 40 min intravenous infusion. Functional magnetic resonance imaging was acquired approximately 5 h post-infusion during the Monetary Incentive Delay (MID) task, targeting the subacute phase after resolution of acute dissociative effects. Plasma concentrations of ketamine and norketamine were obtained for pharmacokinetic modeling and area under the curve estimation. At 5 h post-administration, ketamine did not significantly modulate brain activation during the MID task in either ROI-based or whole-brain analyses, despite measurable subjective drug effects. Pharmacokinetic modeling yielded ketamine and norketamine concentration-time profiles consistent with expected pharmacokinetic patterns, however, neither individual drug exposure nor psychometric measures showed robust associations with MID task-related neural activation. These findings suggest an absence of detectable task-evoked effects under the present methodological conditions. When considered alongside prior work, they are consistent with the possibility that ketamine’s effects on reward circuitry are time-sensitive and may be more prominent during the acute post-administration phase or in clinical populations with pre-existing reward dysfunction.

## Introduction

Ketamine’s rapid-acting antidepressant effects have led to a major shift in psychiatric research in recent years. Unlike traditional monoaminergic antidepressants, ketamine elicits rapid antidepressant effects within 2–4 h and sustained effects lasting up to 7 days following a single subanesthetic dose [1–3]. Ketamine exhibits a distinct neuropsychopharmacological profile characterized by acute, subacute, and sustained phases. Acute dissociative and psychotomimetic effects emerge during infusion, peak within the first hour and largely resolve within approximately 2 h post-infusion [1–3]. These transient subjective effects are temporally separated from the antidepressant response, which continues to evolve beyond the acute phase of dissociation, with maximal clinical effects commonly observed around 24 h post-infusion [2, 3]. Neuroimaging findings suggest that ketamine induces circuit-level alterations in healthy individuals, including increased prefrontal-hippocampal connectivity 20 min after and decreased fronto-striatal connectivity 2 d after ketamine infusion [4, 5]. Task-based fMRI studies in healthy volunteers have predominantly focused on emotional processing and working memory performance [6–8], while functional consequences of ketamine on motivational processes remain poorly understood.

Dysfunction in reward anticipation and outcome evaluation is consistently implicated in multiple psychiatric disorders, including major depressive disorder (MDD), bipolar disorder, schizophrenia, and substance use disorders, highlighting altered reward processing as a transdiagnostic neurocognitive feature [9–12]. Despite the central role of reward processing across psychiatric disorders, very few neuroimaging studies have examined ketamine’s effects on reward processing directly. In healthy individuals, evidence is essentially limited to a single placebo-controlled fMRI study demonstrating attenuated ventral striatal responses during reward anticipation approximately 40 min after ketamine infusion [13]. In patients with remitted depression, ketamine has been shown to produce detectable changes in reward-related brain areas 2 h post-infusion, including increased activations in the nucleus accumbens (NAc) and the putamen during the feedback phase of low-win trials compared with neutral trials, without coincident changes in depressive symptoms [14].

Reward processing relies on coordinated activity across the striatum, insula, amygdala, thalamus, and dopaminergic mesocorticolimbic regions [15], enabling adaptive decision-making and goal-directed behavior [16]. The Monetary Incentive Delay (MID) task is a well-established paradigm for investigating reward processing, predominantly combined with functional magnetic resonance imaging (fMRI) [15, 17–19], and has been previously applied by our group [20, 21]. The MID task comprises two phases: anticipation, when a cue signals potential reward or loss, and outcome, when the reward or loss is obtained or omitted. Anticipation activates regions such as the striatum, insula, thalamus, and amygdala [15, 17, 18]. Similar regions are also active during the outcome phase, suggesting overlapping neural circuits in different stages of reward processing [15, 17, 18]. The insula, amygdala and thalamus are commonly implicated in salience processing and the detection of motivationally relevant stimuli [15, 22, 23], whereas the striatum, particularly the NAc, has been linked to reward magnitude [24, 25], reward sensitivity [25–27] and the subjective experience of motivation [24].

While acute modulatory effects of ketamine on reward processing during the MID task have been observed during the acute phase, including 40 min post-infusion in healthy subjects [13] and 2 h post-infusion in patients with remitted depression [14], subacute effects remain underexplored. Beyond 2 h post-infusion, ketamine plasma levels have substantially declined [28, 29] and dissociative symptoms have largely resolved [1–3], while early molecular and synaptic plasticity processes are thought to unfold [30–33]. Whether reward-related brain networks normalize, persist, or reorganize during this intermediate period remains unknown.

From a behavioral perspective, the subacute post-infusion phase is characterized by resolution of acute dissociative and sedative effects, with relatively stable alertness and task performance. Assessing reward circuitry during this subacute time window (~2–6 h post-infusion) may provide insight into the timeline of early neuromodulatory changes that could be relevant for optimizing the timing of behavioral or therapeutic interventions following ketamine administration.

In the present study, we used task-based fMRI during the MID task to investigate the subacute effects of ketamine (~5 h post-infusion) on reward circuitry in healthy individuals. This approach enables the assessment of ketamine-related modulation of reward processing after resolution of acute subjective effects and independent of clinical symptom change. Based on evidence that ketamine’s antidepressant effects emerge after resolution of acute psychotomimetic effects [1–3], and neuroimaging findings in clinical populations reporting enhanced striatal and reward-related responses at later time points [14, 34, 35], we hypothesized that ketamine would increase reward-related neural activation during the subacute post-infusion window (~5 h). Given prior reports of attenuated reward-related responses during the acute phase in healthy individuals [13], we suggest that ketamine’s effects on reward circuitry may be time-dependent.

## Subjects and methods

### Participants

Participants were required to be 18–55 years old, right-handed, in good general and psychiatric health (based on medical history, physical examination, and structured DSM-5 interview), and able to provide informed consent. Current or past psychiatric diagnosis, relevant medical illness, pregnancy or breastfeeding, MRI contraindications, substance abuse, or prior ketamine use were defined as exclusion criteria. Urine drug screening and pregnancy testing (in females) were performed at each visit. All participants provided written informed consent prior to enrollment.

### Study design

This study was approved by the ethics committee of the Medical University of Vienna, Austria (EK 1572/2021) and performed in accordance with the Declaration of Helsinki.

**Fig. 1:**
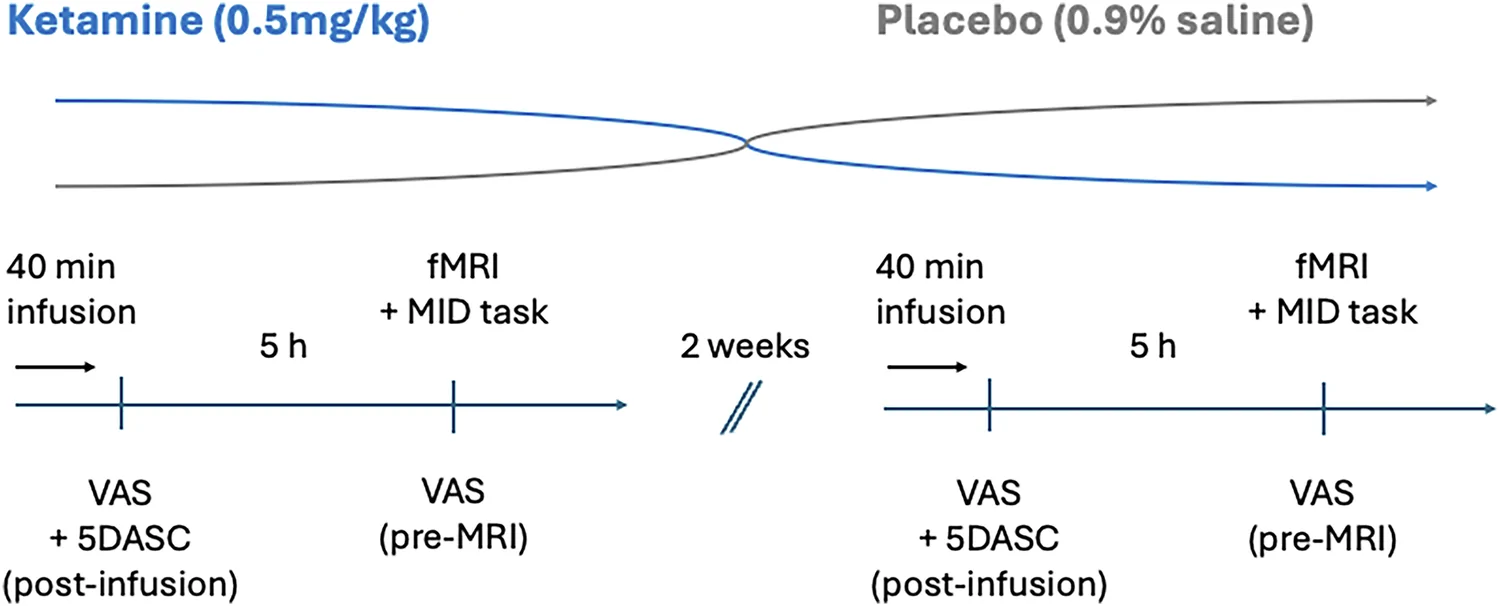
Single-blind, placebo-controlled, crossover study design. Participants received either ketamine (0.5 mg/kg, 40 min i.v. infusion) or placebo (0.9% saline) in two separate sessions, spaced approximately 2 weeks apart, with treatment order randomized across participants. Following each infusion, subjective drug effects were assessed using a visual analog scale (VAS) to assess common ketamine-related adverse effects and a 5D-ASC questionnaire immediately post-infusion. Approximately 5 h later, participants underwent fMRI scanning while performing the Monetary Incentive Delay (MID) task, with VAS ratings collected immediately before scanning.

The study followed a single-blind, placebo-controlled, crossover design consisting of four sessions: a screening visit, two experimental sessions (ketamine and placebo, respectively, in a randomized order, each including MRI scanning and spaced at least two weeks apart; Fig. 1), and an end-of-study visit. At MRI sessions, subjects received either placebo (0.9% saline solution) or 0.5 mg/kg body weight ketamine diluted in 0.9% saline solution intravenously over 40 min. The study medication (50 mg/mL ampoules; Hameln Pharma Plus GmbH) was prepared by a medical doctor before each session. Participants were blinded to treatment condition; investigators involved in data acquisition were not blinded. Participants were observed in a clinical setting for at least 90 min post-infusion until full recovery from ketamine-induced sedative and dissociative effects and remained at the study site under supervision of medically trained staff until MRI scanning. They were only allowed to engage in low-stimulation activities (e.g., resting, conversation) and no substance intake (except a light meal and water) was permitted during this interval.

### Psychometric measures

Subjective drug effects as experienced during ketamine application were assessed using the 5-dimensional altered states of consciousness rating scale (5D-ASC), a validated questionnaire designed to assess altered states of consciousness induced by psychoactive substances such as ketamine [36, 37]. Participants completed the questionnaire on a tablet in a quiet room under supervision immediately post-infusion. The 94 items of the 5D-ASC were scored on a scale ranging from 0 (no experience) to 100 (strongest possible experience). A total 5D-ASC score was computed by summing the scores of all 94 items, yielding a maximum possible score of 9400, with higher scores indicating more pronounced subjective drug effects. In addition to the 5D-ASC, participants completed two brief visual analog scale (VAS) assessments to evaluate the duration and intensity of common ketamine-associated adverse effects, including dizziness, blurred vision, headache, nausea, dry mouth, poor coordination, and restlessness [3]. Each of the seven symptoms was rated individually from 0 (“no symptom”) to 100 (“most severe”), and a total VAS score was obtained by summing the ratings across all symptoms. VAS assessments were conducted shortly after infusion stop as well as immediately before MRI scanning. Additionally, participants were asked to guess whether they had received ketamine or placebo and to rate their confidence in this judgment on a scale from 0 (“not sure”) to 100 (“completely sure”).

### fMRI scanning

Task-based fMRI scanning was performed approximately 5 h (mean: 5 h 9 min ± 6 min) after the start of the ketamine infusion on a 3 Tesla MR Scanner (MAGNETOM Prisma, Siemens Medical, Erlangen, Germany) using a 64-channel head/neck coil. Functional imaging data was acquired using a single-shot gradient-recalled echo-planar imaging (EPI) sequence with the following parameters: echo time (TE)/repetition time (TR) = 30 ms/1600 ms, matrix size of 70 × 70 voxels, a field of view (FOV) of 210 × 210 mm, and a multiband factor of 2. The resulting voxel dimensions were 3 × 3 × 3 mm, with a total of 48 slices (slice thickness = 3 mm). Preprocessing included conversion of data to NIfTI-format, physiological noise correction (PESTICA; [38]), motion correction (SLOMOCO; [39]), slice time correction (SPM12), wavelet despiking [40, 41], distortion correction (TOPUP; [42]), spatial normalization to Montreal Neurological Institute (MNI) space via the mean T1 image across sessions (SPM12), and spatial smoothing using a 9 mm full width at half maximum (FWHM) Gaussian kernel.

Functional image quality was visually assessed during data acquisition, and two participants were excluded because of severe motion-related image-quality impairment. No quantitative motion-based exclusion threshold was specified a priori. Because conventional volume-wise framewise displacement (FD) is not provided within SLOMOCO-based preprocessing, an approximate FD-equivalent motion metric was retrospectively derived from the SLOMOCO motion estimates. The median value per scan was used for descriptive characterization only and was not used to determine any additional exclusions.

### MID task

During the MID task, participants were instructed to maximize gains and avoid losses during win, loss, and neutral trials. Each trial began with the presentation of a cue (anticipation phase) indicating a potential gain or loss (e.g., –1€, +3€). After a variable delay of 3–5 s, a target stimulus appeared (“!”), prompting participants to press a button as quickly as possible. If the reaction time was within a predefined time window based on their individual average response time, as determined prior to the scan, the amount was gained or the loss was avoided; otherwise, the amount was not gained, or the loss was incurred. Each response was immediately followed by feedback displaying the outcome and the updated cumulative balance (outcome phase) (Supplementary Figure S1). Unknown to the participants, odds were manipulated during “win” and “loss” trials by increasing or decreasing the valid time window for reaction, respectively.

The task lasted 10 min and 5 s and consisted of nine blocks presented in randomized order (three gain, three loss, three neutral i.e., 0 € gambled), comprising a total of 57 trials (3 × 7 gain, 3 × 7 loss, 3 × 5 neutral). Trials were separated by a fixation cross presented for a variable interval of 3–7 s. To maintain attention and enable modeling of reward sensitivity, six different monetary amounts were used (0.5€, 1€, and 3€ for both gain and loss trials; initial balance: 10€). Monetary amounts were selected to reflect a range of low to high incentives, in line with previous studies on motivational salience [14, 17]. The final balance (if positive) was paid out to participants at the end of the study.

### Pharmacokinetic analysis

Racemic ketamine (0.5 mg/kg) was administered intravenously over 40 min, and blood samples were collected at predefined time points (0, 5, 10, 20, 40, 100, and 360 min). After centrifugation and plasma separation, plasma samples were stored at −80 °C until analysis. Ketamine plasma concentrations were determined by gas chromatography-tandem mass spectrometry with multiple reaction monitoring at the Department of Laboratory Medicine, Medical University of Vienna, Austria. The analytical procedure was validated in accordance with EMA guidelines [43].

Area under the curve (AUC) values for ketamine and its primary metabolite, norketamine, were calculated from model-fitted concentration-time profiles. MATLAB’s SimBiology toolbox was used to estimate individual concentration trajectories based on subject-specific pharmacokinetic (PK) parameters, enabling quantification of systemic drug exposure by accounting for inter-individual variability in drug distribution and clearance. The PK analysis was performed using a two-compartment model (central and peripheral) with two metabolic delay compartments to simulate the conversion of ketamine to norketamine. The model structure was based on the PK framework described by Kamp et al. [44], which applied a compartmental approach to account for the distribution and metabolism of ketamine and its metabolites. Parameter estimation was carried out using nonlinear mixed-effects modeling (NLME) in SimBiology (MATLAB). Individual PK parameters such as clearance (CL), volume of distribution (V_central_, V_peripheral_), and inter-compartmental transfer rates (Q) were estimated from the model based on the observed concentration-time data.

### fMRI analysis and statistical modeling

#### First level analysis

At the first level, each participant’s data was modeled using a general linear model (GLM). Pretrial cues (win/loss/neutral), targets, and feedback (gain/no gain/loss/no loss/neutral) were modeled separately. To control for residual physiological and other sources of noise, principal components of the white matter and cerebrospinal fluid (CSF) signal time courses were included as nuisance regressors (CompCor [45]).

Four canonical contrasts of the MID task were implemented to assess neural responses during the two primary phases of reward processing: anticipation and outcome evaluation (feedback). Anticipatory responses were assessed using the contrasts WinCue > NeutralCue and LossCue > NeutralCue. For outcome-related contrasts, trials were summarized into “Success” (gain/no loss) and “Failure” (no gain/loss) conditions. Outcome-related responses were examined using the feedback (FB) contrasts SuccessFB > NeutralFB and FailureFB > NeutralFB. These contrasts represent a standard analytical approach in fMRI studies using MID paradigms [15, 18, 24]. First-level contrast images for each participant were entered into second-level group analyses.

In order to capture neural processes associated with the alignment or mismatch between anticipated and actual outcomes [46, 47], we additionally implemented an Expectation-Outcome Congruency contrast. This contrast compared trials in which the actual feedback matched the expected outcome (congruent: win cue followed by win feedback, loss cue followed by loss feedback) versus trials where the outcome violated expectations (incongruent). At the first level, this contrast was specified as Congruent > Incongruent (GainFB + LossFB > NoGainFB + NoLossFB). We included this contrast to explore whether ketamine might influence neural responses related to expectancy-outcome alignment. To our knowledge, this specific Expectation-Outcome Congruency contrast has not previously been applied in the context of the MID task. Importantly, this contrast does not represent a formal reward prediction error signal [48], as the MID task does not model trial-by-trial learning.

#### Second level analysis

##### Whole-brain analysis

First-level contrasts (WinCue > NeutralCue, LossCue > NeutralCue, SuccessFB > NeutralFB, FailureFB > NeutralFB) were entered into second-level whole-brain analyses. To directly examine differences between ketamine and placebo sessions, paired t-tests were conducted for each MID task contrast. Furthermore, whole-brain analyses were performed using a flexible factorial design in SPM12. The model accounted for drug (placebo/ketamine), measurement, order of drugs (to account for potential carry-over and/or habituation effects), and enabled the testing of main and interaction effects using F- and t-tests. Whole-brain maps were thresholded at *p* < 0.001 (uncorrected) at the voxel level and evaluated using cluster-level family-wise error (FWE) correction at *p* < 0.05.

No formal a priori power calculation was performed. Sample size was based on previous ketamine fMRI studies using comparable crossover designs and MID task paradigms [13, 14].

##### Region of interest (ROI)-based analysis

To complement the whole-brain analyses, we conducted a literature-driven, a priori ROI-based analysis, focusing on key brain structures implicated in reward and salience processing, including the striatum, NAc, insula, amygdala, and thalamus [15, 46, 49–51]. ROIs were anatomically defined using the Automated Anatomical Labeling 3 (AAL3) atlas and analyzed bilaterally [52]. For each ROI, contrast estimates (beta values of the first level analysis) were extracted and analyzed using linear mixed-effects models in MATLAB to assess the influence of drug condition (ketamine vs. placebo), individual ketamine exposure (modeled as AUC), and subjective drug experience (5D-ASC scores).

The primary model specification was:

$${\beta }_{{ROI}} \sim 	 \, {dru}{g}_{{ketamine},{placebo}}+{ketLv}{l}_{{AUC}{to}{MID}/2}+{se}{x}_{m,f}+{age} \\ 	+{measuremen}{t}_{1,2}+{drugOrde}{r}_{1,2}+{fiveDASC}+(1\,|\,{subject})$$

Here, ketLvl represents the area under a piecewise linear approximation of individual ketamine exposure up to half the task duration (z-scored within the ketamine condition, otherwise zero, to avoid collinearity with the drug conditions). fiveDASC reflects subjective drug experience (5D-ASC, z-scored within each condition). A random intercept for subject was included to account for repeated measures. ROI effects were considered significant if they survived false discovery rate (FDR) correction for multiple comparisons according to Benjamini and Hochberg (q < 0.05).

The Expectation-Outcome Congruency contrast (Congruent > Incongruent) was analyzed analogously to the canonical MID task contrasts using whole-brain flexible factorial models and ROI-based linear mixed-effects models.

##### Post-hoc sensitivity analysis

Whole-brain models estimated 322–366 resolution elements for the calculated contrasts, corresponding to adjusted type-I error probabilities ranging from 1.6×10⁻⁴–1.4×10⁻⁴ at the voxel level. With 28 participants, six covariates, a power of 0.80, and an assumed reliability of r = 0.21 [53], detectable effect sizes were estimated at approximately f = 0.69 (d = 1.38) under repeated-measures ANOVA assumptions. Under the same assumptions, testing a single ROI would require a detectable effect size of approximately f = 0.37 (d = 0.74). Because multiple models and contrasts were evaluated, these estimates should be interpreted as approximate indicators of effect size sensitivity. Effect size calculations were performed using G*Power 3.1.9.7 (ANOVA: repeated measures, within factors).

## Results

A total of 36 healthy subjects completed the study. During data acquisition, two subjects were flagged due to severe motion-related image quality impairment during MRI scanning. After excluding these subjects, as well as participants with missing plasma concentration values or irregular ketamine plasma concentration-time curves (e.g., peak plasma concentration 20 min prior to end of infusion), 28 participants (15 females, mean age 23.6 ± 3.8 years) remained eligible for analysis. Due to incomplete 5D-ASC data in one participant, ROI-based linear mixed-effects analyses including subjective drug effects were conducted in 27 participants.

### Pharmacokinetic data

**Fig. 2:**
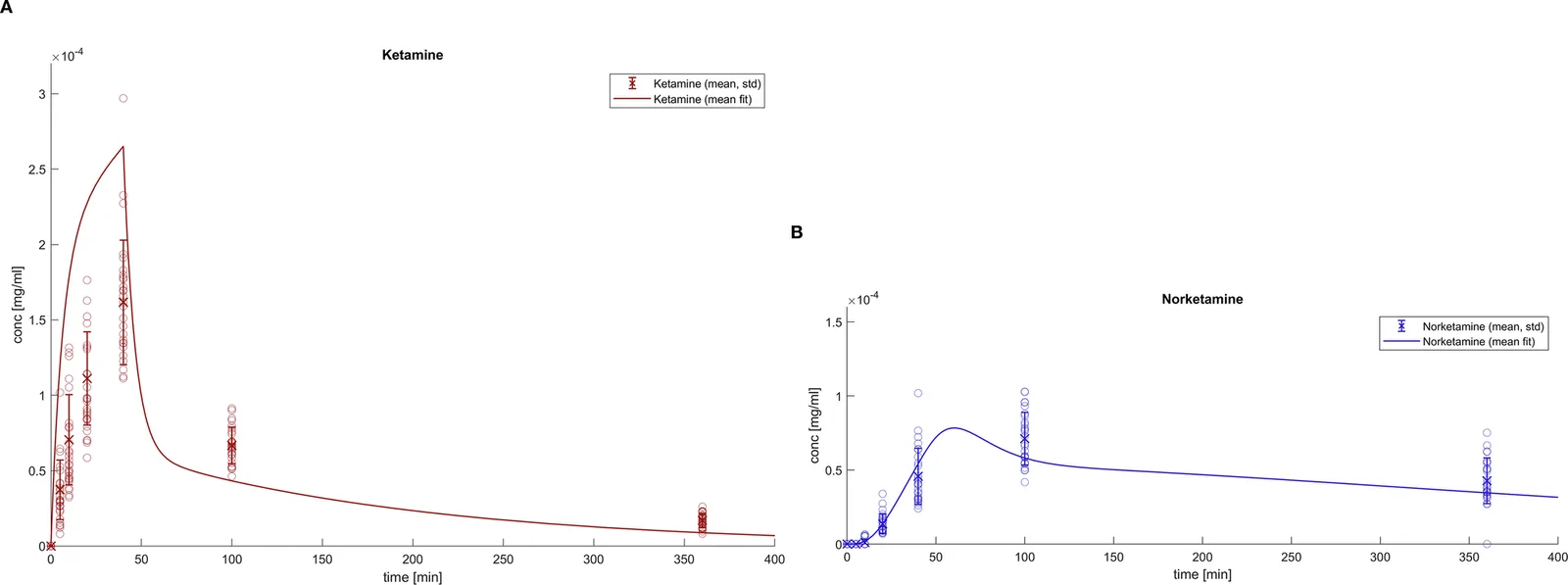
Ketamine and norketamine plasma concentration–time curves. Piece-wise, linearly fitted plasma concentration–time curves for ketamine (**A**, red) and norketamine (**B**, blue) following a 40 min intravenous infusion of racemic ketamine (0.5 mg/kg). Symbols (open circles) represent individual data points and mean ± standard deviation (crosses with error bars), while solid lines depict the pooled pharmacokinetic model fit.

Ketamine plasma levels increased rapidly after infusion, peaking at approximately 40 min, i.e., end of infusion (Figure S2A, Table S1), while norketamine concentrations increased more slowly, consistent with the expected metabolic conversion over time (Figure S2B, Table S2). AUC values for ketamine concentration-time curves were calculated up to half the task duration (5 h 14 min ± 6 min) using a two-compartment PK model (SimBiology, MATLAB) and model-fitted concentration-time profiles (Fig. 2).

### Subjective effects

**Fig. 3:**
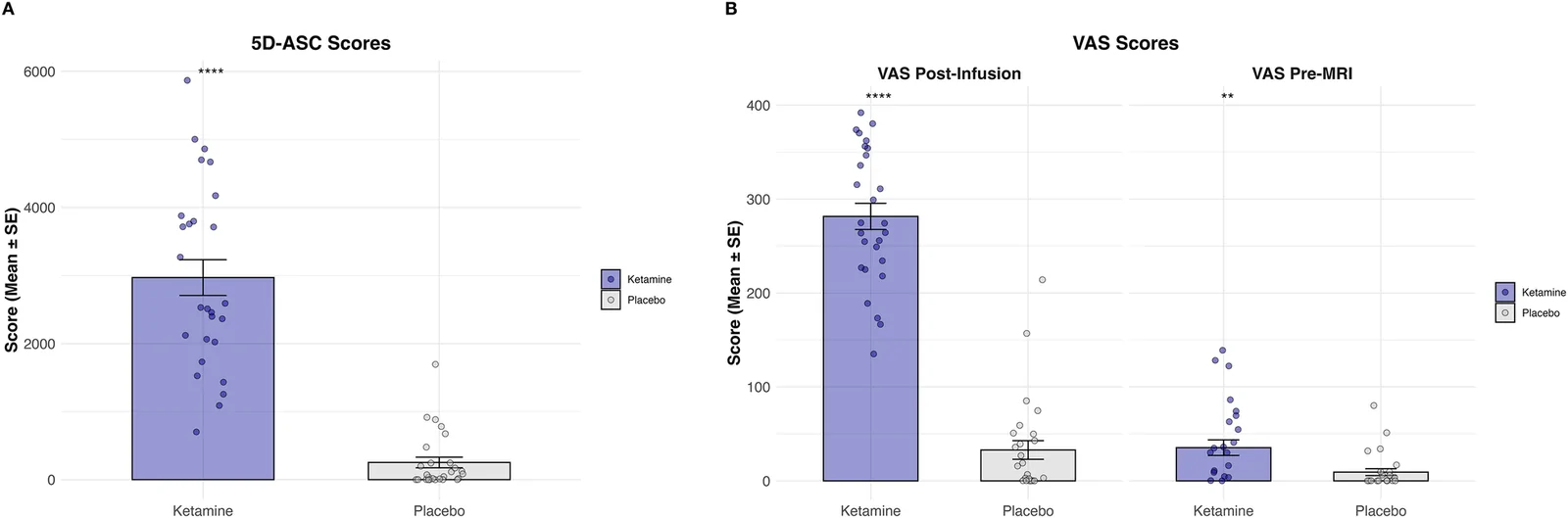
Subjective drug effects. Subjective drug effects measured via the 5-dimensional altered states of consciousness rating scale (5D-ASC) questionnaire (**A**) and visual analog scale (VAS) ratings of common ketamine-associated adverse effects (**B**) (*n* = 27). Total 5D-ASC scores and VAS ratings were obtained immediately after infusion stop (post-infusion), with VAS ratings additionally assessed immediately before MRI scanning (pre-MRI). Symptoms included dizziness, blurred vision, headache, nausea, dry mouth, poor coordination, and restlessness [3]. Bars represent mean ± standard deviation. Asterisks indicate significant differences between conditions (**p < 0.01, ****p < 0.0001). Group differences were tested with paired t-tests (5D-ASC Scores, VAS Post-Infusion) or Wilcoxon signed-rank tests (VAS Pre-MRI).

5D-ASC and VAS scores were available for 27 of the 28 subjects. Values are reported as mean ± standard deviation unless otherwise specified. Total 5D-ASC scores were significantly elevated during ketamine visits (2969.8 ± 1363.3) compared to placebo visits (251.6 ± 406.1; paired t(26) = 10.09, *p* < 0.0001, Cohen’s d = 2.67; Fig. 3A), indicating pronounced subjective drug effects during ketamine sessions. VAS scores immediately post-infusion were also significantly higher (281.5 ± 72.2) compared to placebo (32.8 ± 51.5; paired t(26) = 16.49, *p* < 0.0001, Cohen’s d = 3.93). During ketamine sessions, VAS scores decreased substantially from immediately post-infusion (281.5 ± 72.2) to just before MRI scanning (35.3 ± 43; paired t(26) = 18.5, *p* < 0.0001, Cohen’s d = 4). Nevertheless, compared to placebo sessions (9.3 ± 19.1), VAS scores just before MRI remained significantly higher during ketamine sessions (median difference = 26; Wilcoxon signed-rank test: V = 189, *p* = 0.0018; Fig. 3B). Distributional checks supported the use of paired t-tests for 5D-ASC and VAS post-infusion, while non-normal pre-MRI VAS differences were analyzed with the Wilcoxon signed-rank test. Descriptive statistics for the five canonical 5D-ASC dimensions (Oceanic Boundlessness, Anxious Ego Dissolution, Visionary Restructuralization, Auditory Alterations, and Reduction of Vigilance) are provided in the Supplementary Material (Table S3, Figure S3).

### Functional unblinding

In the ketamine condition, 26 out of 28 participants provided a response, and all responding participants correctly identified the treatment they had received. In the placebo condition, 24 out of 28 participants (85.7%) correctly guessed their treatment allocation, while 2 (7.1%) guessed incorrectly and 2 (7.1%) reported being unable to decide. Confidence ratings were high in both conditions (mean ± SD: 94.12 ± 9.17 for ketamine; 89.53 ± 13.97 for placebo).

### MID task performance

Reaction times during the MID task did not significantly differ (α = 0.05) between ketamine and placebo (${\bar{{t}_{placebo}}} \approx 338\,{ms}$, ${\bar{{t}_{ketamine}}} \approx 352\,{ms}$, *p* = 0.07, paired t-test; ${\bar{{balance}_{placebo}}} \approx 15.82$ €, ${\bar{{balance}_{ketamine}}} \approx 15.51$ €, *p* = 0.78, paired t-test) (Supplementary Figure S4). Normality tests confirmed that assumptions for the paired t-test were met (all *p* > 0.05).

### fMRI analysis

**Fig. 4:**
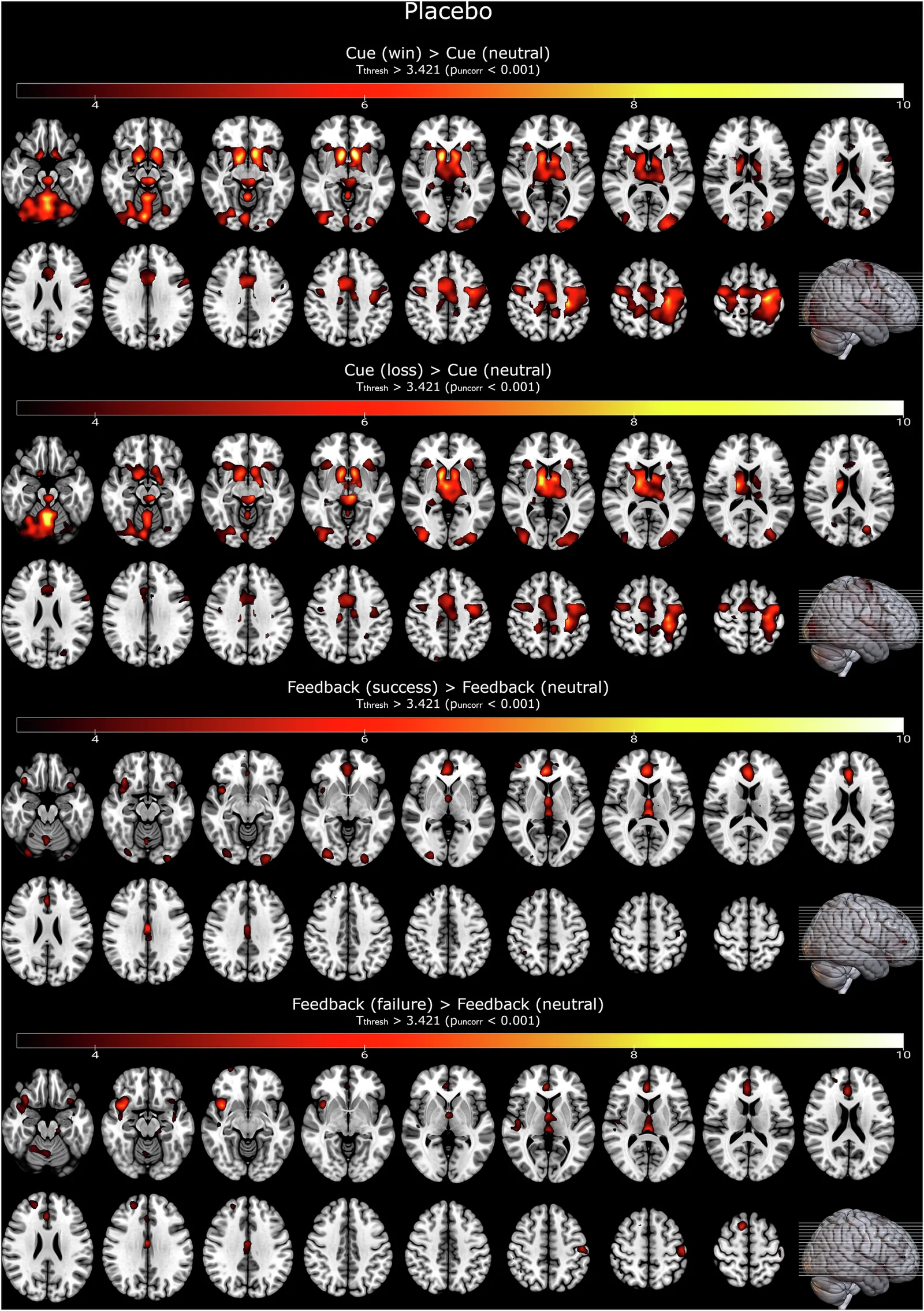
Whole-brain activation during the Monetary Incentive Delay (MID) task for the placebo condition. Group-level whole-brain activation maps for canonical MID contrasts are shown for reward anticipation (WinCue > NeutralCue), loss anticipation (LossCue > NeutralCue), success feedback (SuccessFB > NeutralFB), and failure feedback (FailureFB > NeutralFB). Statistical maps are thresholded at *p* < 0.001 (uncorrected) for visualization.

At the group level, the MID task contrasts elicited robust activation patterns consistent with previous literature [15, 17, 18] (Fig. 4). Reward anticipation (WinCue > NeutralCue) was associated with activation in the ventral striatum (including NAc), dorsal striatum, anterior insula, medial frontal regions (supplementary motor area (SMA), dorsal anterior cingulate cortex (dACC)), as well as parietal, occipital and cerebellar regions (peak T = 10.90, k = 6059, pFWE < 0.001). Loss anticipation (LossCue > NeutralCue) produced a broadly similar activation pattern, involving ventral and dorsal striatum, anterior insula, medial frontal regions (SMA, dACC), and parietal, occipital, and cerebellar regions (peak T = 10.75, k = 4616, pFWE < 0.001). Success feedback (SuccessFB > NeutralFB) was primarily associated with activation in the ventromedial prefrontal cortex (vmPFC), midline thalamic regions, and occipital cortex (peak T = 7.44, k = 531, pFWE = 0.001), whereas failure feedback showed activation in the anterior insula, vmPFC, and midline thalamic regions (peak T = 7.38, k = 257, pFWE = 0.001).

#### Whole-brain analysis

**Fig. 5:**
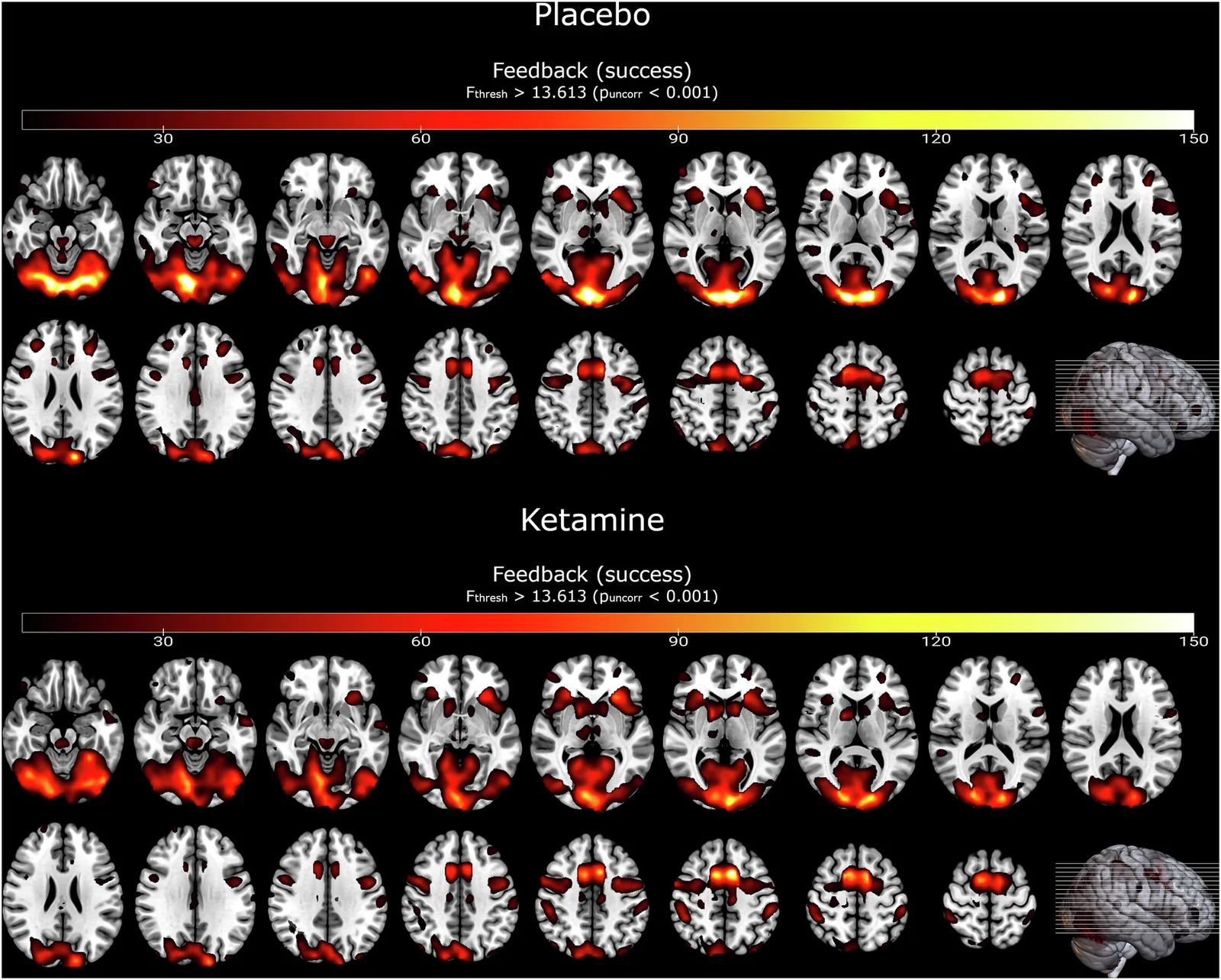
Whole-brain effects during success feedback under placebo and ketamine. Group-level whole-brain F-statistic maps are shown separately for the placebo (top panel) and ketamine (bottom panel) conditions. Statistical maps are thresholded at *p* < 0.001 (uncorrected) for visualization (F > 13.613). In both conditions, spatially coherent effects are observed.

Whole-brain analyses did not reveal significant drug-related effects for any MID task contrast. Neither the main effect of drug condition (ketamine vs placebo) nor exploratory F-contrasts for other factors revealed significant differences in BOLD activation (cluster-level pFWE > 0.05; Supplementary Table S4). Paired t-tests comparing ketamine and placebo sessions likewise did not reveal significant differences in BOLD activation (cluster-level pFWE > 0.05; Supplementary Table S5). However, when exploring each substance condition individually, spatially coherent activation patterns consistent with typical MID task responses were observed (Fig. 5).

#### ROI-based analysis

Linear mixed-effects models were conducted for each ROI across the four canonical MID task contrasts (WinCue > NeutralCue, LossCue > NeutralCue, SuccessFB > NeutralFB, and FailureFB > NeutralFB). No main effects of drug condition survived FDR correction for multiple comparisons. Reported *p*-values are uncorrected unless stated otherwise. An uncorrected thalamic effect was observed during loss anticipation (β = 0.194, SE = 0.095, t = 2.04, *p* = 0.047). Individual ketamine exposure (ketLvl) was not associated with BOLD activation in any ROI across contrasts (all *p* ≥ 0.05).

#### Reward anticipation (WinCue > NeutralCue)

No significant drug effects were observed in the striatum (β = 0.031, SE = 0.078, t = 0.40, *p* = 0.690), nucleus accumbens (β = 0.173, SE = 0.135, t = 1.28, *p* = 0.206), insula (β = 0.085, SE = 0.186, t = 0.46, p = 0.651), thalamus (β = 0.054, SE = 0.113, t = 0.48, *p* = 0.636), or amygdala (β = 0.229, SE = 0.183, t = 1.25, *p* = 0.216). No significant associations between subjective drug experience (5D-ASC scores) and ROI activation were observed.

#### Loss anticipation (LossCue > NeutralCue)

No significant drug effects were observed in the striatum (β = −0.017, SE = 0.097, t = −0.17, *p* = 0.864), nucleus accumbens (β = 0.229, SE = 0.140, t = 1.63, *p* = 0.109), insula (β = 0.136, SE = 0.185, t = 0.74, p = 0.464), or amygdala (β = 0.182, SE = 0.175, t = 1.04, p = 0.304). In the thalamus, ketamine was associated with greater activation compared to placebo (β = 0.194, SE = 0.095, t = 2.04, p = 0.047). Additionally, a positive association between subjective drug experience (5D-ASC scores) and nucleus accumbens activation was observed (β = 0.036, SE = 0.017, t = 2.14, p = 0.038).

#### Success feedback (SuccessFB > NeutralFB)

For success feedback, no significant drug effects were observed in the striatum (β = 0.096, SE = 0.190, t = 0.50, p = 0.617), nucleus accumbens (β = 0.146, SE = 0.237, t = 0.61, p = 0.542), insula (β = 0.020, SE = 0.383, t = 0.05, p = 0.958), thalamus (β = −0.175, SE = 0.157, t = −1.11, p = 0.271), or amygdala (β = −0.210, SE = 0.301, t = −0.70, p = 0.489). No significant associations with subjective drug effects were observed (all p ≥ 0.05).

#### Failure feedback (FailureFB > NeutralFB)

For failure feedback, no significant drug effects were observed in the striatum (β = 0.150, SE = 0.182, t = 0.82, *p* = 0.415), nucleus accumbens (β = 0.019, SE = 0.294, t = 0.07, *p* = 0.948), insula (β = −0.003, SE = 0.348, t = −0.01, *p* = 0.993), thalamus (β = −0.235, SE = 0.156, t = −1.50, *p* = 0.140), or amygdala (β = −0.163, SE = 0.306, t = −0.53, *p* = 0.596). No significant associations with subjective drug effects were observed (all *p* ≥ 0.05).

Across all ROI analyses, no effects survived correction for multiple comparisons. Full linear mixed-effects model results are reported in Supplementary Table S6. To exclude a potential influence of monetary amount in each trial, win and loss trials were additionally modeled using parametrization and analyses were repeated on an exploratory basis. Qualitatively, no differences in substance effects compared to the initial model were observed, thus only results of the non-parametrized model are reported. Exploratory supplementary analyses examining individual 5D-ASC subdimensions in relation to ROI activation measures revealed only isolated associations that did not survive correction for multiple comparisons. The complete linear mixed-effects results for all 5D-ASC subdimensions, contrasts, and ROIs are provided in a separate Supplementary Appendix (Supplementary Appendix 1).

#### Expectation-outcome congruency contrast

Analyses of the Expectation-Outcome Congruency contrast (Congruent > Incongruent) did not reveal significant drug-related effects at the whole-brain or ROI level (cluster-level pFWE > 0.05). Across conditions, the contrast primarily elicited activation in occipital visual regions (Supplementary Figure S5).

#### Head motion

To transparently characterize head motion, we retrospectively examined scan-level median values of a SLOMOCO-derived approximate FD-equivalent motion metric. Scan-level median FD-equivalent values were comparable between ketamine (mean ± SD: 0.121 ± 0.038 mm) and placebo (0.120 ± 0.045 mm), as well as between the first (0.120 ± 0.044 mm) and second measurement (0.122 ± 0.039 mm). Wilcoxon signed-rank tests indicated no significant differences between ketamine and placebo or between M1 and M2 (both *p* > 0.05). Distributions by treatment condition and measurement session are presented in Supplementary Figure S6, with the underlying scan-level values provided in Supplementary Table S7.

## Discussion

Ketamine administration 5 h prior to scanning did not significantly alter reward-related brain activation during the MID task at either the ROI or whole-brain level. This absence of detectable effects may partly reflect limited sensitivity of canonical MID task contrasts to subtle drug-related changes during the subacute phase, rather than indicating an absence of ketamine-related neuromodulatory processes per se.

Canonical MID task contrasts (WinCue > NeutralCue, LossCue > NeutralCue, SuccessFB > NeutralFB, FailureFB > NeutralFB) elicited robust activation patterns consistent with meta-analytic findings [15, 17, 18]. Reward and loss anticipation contrasts engaged the ventral striatum, thalamus, anterior insula, and medial frontal regions, while feedback contrasts recruited medial frontal and posterior cortical areas. Despite coherent task-related activation patterns, whole-brain analyses using flexible factorial models and paired t-tests showed no significant differences between ketamine and placebo for any MID task contrast. Likewise, ROI-based analyses did not reveal a significant main effect of drug condition in the striatum, NAc, thalamus, insula, and amygdala. These ROIs were selected a priori based on meta-analytic evidence identifying them as core neural substrates of reward and loss anticipation during the MID task [15, 46, 49–51].

Although no ROI effects survived correction for multiple comparisons, ROI-based analyses revealed isolated uncorrected associations. Ketamine was associated with increased thalamic activation during loss anticipation, while higher subjective drug effects (5D-ASC scores) were associated with greater NAc activation during loss anticipation. Meta-analytic findings from the MID task indicate increased dorsal-medial thalamic activation during loss versus reward anticipation, potentially reflecting the prediction of aversive outcomes [15]. Moreover, ketamine has previously been associated with altered thalamo-cortical connectivity, thalamic volumetric changes, and increased thalamic BOLD activation in healthy participants [54–56], suggesting that the thalamus may be sensitive to ketamine-induced modulation of salience and motivational processing. In this context, the observed increase in thalamic activation under ketamine during loss anticipation may tentatively align with previous findings implicating the thalamus as a ketamine-sensitive region. However, these associations did not survive correction for multiple comparisons and should therefore be interpreted with utmost caution.

In addition to canonical MID task contrasts, we also explored an Expectation-Outcome Congruency contrast to assess whether ketamine modulated neural responses when outcomes matched or violated prior cue-based expectations. This contrast primarily engaged occipital visual regions (Figure S5). No significant drug-related effects were observed for this contrast at the ROI or whole-brain level. Since this contrast compared expected with unexpected outcomes irrespective of valence, it does not isolate a classical reward prediction error signal. Incongruent trials comprised both worse-than-expected outcomes (no gain after gain cue) and better-than-expected outcomes (no loss after loss cue), which may generate signals of opposite sign and reduce sensitivity to typical prediction-error responses. The predominance of occipital activation may instead reflect visual-attentional processing of expectancy-outcome alignment during feedback evaluation.

When combined with previous studies, our findings provide further information on the temporal effects of ketamine on reward processing. Kotoula et al. reported increased activation of the NAc, putamen, insula, and caudate in remitted MDD patients approximately 2 h post-infusion during the feedback phase of the MID task, though only in ROI analyses [14]. François et al. reported an attenuated ventral striatal response during reward anticipation in both humans and rodents within 40 min post-infusion [13]. While these findings may initially appear divergent, they likely reflect differences in timing. Both studies assessed participants in the acute post-infusion phase (40 min and 2 h post-administration), when plasma concentrations of ketamine and norketamine were near their peak levels and NMDA receptor antagonism ongoing [44, 57]. François et al. captured the acute phase near peak NMDA receptor blockade, whereas Kotoula et al. examined a slightly later window when acute psychotomimetic effects had subsided but receptor-level modulation was likely ongoing [31, 44, 57, 58].

Our scanning time point (~5 h post-infusion) corresponds to a subacute pharmacodynamic phase, occurring after the resolution of acute psychotomimetic symptoms [59, 60] and temporally overlapping with the onset of antidepressant effects in MDD [1, 2]. This interval is hypothesized to reflect a transitional window between acute receptor-level effects and more sustained synaptic and circuit-level adaptations. Preclinical work suggests that this phase may involve glutamate-dependent synaptic strengthening and network reorganization, mediated by downstream mechanisms such as AMPA receptor activation, brain-derived neurotrophic factor (BDNF) release, and mammalian target of rapamycin (mTOR) signaling [31, 32, 57, 58, 61]. Importantly, the present study does not provide direct evidence of these molecular or synaptic mechanisms, and their discussion here is intended solely to contextualize the chosen time window.

Pharmacokinetic and pharmacodynamic studies indicate that ketamine’s NMDA receptor antagonism peaks rapidly after administration and declines over several hours, consistent with a plasma elimination half-life of approximately 2–4 h [28, 29]. Preclinical evidence suggests that ketamine may continue to block NMDA receptors for up to 24 h due to trapping mechanisms [62]. Norketamine is eliminated more slowly and maintains high plasma concentrations more than 5 h after infusion (Fig. 2B) [28, 63]. By the time of our scan (~5 h post-infusion), NMDA receptor blockade has likely subsided [44, 57], and secondary metabolites such as hydroxynorketamine predominate [57]. Although these metabolites are implicated in the sustained antidepressant effects of ketamine [58], their immediate impact on task-evoked BOLD responses during reward processing remains unclear [35, 58]. In our study, pharmacokinetic modeling yielded ketamine and norketamine concentration-time profiles consistent with expected pharmacokinetic patterns, yet individual ketamine exposure (AUC) was not significantly associated with task-evoked brain activation.

The temporal mismatch between the peak of receptor-level effects and our task window may account for the absence of robust effects in our dataset. Ketamine’s effects on reward circuitry in healthy subjects may be time-specific, with fMRI alterations most reliably detected during the acute post-infusion window (<2 h) when NMDA receptor antagonism and dissociative effects are most pronounced [13, 14]. The absence of detectable modulation of task-evoked reward responses at ~5 h may itself be informative, suggesting that ketamine’s influence on reward-related neural signals may shift from immediate receptor-level modulation toward processes that are not readily captured by canonical MID contrasts during this subacute window. By approximately 5 h, when pharmacokinetics shift toward downstream metabolites and behavioral normalization occurs, ketamine’s impact on reward processing may be too subtle to detect under the presented methodological conditions, potentially manifesting through longer-term plasticity mechanisms rather than immediate modulation of task-related neural activity [31, 32, 57, 58, 61]. This intermediate, subacute state may reflect a transitional pharmacodynamic window between acute receptor-level effects and sustained synaptic changes; however, this interpretation remains speculative and should be tested in studies with more comprehensive sampling and imaging approaches. Future studies using denser temporal sampling across acute (≤2 h), intermediate (3–6 h), and later phases (24–72 h) could map evolving neural effects of ketamine more precisely.

It is also conceivable that the absence of ketamine-induced neural effects in our healthy cohort may result from baseline differences from clinical samples, particularly MDD patients [14]. MDD is associated with deficits in reward processing, including attenuated striatal and prefrontal responses [9, 64], and ketamine may differently modulate reward processing in individuals with pre-existing reward dysfunction. Indeed, ketamine-induced increases in striatal activation have been reported in depressed individuals [14, 35], whereas attenuation of ventral striatal response during reward anticipation under ketamine was observed in healthy subjects [13]. Mkrtchian et al. further demonstrated diagnosis-specific connectivity changes 48 h post-infusion, with ketamine normalizing fronto-striatal connectivity in MDD but shifting it in healthy controls toward patterns typical of unmedicated MDD [5]. These divergent effects suggest that ketamine may restore dysfunctional circuits in depression but disrupt the same networks in healthy individuals, consistent with state-dependent modulation [5, 8]. Evidence indicates that ketamine transiently induces symptoms related to impaired motivation in healthy participants [65, 66], further implicating that the direction of ketamine’s effects on reward circuitry may be state-dependent.

Several limitations should be considered when interpreting these findings. First, our study included healthy subjects only and did not assess symptom-level constructs related to reward dysfunction such as anhedonia. The absence of significant substance effects on MID task-related activation may not translate to clinical populations with pre-existing reward dysfunction, such as MDD patients. Second, the timing of the fMRI task (~5 h post-infusion) could have missed the window in which ketamine exerts its most pronounced effects on reward processing. Although this time point was chosen to investigate subacute changes relevant to sustained antidepressant mechanisms, it may have fallen into a transitional pharmacodynamic state in which neither acute pharmacological effects nor long-term synaptic changes were sufficiently pronounced to produce detectable BOLD signal differences. Denser temporal sampling across acute, intermediate, and later phases would be necessary to fully capture the evolving neural effects of ketamine.

Third, the Expectation-Outcome Congruency contrast should be interpreted cautiously. While this contrast captures whether outcomes are congruent or incongruent with prior cues, the MID task was not designed to estimate computational reward prediction error, and we did not model trial-wise learning signals. Accordingly, this contrast does not represent a formal reward prediction error signal [48]. Fourth, functional unblinding due to subjective ketamine effects, as confirmed by post-session treatment guesses, may have influenced task engagement or expectancy. The high accuracy and confidence of correct treatment identification indicate substantial functional unblinding, which is widely discussed as a limitation in ketamine studies due to its distinct dissociative and psychotomimetic properties, but is only rarely systematically reported [1–3]. Fifth, no quantitative threshold for motion-based exclusion was specified a priori, and the original exclusions were based on visual assessment of motion-related image-quality impairment. This observer-dependent procedure may have introduced selection bias, and residual motion may have affected BOLD estimates, although the SLOMOCO-derived approximate FD-equivalent motion metric did not differ significantly between ketamine and placebo. Furthermore, while we modeled individual ketamine and norketamine exposure using AUC values, we did not directly assess plasma concentrations of downstream metabolites such as hydroxynorketamine at the time of scanning. Additionally, AUC values may not fully capture the complexity of ketamine’s pharmacokinetics and pharmacodynamics, particularly when modeling individual subjective or neural responses. Finally, limitations in effect size sensitivity should be acknowledged. While the applied statistical thresholds made it unlikely that effects would be detected at the voxel level, the usually more liberal cluster-level or ROI analyses might have detected sufficiently strong neural effects of ketamine. We did not perform formal equivalence testing and therefore cannot formally rule out small effects.

In summary, we observed no significant drug-related effects on reward processing during the MID task in healthy participants 5 h after ketamine infusion. These findings primarily indicate an absence of detectable task-evoked effects under the present methodological conditions. When considered alongside prior work, they suggest that ketamine’s effects on reward circuitry may be time-sensitive and potentially most prominent during the acute post-infusion phase (<2 h) [13, 14, 35]. The 5 h post-infusion time point may reflect a transitional pharmacodynamic window, in which receptor-level effects have diminished but sustained synaptic changes have not yet fully developed. In this state, ketamine’s influence on anticipation- and feedback-related activation, as well as the Expectation-Outcome Congruency contrast, may be too subtle to detect with present methods. Moreover, ketamine’s neural effects may be state-dependent, with more robust modulation emerging in individuals with pre-existing reward system dysfunction, such as MDD patients [14, 34, 35]. Future studies should integrate longitudinal designs with denser temporal sampling across acute, intermediate, and later phases, and explicitly compare healthy and clinical cohorts to further characterize temporal dynamics and state-dependence of ketamine’s effects on reward processing.

## Supplementary information

Supplement

Supplementary Appendix 1

## Data Availability

The data that support the findings of this study, excluding data enabling identification of study participants, are available from the corresponding author upon reasonable request.
