# Supplementary material for: Subacute effects of ketamine on neural correlates of reward processing
Source: Transl Psychiatry. 2026 Sep 25;16:521. doi: 10.1038/s41398-026-04485-6 (PMC13639076; doi:10.1038/s41398-026-04485-6)
Supplement: Supplementary file 1 [file 41398_2026_4485_MOESM2_ESM.pdf]

Contrast: cue win vs cue neutral   ROI: striatum 5D-ASC subscore: asc_obn

lme =

Linear mixed-effects model fit by REML

Model information:

Number of observations	54
Fixed effects coefficients	8
Random effects coefficients	27
Covariance parameters	2

Formula:

ROI ~ 1 + measurement + drug + ketLvl + sex + age + drugOrder + fiveDASC + (1 | subject)

Model fit statistics:

AIC	BIC	LogLikelihood	Deviance
-47.978	-29.691	33.989	-67.978

Fixed effects coefficients (95% CIs):

Name	Estimate	SE	tStat	DF	pValue	Lower
'(Intercept)'	0.072804	0.059623	1.2211	46	0.22828	-0.047211
'measurement'	-0.0095417	0.014049	-0.67917	46	0.50043	-0.037821
'drug'	0.0277	0.077536	0.35725	46	0.72254	-0.12837
'ketLvl'	-1.4389e-06	3.7289e-06	-0.38587	46	0.70137	-8.9447e-06
'sex'	0.026564	0.033067	0.80334	46	0.42591	-0.039996
'age'	-0.017688	0.022264	-0.79447	46	0.431	-0.062502
'drugOrder'	0.0039312	0.031682	0.12408	46	0.90179	-0.059841
'fiveDASC'	-0.012104	0.0090044	-1.3443	46	0.18546	-0.030229

Upper
0.19282
0.018738
0.18377
6.067e-06
0.093123
0.027126
0.067703
0.0060207

Random effects covariance parameters (95% CIs):

Group: subject (27 Levels)

Name1	Name2	Type	Estimate	Lower	Upper
'(Intercept)'	'(Intercept)'	'std'	0.072291	0.049449	0.10568

Group: Error

Name	Estimate	Lower	Upper
'Res Std'	0.049313	0.03668	0.066297

Contrast: cue win vs cue neutral   ROI: striatum 5D-ASC subscore: asc_aed

lme =

Linear mixed-effects model fit by REML

Model information:

Number of observations	54
Fixed effects coefficients	8
Random effects coefficients	27
Covariance parameters	2

Formula:

ROI ~ 1 + measurement + drug + ketLvl + sex + age + drugOrder + fiveDASC + (1 | subject)

Model fit statistics:

AIC	BIC	LogLikelihood	Deviance
-46.298	-28.012	33.149	-66.298

Fixed effects coefficients (95% CIs):

Name	Estimate	SE	tStat	DF	pValue	Lower
'(Intercept)'	0.068357	0.060251	1.1345	46	0.26245	-0.052922
'measurement'	-0.0049338	0.014275	-0.34562	46	0.7312	-0.033668
'drug'	0.032336	0.079693	0.40576	46	0.6868	-0.12808

'ketLvl'	-1.6568e-06	3.8325e-06	-0.43229	46	0.66755	-9.3712e-06
'sex'	0.025043	0.033389	0.75004	46	0.45705	-0.042165
'age'	-0.019337	0.022507	-0.85915	46	0.39471	-0.064641
'drugOrder'	0.0024814	0.032141	0.077202	46	0.9388	-0.062216
'fiveDASC'	-0.0016969	0.0094831	-0.17894	46	0.85877	-0.020785

Upper
0.18964
0.023801
0.19275
6.0577e-06
0.092251
0.025967
0.067179
0.017392

Random effects covariance parameters (95% CIs):
Group: subject (27 Levels)

Name1	Name2	Type	Estimate	Lower	Upper
'(Intercept)'	'(Intercept)'	'std'	0.072674	0.049534	0.10662

Group: Error

Name	Estimate	Lower	Upper
'Res Std'	0.050705	0.037732	0.068138

Contrast: cue win vs cue neutral ROI: striatum 5D-ASC subscore: asc_vis

lme =

Linear mixed-effects model fit by REML

Model information:

Number of observations	54
Fixed effects coefficients	8
Random effects coefficients	27
Covariance parameters	2

Formula:
ROI ~ 1 + measurement + drug + ketLvl + sex + age + drugOrder + fiveDASC + (1 | subject)

Model fit statistics:

AIC	BIC	LogLikelihood	Deviance
-47.11	-28.824	33.555	-67.11

Fixed effects coefficients (95% CIs):

Name	Estimate	SE	tStat	DF	pValue	Lower
'(Intercept)'	0.072478	0.059333	1.2216	46	0.2281	-0.046953
'measurement'	-0.009898	0.015143	-0.65363	46	0.51661	-0.04038
'drug'	0.029747	0.079256	0.37532	46	0.70914	-0.12979
'ketLvl'	-1.5394e-06	3.8111e-06	-0.40392	46	0.68814	-9.2107e-06
'sex'	0.025934	0.032723	0.79254	46	0.43212	-0.039933
'age'	-0.018444	0.021997	-0.8385	46	0.40608	-0.062722
'drugOrder'	0.0046189	0.031405	0.14707	46	0.88372	-0.058597
'fiveDASC'	-0.008827	0.0093134	-0.94777	46	0.3482	-0.027574

Upper
0.19191
0.020584
0.18928
6.132e-06
0.091801
0.025833
0.067835
0.0099199

Random effects covariance parameters (95% CIs):
Group: subject (27 Levels)

Name1	Name2	Type	Estimate	Lower	Upper
'(Intercept)'	'(Intercept)'	'std'	0.070702	0.047606	0.105

Group: Error

Name	Estimate	Lower	Upper
'Res Std'	0.050844	0.037691	0.068588

```
 Contrast: cue win vs cue neutral       ROI: striatum 5D-ASC subscore: asc_aud

lme =


Linear mixed-effects model fit by REML

Model information:
    Number of observations              54
    Fixed effects coefficients           8
    Random effects coefficients         27
    Covariance parameters                2

Formula:
    ROI ~ 1 + measurement + drug + ketLvl + sex + age + drugOrder + fiveDASC + (1 | subject)

Model fit statistics:
    AIC        BIC        LogLikelihood    Deviance
    -47.965    -29.679    33.983           -67.965

Fixed effects coefficients (95% CIs):
    Name                 Estimate      SE            tStat       DF    pValue     Lower
    '(Intercept)'          0.075692      0.059493      1.2723    46    0.20967      -0.044062
    'measurement'        -0.0090576      0.014113    -0.64177    46    0.52421      -0.037466
    'drug'                 0.018482      0.078745      0.2347    46    0.81548       -0.14002
    'ketLvl'             -9.881e-07    3.7873e-06     -0.2609    46    0.79533    -8.6115e-06
    'sex'                  0.024689      0.032826     0.75213    46    0.45581      -0.041386
    'age'                 -0.021003      0.022078     -0.9513    46    0.34642      -0.065443
    'drugOrder'           0.0016467      0.031412    0.052421    46    0.95842      -0.061582
    'fiveDASC'            -0.012328     0.0094617     -1.3029    46    0.19909      -0.031373


    Upper
       0.19545
      0.019351
       0.17699
    6.6353e-06
      0.090765
      0.023438
      0.064875
     0.0067175

Random effects covariance parameters (95% CIs):
Group: subject (27 Levels)
    Name1                Name2                Type         Estimate    Lower       Upper
    '(Intercept)'        '(Intercept)'        'std'        0.071477    0.048695    0.10492

Group: Error
    Name             Estimate    Lower       Upper
    'Res Std'        0.049774    0.037037    0.066891


 Contrast: cue win vs cue neutral       ROI: striatum 5D-ASC subscore: asc_vig

lme =


Linear mixed-effects model fit by REML

Model information:
    Number of observations              54
    Fixed effects coefficients           8
    Random effects coefficients         27
    Covariance parameters                2

Formula:
    ROI ~ 1 + measurement + drug + ketLvl + sex + age + drugOrder + fiveDASC + (1 | subject)

Model fit statistics:
    AIC        BIC        LogLikelihood    Deviance
    -46.422    -28.135    33.211           -66.422

Fixed effects coefficients (95% CIs):
    Name                 Estimate      SE            tStat       DF    pValue     Lower
    '(Intercept)'          0.068747      0.060551      1.1354    46     0.2621      -0.053135
    'measurement'        -0.0055445       0.01482    -0.37412    46    0.71003      -0.035375
    'drug'                 0.036903      0.079216     0.46585    46    0.64352       -0.12255
```

```
    'ketLvl'             -1.8807e-06    3.8111e-06    -0.49349    46    0.62402    -9.5521e-06
    'sex'                  0.025321      0.033585     0.75391    46    0.45474      -0.042283
    'age'                 -0.019724      0.022571    -0.87388    46    0.38672      -0.065158
    'drugOrder'            0.0027014     0.032389    0.083404    46    0.93389      -0.062495
    'fiveDASC'            -0.0021588    0.0099881    -0.21613    46    0.82984      -0.022264


    Upper
       0.19063
      0.024286
       0.19636
    5.7906e-06
      0.092925
      0.025709
      0.067898
      0.017946

Random effects covariance parameters (95% CIs):
Group: subject (27 Levels)
    Name1                Name2                Type         Estimate    Lower       Upper
    '(Intercept)'        '(Intercept)'        'std'        0.073334    0.049878    0.10782

Group: Error
    Name             Estimate    Lower       Upper
    'Res Std'        0.050392    0.037417    0.067868


 Contrast: cue win vs cue neutral        ROI: striatum 5D-ASC subscore: sum

lme =


Linear mixed-effects model fit by REML

Model information:
    Number of observations             54
    Fixed effects coefficients          8
    Random effects coefficients        27
    Covariance parameters               2

Formula:
    ROI ~ 1 + measurement + drug + ketLvl + sex + age + drugOrder + fiveDASC + (1 | subject)

Model fit statistics:
    AIC        BIC        LogLikelihood    Deviance
    -47.257    -28.971    33.629           -67.257

Fixed effects coefficients (95% CIs):
    Name                 Estimate       SE            tStat       DF    pValue     Lower
    '(Intercept)'           0.071955      0.060116      1.1969    46    0.23746      -0.049052
    'measurement'          -0.009666      0.014747    -0.65546    46    0.51544       -0.03935
    'drug'                  0.031432      0.078256     0.40165    46     0.6898       -0.12609
    'ketLvl'             -1.6212e-06    3.7633e-06    -0.43079    46    0.66863    -9.1962e-06
    'sex'                   0.026045      0.033308     0.78195    46    0.43825         -0.041
    'age'                  -0.018431      0.022414     -0.8223    46    0.41515      -0.063548
    'drugOrder'               0.0047      0.032014     0.14681    46    0.88392       -0.05974
    'fiveDASC'            -0.0093513     0.0097824    -0.95593    46     0.3441      -0.029042


    Upper
       0.19296
      0.020018
       0.18895
    5.9539e-06
       0.09309
      0.026686
       0.06914
       0.01034

Random effects covariance parameters (95% CIs):
Group: subject (27 Levels)
    Name1                Name2                Type         Estimate    Lower       Upper
    '(Intercept)'        '(Intercept)'        'std'        0.072744    0.049708    0.10646

Group: Error
    Name             Estimate    Lower      Upper
    'Res Std'        0.049884    0.03712    0.067036
```

Contrast: cue win vs cue neutral   ROI: nucleus accumbens 5D-ASC subscore: asc_obn

```
lme = 


Linear mixed-effects model fit by REML

Model information:
    Number of observations              54
    Fixed effects coefficients           8
    Random effects coefficients         27
    Covariance parameters                2

Formula:
    ROI ~ 1 + measurement + drug + ketLvl + sex + age + drugOrder + fiveDASC + (1 | subject)

Model fit statistics:
    AIC        BIC       LogLikelihood    Deviance
    -13.467    4.8196    16.733           -33.467

Fixed effects coefficients (95% CIs):
    Name                   Estimate       SE            tStat       DF    pValue      Lower     
    '(Intercept)'              0.13012      0.072523      1.7942    46    0.079354      -0.01586
    'measurement'            -0.018012      0.027596    -0.65271    46     0.51719     -0.073561
    'drug'                     0.17235       0.13477      1.2788    46     0.20737     -0.098932
    'ketLvl'               -8.9361e-06    6.4504e-06     -1.3854    46     0.17262     -2.192e-05
    'sex'                    -0.052071      0.035656     -1.4604    46     0.15098      -0.12384
    'age'                    -0.032211      0.023508     -1.3702    46     0.17726     -0.079529
    'drugOrder'               0.072852      0.033369      2.1832    46    0.034157     0.0056844
    'fiveDASC'              -0.0030862      0.015215    -0.20284    46     0.84015     -0.033711


    Upper     
        0.2761
      0.037536
       0.44363
    4.0478e-06
        0.0197
      0.015107
       0.14002
      0.027539

Random effects covariance parameters (95% CIs):
Group: subject (27 Levels)
    Name1                Name2                Type         Estimate    Lower       Upper  
    '(Intercept)'        '(Intercept)'        'std'        0.048037    0.016413    0.14059

Group: Error
    Name             Estimate    Lower       Upper  
    'Res Std'        0.098057    0.073856    0.13019
```

Contrast: cue win vs cue neutral   ROI: nucleus accumbens 5D-ASC subscore: asc_aed

```
lme = 


Linear mixed-effects model fit by REML

Model information:
    Number of observations              54
    Fixed effects coefficients           8
    Random effects coefficients         27
    Covariance parameters                2

Formula:
    ROI ~ 1 + measurement + drug + ketLvl + sex + age + drugOrder + fiveDASC + (1 | subject)

Model fit statistics:
    AIC        BIC       LogLikelihood    Deviance
    -13.783    4.5034    16.892           -33.783

Fixed effects coefficients (95% CIs):
    Name                   Estimate       SE            tStat       DF    pValue      Lower      
    '(Intercept)'              0.12917      0.072345      1.7855    46     0.08078      -0.016453
    'measurement'            -0.019729      0.026989      -0.731    46     0.46849      -0.074054
    'drug'                     0.16606       0.13463      1.2335    46     0.22367       -0.10493
```

```
    'ketLvl'            -8.6322e-06    6.4464e-06     -1.3391    46     0.18713    -2.1608e-05
    'sex'                 -0.052656       0.03587      -1.468    46     0.14891       -0.12486
    'age'                 -0.030997      0.023742     -1.3056    46     0.19819      -0.078787
    'drugOrder'            0.075608      0.034008      2.2232    46    0.031153      0.0071532
    'fiveDASC'           -0.0088781      0.015403    -0.57638    46     0.56717      -0.039883


    Upper
       0.27479
      0.034597
       0.43706
    4.3437e-06
      0.019545
      0.016793
       0.14406
      0.022127

Random effects covariance parameters (95% CIs):
Group: subject (27 Levels)
    Name1                Name2                Type         Estimate    Lower       Upper
    '(Intercept)'        '(Intercept)'        'std'        0.05043     0.018762    0.13555

Group: Error
    Name             Estimate    Lower      Upper
    'Res Std'        0.096773    0.07277    0.12869


 Contrast: cue win vs cue neutral        ROI: nucleus accumbens 5D-ASC subscore: asc_vis

lme =


Linear mixed-effects model fit by REML

Model information:
    Number of observations              54
    Fixed effects coefficients           8
    Random effects coefficients         27
    Covariance parameters                2

Formula:
    ROI ~ 1 + measurement + drug + ketLvl + sex + age + drugOrder + fiveDASC + (1 | subject)

Model fit statistics:
    AIC        BIC     LogLikelihood    Deviance
    -13.636    4.65    16.818           -33.636

Fixed effects coefficients (95% CIs):
    Name                 Estimate       SE            tStat       DF    pValue      Lower
    '(Intercept)'             0.126      0.072527      1.7373    46    0.089022      -0.019986
    'measurement'         -0.012708      0.028622    -0.44401    46     0.65912      -0.070321
    'drug'                  0.17564       0.13415      1.3093    46     0.19692      -0.094381
    'ketLvl'            -9.0871e-06    6.4197e-06     -1.4155    46     0.16365    -2.2009e-05
    'sex'                 -0.053147      0.035543     -1.4953    46     0.14167       -0.12469
    'age'                 -0.033601      0.023396     -1.4362    46     0.15772      -0.080695
    'drugOrder'            0.070358      0.033489      2.1009    46    0.041155      0.0029485
    'fiveDASC'            0.0062986      0.015574     0.40443    46     0.68777       -0.02505


    Upper
      0.27199
     0.044904
      0.44567
    3.835e-06
     0.018397
     0.013493
      0.13777
     0.037648

Random effects covariance parameters (95% CIs):
Group: subject (27 Levels)
    Name1                Name2                Type         Estimate    Lower       Upper
    '(Intercept)'        '(Intercept)'        'std'        0.047354    0.015742    0.14245

Group: Error
    Name             Estimate    Lower       Upper
    'Res Std'        0.098162    0.073907    0.13038
```

Contrast: cue win vs cue neutral   ROI: nucleus accumbens 5D-ASC subscore: asc_aud

lme =

Linear mixed-effects model fit by REML

Model information:

Number of observations	54
Fixed effects coefficients	8
Random effects coefficients	27
Covariance parameters	2

Formula:
ROI ~ 1 + measurement + drug + ketLvl + sex + age + drugOrder + fiveDASC + (1 | subject)

Model fit statistics:

AIC	BIC	LogLikelihood	Deviance
-13.788	4.498	16.894	-33.788

Fixed effects coefficients (95% CIs):

Name	Estimate	SE	tStat	DF	pValue	Lower	Upper
'(Intercept)'	0.12347	0.072507	1.7029	46	0.095339	-0.022478	0.26942
'measurement'	-0.013157	0.027499	-0.47845	46	0.6346	-0.068508	0.042195
'drug'	0.18429	0.13501	1.365	46	0.17889	-0.087469	0.45604
'ketLvl'	-9.5098e-06	6.4614e-06	-1.4718	46	0.14789	-2.2516e-05	3.4964e-06
'sex'	-0.052238	0.035337	-1.4783	46	0.14615	-0.12337	0.018891
'age'	-0.03173	0.023237	-1.3655	46	0.17874	-0.078505	0.015044
'drugOrder'	0.072485	0.032978	2.198	46	0.033021	0.0061041	0.13887
'fiveDASC'	0.0091988	0.015174	0.60622	46	0.54735	-0.021345	0.039743

Random effects covariance parameters (95% CIs):
Group: subject (27 Levels)

Name1	Name2	Type	Estimate	Lower	Upper
'(Intercept)'	'(Intercept)'	'std'	0.046604	0.014957	0.14521

Group: Error

Name	Estimate	Lower	Upper
'Res Std'	0.09819	0.073928	0.13041

Contrast: cue win vs cue neutral   ROI: nucleus accumbens 5D-ASC subscore: asc_vig

lme =

Linear mixed-effects model fit by REML

Model information:

Number of observations	54
Fixed effects coefficients	8
Random effects coefficients	27
Covariance parameters	2

Formula:
ROI ~ 1 + measurement + drug + ketLvl + sex + age + drugOrder + fiveDASC + (1 | subject)

Model fit statistics:

AIC	BIC	LogLikelihood	Deviance
-13.866	4.4208	16.933	-33.866

Fixed effects coefficients (95% CIs):

Name	Estimate	SE	tStat	DF	pValue	Lower
'(Intercept)'	0.1308	0.072156	1.8128	46	0.076393	-0.014439
'measurement'	-0.021782	0.027917	-0.78024	46	0.43924	-0.077976
'drug'	0.17643	0.13361	1.3205	46	0.19321	-0.092516

```
    'ketLvl'             -9.142e-06    6.3954e-06     -1.4295    46     0.15963    -2.2015e-05
    'sex'                 -0.052233      0.035569     -1.4685    46     0.14877       -0.12383
    'age'                 -0.033113       0.02336     -1.4175    46     0.16307      -0.080134
    'drugOrder'            0.076186      0.033807      2.2535    46    0.029035      0.0081355
    'fiveDASC'           -0.0096355      0.015623    -0.61675    46     0.54044      -0.041083


    Upper
       0.27605
      0.034412
       0.44538
    3.7314e-06
      0.019363
      0.013908
       0.14424
      0.021812

Random effects covariance parameters (95% CIs):
Group: subject (27 Levels)
    Name1                Name2                Type         Estimate    Lower       Upper
    '(Intercept)'        '(Intercept)'        'std'        0.048417    0.016869    0.13897

Group: Error
    Name             Estimate    Lower       Upper
    'Res Std'        0.097485    0.073445    0.12939


 Contrast: cue win vs cue neutral        ROI: nucleus accumbens 5D-ASC subscore: sum

lme =


Linear mixed-effects model fit by REML

Model information:
    Number of observations              54
    Fixed effects coefficients           8
    Random effects coefficients         27
    Covariance parameters                2

Formula:
    ROI ~ 1 + measurement + drug + ketLvl + sex + age + drugOrder + fiveDASC + (1 | subject)

Model fit statistics:
    AIC        BIC       LogLikelihood    Deviance
    -13.545    4.7416    16.772           -33.545

Fixed effects coefficients (95% CIs):
    Name                 Estimate       SE            tStat       DF    pValue      Lower
    '(Intercept)'            0.1303      0.072627      1.7941    46    0.079373      -0.015891
    'measurement'         -0.018616       0.02817    -0.66083    46     0.51202       -0.07532
    'drug'                  0.17277       0.13462      1.2834    46     0.20579      -0.098213
    'ketLvl'            -8.9577e-06    6.4434e-06     -1.3902    46     0.17115    -2.1927e-05
    'sex'                 -0.052119      0.035731     -1.4587    46     0.15145       -0.12404
    'age'                  -0.03227      0.023533     -1.3713    46     0.17695      -0.079639
    'drugOrder'             0.07336      0.033712      2.1761    46    0.034724      0.0055005
    'fiveDASC'           -0.0034002      0.015771     -0.2156    46     0.83025      -0.035145


    Upper
       0.27649
      0.038088
       0.44376
    4.0121e-06
      0.019804
        0.0151
       0.14122
      0.028345

Random effects covariance parameters (95% CIs):
Group: subject (27 Levels)
    Name1                Name2                Type         Estimate    Lower       Upper
    '(Intercept)'        '(Intercept)'        'std'        0.048744    0.017128    0.13871

Group: Error
    Name             Estimate    Lower       Upper
    'Res Std'        0.09778     0.073678    0.12977
```

```
 Contrast: cue win vs cue neutral       ROI: insula 5D-ASC subscore: asc_obn

lme = 


Linear mixed-effects model fit by REML

Model information:
    Number of observations              54
    Fixed effects coefficients           8
    Random effects coefficients         27
    Covariance parameters                2

Formula:
    ROI ~ 1 + measurement + drug + ketLvl + sex + age + drugOrder + fiveDASC + (1 | subject)

Model fit statistics:
    AIC       BIC       LogLikelihood    Deviance
    28.815    47.101    -4.4074          8.8147  

Fixed effects coefficients (95% CIs):
    Name                    Estimate       SE            tStat       DF    pValue     Lower      
    '(Intercept)'             0.054244       0.13136     0.41293    46    0.68158       -0.21018
    'measurement'             0.015944      0.034363       0.464    46    0.64484      -0.053224
    'drug'                    0.083044       0.18682     0.44452    46    0.65875         -0.293
    'ketLvl'               -3.3362e-06    8.9797e-06    -0.37153    46    0.71195    -2.1411e-05
    'sex'                     0.031098      0.071792     0.43317    46    0.66692       -0.11341
    'age'                    -0.038103      0.048192    -0.79065    46     0.4332       -0.13511
    'drugOrder'              -0.011262      0.068553    -0.16427    46    0.87023       -0.14925
    'fiveDASC'              0.00058218      0.021611    0.026939    46    0.97862      -0.042919


    Upper     
       0.31867
      0.085113
       0.45909
    1.4739e-05
       0.17561
      0.058902
       0.12673
      0.044083

Random effects covariance parameters (95% CIs):
Group: subject (27 Levels)
    Name1                Name2                Type         Estimate    Lower      Upper 
    '(Intercept)'        '(Intercept)'        'std'        0.15114     0.10139    0.2253

Group: Error
    Name             Estimate    Lower       Upper  
    'Res Std'        0.12083     0.090506    0.16131


 Contrast: cue win vs cue neutral       ROI: insula 5D-ASC subscore: asc_aed

lme = 


Linear mixed-effects model fit by REML

Model information:
    Number of observations              54
    Fixed effects coefficients           8
    Random effects coefficients         27
    Covariance parameters                2

Formula:
    ROI ~ 1 + measurement + drug + ketLvl + sex + age + drugOrder + fiveDASC + (1 | subject)

Model fit statistics:
    AIC       BIC       LogLikelihood    Deviance
    28.748    47.034    -4.3739          8.7479  

Fixed effects coefficients (95% CIs):
    Name                    Estimate       SE            tStat       DF    pValue     Lower      
    '(Intercept)'             0.054397       0.13111      0.4149    46    0.68014       -0.20951
    'measurement'              0.01668      0.033964      0.4911    46    0.62569      -0.051686
    'drug'                    0.085248       0.18719     0.45541    46    0.65096       -0.29155
```

```
    'ketLvl'              -3.4424e-06    8.9982e-06    -0.38257    46      0.7038    -2.1555e-05
    'sex'                    0.031233      0.071751      0.4353    46     0.66538       -0.11319
    'age'                   -0.038565      0.048253    -0.79922    46     0.42827       -0.13569
    'drugOrder'             -0.012225      0.068936    -0.17734    46     0.86002       -0.15099
    'fiveDASC'              0.0028744      0.022162      0.1297    46     0.89737      -0.041734


    Upper
      0.31831
     0.085046
      0.46204
    1.467e-05
      0.17566
     0.058564
      0.12653
     0.047483

Random effects covariance parameters (95% CIs):
Group: subject (27 Levels)
    Name1                 Name2                 Type         Estimate    Lower      Upper
    '(Intercept)'         '(Intercept)'         'std'        0.15113     0.10143    0.22519

Group: Error
    Name             Estimate    Lower       Upper
    'Res Std'        0.1208      0.090512    0.16121


 Contrast: cue win vs cue neutral        ROI: insula 5D-ASC subscore: asc_vis

lme =


Linear mixed-effects model fit by REML

Model information:
    Number of observations              54
    Fixed effects coefficients           8
    Random effects coefficients         27
    Covariance parameters                2

Formula:
    ROI ~ 1 + measurement + drug + ketLvl + sex + age + drugOrder + fiveDASC + (1 | subject)

Model fit statistics:
    AIC       BIC       LogLikelihood    Deviance
    28.704    46.991    -4.3522          8.7045

Fixed effects coefficients (95% CIs):
    Name                  Estimate       SE            tStat       DF    pValue     Lower
    '(Intercept)'            0.057557       0.13101     0.43934    46    0.66248       -0.20615
    'measurement'            0.011558      0.036025     0.32083    46     0.7498      -0.060957
    'drug'                   0.080229       0.18657     0.43003    46    0.66918       -0.29531
    'ketLvl'              -3.2067e-06    8.9675e-06     -0.3576    46    0.72228    -2.1257e-05
    'sex'                    0.031842      0.071439     0.44573    46    0.65788       -0.11196
    'age'                   -0.037093      0.047909    -0.77425    46    0.44275       -0.13353
    'drugOrder'            -0.0091073       0.06842    -0.13311    46    0.89469       -0.14683
    'fiveDASC'             -0.0065685      0.021897    -0.29998    46    0.76554      -0.050644


    Upper
       0.32127
      0.084073
       0.45577
    1.4844e-05
       0.17564
      0.059342
       0.12861
      0.037507

Random effects covariance parameters (95% CIs):
Group: subject (27 Levels)
    Name1                 Name2                 Type         Estimate    Lower       Upper
    '(Intercept)'         '(Intercept)'         'std'        0.14989     0.099984    0.22471

Group: Error
    Name             Estimate    Lower       Upper
    'Res Std'        0.12124     0.090671    0.16212
```

```
 Contrast: cue win vs cue neutral       ROI: insula 5D-ASC subscore: asc_aud

lme =


Linear mixed-effects model fit by REML

Model information:
    Number of observations              54
    Fixed effects coefficients           8
    Random effects coefficients         27
    Covariance parameters                2

Formula:
    ROI ~ 1 + measurement + drug + ketLvl + sex + age + drugOrder + fiveDASC + (1 | subject)

Model fit statistics:
    AIC       BIC      LogLikelihood    Deviance
    27.684    45.97    -3.8419          7.6839

Fixed effects coefficients (95% CIs):
    Name                 Estimate       SE            tStat       DF    pValue     Lower
    '(Intercept)'           0.068214       0.12986      0.5253    46     0.6019       -0.19318
    'measurement'          0.0069484      0.033976     0.20451    46    0.83886      -0.061441
    'drug'                  0.052076       0.18672     0.27891    46    0.78157       -0.32377
    'ketLvl'             -1.8412e-06    8.9756e-06    -0.20513    46    0.83837    -1.9908e-05
    'sex'                   0.030284      0.070601     0.42894    46    0.66997       -0.11183
    'age'                   -0.04053      0.047338    -0.85618    46    0.39634       -0.13582
    'drugOrder'            -0.011534      0.067326    -0.17131    46    0.86473       -0.14705
    'fiveDASC'             -0.023024      0.022227     -1.0359    46    0.30569      -0.067764


    Upper
        0.3296
      0.075338
       0.42792
    1.6226e-05
        0.1724
      0.054757
       0.12399
      0.021717

Random effects covariance parameters (95% CIs):
Group: subject (27 Levels)
    Name1                Name2                Type         Estimate    Lower       Upper
    '(Intercept)'        '(Intercept)'        'std'        0.14817     0.098936    0.2219

Group: Error
    Name             Estimate    Lower       Upper
    'Res Std'        0.12004     0.089902    0.16028


 Contrast: cue win vs cue neutral       ROI: insula 5D-ASC subscore: asc_vig

lme =


Linear mixed-effects model fit by REML

Model information:
    Number of observations              54
    Fixed effects coefficients           8
    Random effects coefficients         27
    Covariance parameters                2

Formula:
    ROI ~ 1 + measurement + drug + ketLvl + sex + age + drugOrder + fiveDASC + (1 | subject)

Model fit statistics:
    AIC       BIC       LogLikelihood    Deviance
    28.566    46.852    -4.283           8.566

Fixed effects coefficients (95% CIs):
    Name                 Estimate       SE            tStat       DF    pValue     Lower
    '(Intercept)'           0.053054       0.13118     0.40442    46    0.68778       -0.21101
    'measurement'           0.019617      0.035306     0.55563    46    0.58116       -0.05145
    'drug'                  0.078658       0.18635      0.4221    46    0.67492       -0.29644
```

```
    'ketLvl'            -3.1155e-06    8.9602e-06     -0.3477    46    0.72965    -2.1151e-05
    'sex'                   0.03084      0.071783     0.42963    46    0.66947       -0.11365
    'age'                 -0.037731       0.04809    -0.78459    46    0.43672       -0.13453
    'drugOrder'           -0.014088      0.069098    -0.20389    46    0.83934       -0.15318
    'fiveDASC'            0.0073372      0.023251     0.31557    46    0.75376      -0.039464


    Upper
      0.31711
     0.090684
      0.45375
    1.492e-05
      0.17533
     0.059069
        0.125
     0.054139

Random effects covariance parameters (95% CIs):
Group: subject (27 Levels)
    Name1                Name2                Type         Estimate    Lower      Upper
    '(Intercept)'        '(Intercept)'        'std'        0.15132     0.10166    0.22525

Group: Error
    Name             Estimate    Lower       Upper
    'Res Std'        0.12053     0.090377    0.16075


 Contrast: cue win vs cue neutral        ROI: insula 5D-ASC subscore: sum

lme =


Linear mixed-effects model fit by REML

Model information:
    Number of observations             54
    Fixed effects coefficients          8
    Random effects coefficients        27
    Covariance parameters               2

Formula:
    ROI ~ 1 + measurement + drug + ketLvl + sex + age + drugOrder + fiveDASC + (1 | subject)

Model fit statistics:
    AIC       BIC       LogLikelihood    Deviance
    28.664    46.951    -4.3321          8.6642

Fixed effects coefficients (95% CIs):
    Name                 Estimate       SE            tStat       DF    pValue     Lower
    '(Intercept)'           0.053267       0.13158     0.40483    46    0.68748       -0.21159
    'measurement'           0.017425      0.035511     0.49069    46    0.62598      -0.054056
    'drug'                  0.084925       0.18624       0.456    46    0.65053       -0.28995
    'ketLvl'            -3.4253e-06    8.9515e-06    -0.38265    46    0.70374    -2.1444e-05
    'sex'                   0.030955      0.071902     0.43052    46    0.66883       -0.11378
    'age'                    -0.0384      0.048245    -0.79594    46    0.43015       -0.13551
    'drugOrder'            -0.012113      0.068937    -0.17572    46    0.86129       -0.15088
    'fiveDASC'             0.0030944      0.023073     0.13411    46     0.8939      -0.043349


    Upper
       0.31812
      0.088906
        0.4598
    1.4593e-05
       0.17569
      0.058711
       0.12665
      0.049538

Random effects covariance parameters (95% CIs):
Group: subject (27 Levels)
    Name1                Name2                Type         Estimate    Lower     Upper
    '(Intercept)'        '(Intercept)'        'std'        0.1516      0.1018    0.22576

Group: Error
    Name             Estimate    Lower      Upper
    'Res Std'        0.12057     0.09035    0.16091
```

Contrast: cue win vs cue neutral ROI: thalamus 5D-ASC subscore: asc_obn

lme =

Linear mixed-effects model fit by REML

Model information:

Number of observations	54
Fixed effects coefficients	8
Random effects coefficients	27
Covariance parameters	2

Formula:

ROI ~ 1 + measurement + drug + ketLvl + sex + age + drugOrder + fiveDASC + (1 | subject)

Model fit statistics:

AIC	BIC	LogLikelihood	Deviance
-26.91	-8.6233	23.455	-46.91

Fixed effects coefficients (95% CIs):

Name	Estimate	SE	tStat	DF	pValue	Lower
'(Intercept)'	0.08046	0.064886	1.24	46	0.22126	-0.050149
'measurement'	-0.0074636	0.02201	-0.3391	46	0.73608	-0.051768
'drug'	0.051608	0.11255	0.45854	46	0.64872	-0.17494
'ketLvl'	-1.4899e-06	5.3972e-06	-0.27605	46	0.78374	-1.2354e-05
'sex'	0.019595	0.033389	0.58688	46	0.56015	-0.047613
'age'	-0.013747	0.022168	-0.62011	46	0.53825	-0.05837
'drugOrder'	0.019358	0.031494	0.61467	46	0.5418	-0.044035
'fiveDASC'	-0.0020405	0.012831	-0.15903	46	0.87435	-0.027868

Upper
0.21107
0.036841
0.27816
9.3741e-06
0.086804
0.030876
0.082751
0.023787

Random effects covariance parameters (95% CIs):

Group: subject (27 Levels)

Name1	Name2	Type	Estimate	Lower	Upper
'(Intercept)'	'(Intercept)'	'std'	0.057564	0.030729	0.10783

Group: Error

Name	Estimate	Lower	Upper
'Res Std'	0.07789	0.058412	0.10386

Contrast: cue win vs cue neutral ROI: thalamus 5D-ASC subscore: asc_aed

lme =

Linear mixed-effects model fit by REML

Model information:

Number of observations	54
Fixed effects coefficients	8
Random effects coefficients	27
Covariance parameters	2

Formula:

ROI ~ 1 + measurement + drug + ketLvl + sex + age + drugOrder + fiveDASC + (1 | subject)

Model fit statistics:

AIC	BIC	LogLikelihood	Deviance
-27.24	-8.9537	23.62	-47.24

Fixed effects coefficients (95% CIs):

Name	Estimate	SE	tStat	DF	pValue	Lower
'(Intercept)'	0.079556	0.064432	1.2347	46	0.2232	-0.050139
'measurement'	-0.0041035	0.021768	-0.18851	46	0.85131	-0.04792
'drug'	0.060644	0.1128	0.53761	46	0.59344	-0.16641

```
    'ketLvl'            -1.9248e-06    5.4097e-06     -0.3558    46    0.72362    -1.2814e-05
    'sex'                  0.019631      0.033183     0.59159    46    0.55702      -0.047163
    'age'                 -0.015485      0.022087    -0.70106    46    0.48679      -0.059944
    'drugOrder'            0.016278      0.031609     0.51498    46    0.60903      -0.047347
    'fiveDASC'            0.0073484      0.013062     0.56257    46    0.57646      -0.018944


    Upper
       0.20925
      0.039713
        0.2877
    8.9645e-06
      0.086425
      0.028975
      0.079902
      0.033641

Random effects covariance parameters (95% CIs):
Group: subject (27 Levels)
    Name1                Name2                Type         Estimate    Lower       Upper
    '(Intercept)'        '(Intercept)'        'std'        0.056961    0.030272    0.10718

Group: Error
    Name             Estimate    Lower       Upper
    'Res Std'        0.077839    0.058425    0.10371


 Contrast: cue win vs cue neutral        ROI: thalamus 5D-ASC subscore: asc_vis

lme =


Linear mixed-effects model fit by REML

Model information:
    Number of observations              54
    Fixed effects coefficients           8
    Random effects coefficients         27
    Covariance parameters                2

Formula:
    ROI ~ 1 + measurement + drug + ketLvl + sex + age + drugOrder + fiveDASC + (1 | subject)

Model fit statistics:
    AIC        BIC        LogLikelihood    Deviance
    -27.081    -8.7951    23.541           -47.081

Fixed effects coefficients (95% CIs):
    Name                 Estimate      SE            tStat       DF    pValue     Lower
    '(Intercept)'          0.082181      0.064959      1.2651    46     0.2122      -0.048575
    'measurement'        -0.0099199      0.022824    -0.43463    46    0.66587      -0.055862
    'drug'                  0.05279       0.11188     0.47185    46    0.63926       -0.17241
    'ketLvl'             -1.552e-06    5.3651e-06    -0.28928    46    0.77367    -1.2351e-05
    'sex'                  0.020043      0.033397     0.60015    46    0.55135      -0.047182
    'age'                 -0.013327      0.022152    -0.60164    46    0.55037      -0.057916
    'drugOrder'            0.020625      0.031677     0.65111    46    0.51822      -0.043138
    'fiveDASC'           -0.0052199      0.013051    -0.39998    46    0.69103       -0.03149


    Upper
       0.21294
      0.036022
       0.27799
    9.2474e-06
      0.087269
      0.031262
      0.084388
       0.02105

Random effects covariance parameters (95% CIs):
Group: subject (27 Levels)
    Name1                Name2                Type         Estimate    Lower       Upper
    '(Intercept)'        '(Intercept)'        'std'        0.05772     0.030953    0.10763

Group: Error
    Name             Estimate    Lower       Upper
    'Res Std'        0.077666    0.058238    0.10358
```

```
 Contrast: cue win vs cue neutral       ROI: thalamus 5D-ASC subscore: asc_aud

lme = 


Linear mixed-effects model fit by REML

Model information:
    Number of observations              54
    Fixed effects coefficients           8
    Random effects coefficients         27
    Covariance parameters                2

Formula:
    ROI ~ 1 + measurement + drug + ketLvl + sex + age + drugOrder + fiveDASC + (1 | subject)

Model fit statistics:
    AIC        BIC        LogLikelihood    Deviance
    -26.958    -8.6713    23.479           -46.958 

Fixed effects coefficients (95% CIs):
    Name                 Estimate       SE            tStat       DF    pValue     Lower      
    '(Intercept)'           0.081357      0.065223      1.2474    46    0.21858        -0.04993
    'measurement'          -0.007663      0.021864    -0.35048    46    0.72758       -0.051673
    'drug'                  0.050449       0.11302     0.44638    46    0.65742        -0.17704
    'ketLvl'             -1.4337e-06    5.4205e-06     -0.2645    46    0.79258     -1.2345e-05
    'sex'                   0.019351      0.033415     0.57912    46    0.56534       -0.047909
    'age'                  -0.014364      0.022154    -0.64837    46    0.51997       -0.058956
    'drugOrder'             0.018953      0.031467     0.60232    46    0.54992       -0.044387
    'fiveDASC'            -0.0027492       0.01301    -0.21131    46    0.83358       -0.028938


    Upper     
       0.21264
      0.036348
       0.27794
    9.4773e-06
      0.086612
      0.030229
      0.082293
      0.023439

Random effects covariance parameters (95% CIs):
Group: subject (27 Levels)
    Name1                Name2                Type         Estimate    Lower       Upper  
    '(Intercept)'        '(Intercept)'        'std'        0.057848    0.031063    0.10773

Group: Error
    Name             Estimate    Lower       Upper  
    'Res Std'        0.077738    0.058349    0.10357


 Contrast: cue win vs cue neutral       ROI: thalamus 5D-ASC subscore: asc_vig

lme = 


Linear mixed-effects model fit by REML

Model information:
    Number of observations              54
    Fixed effects coefficients           8
    Random effects coefficients         27
    Covariance parameters                2

Formula:
    ROI ~ 1 + measurement + drug + ketLvl + sex + age + drugOrder + fiveDASC + (1 | subject)

Model fit statistics:
    AIC       BIC        LogLikelihood    Deviance
    -27.01    -8.7234    23.505           -47.01  

Fixed effects coefficients (95% CIs):
    Name                 Estimate       SE            tStat       DF    pValue     Lower      
    '(Intercept)'            0.07925      0.064757      1.2238    46    0.22726         -0.0511
    'measurement'         -0.0053217      0.022486    -0.23667    46    0.81397       -0.050584
    'drug'                  0.052442       0.11217     0.46752    46    0.64234        -0.17335
```

```
    'ketLvl'            -1.5267e-06    5.3798e-06    -0.28379    46    0.77784    -1.2356e-05
    'sex'                  0.019324      0.033361     0.57924    46    0.56525      -0.047828
    'age'                 -0.013975      0.022077    -0.63303    46    0.52985      -0.058414
    'drugOrder'            0.018037      0.031869     0.56598    46    0.57416      -0.046111
    'fiveDASC'             0.002414      0.013442     0.17959    46    0.85826      -0.024643


    Upper
       0.2096
      0.03994
      0.27823
    9.3023e-06
     0.086476
     0.030463
     0.082185
     0.029471

Random effects covariance parameters (95% CIs):
Group: subject (27 Levels)
    Name1                Name2                Type         Estimate    Lower       Upper
    '(Intercept)'        '(Intercept)'        'std'        0.057549    0.030781    0.1076

Group: Error
    Name             Estimate    Lower       Upper
    'Res Std'        0.077889    0.058489    0.10372


 Contrast: cue win vs cue neutral        ROI: thalamus 5D-ASC subscore: sum

lme =


Linear mixed-effects model fit by REML

Model information:
    Number of observations              54
    Fixed effects coefficients           8
    Random effects coefficients         27
    Covariance parameters                2

Formula:
    ROI ~ 1 + measurement + drug + ketLvl + sex + age + drugOrder + fiveDASC + (1 | subject)

Model fit statistics:
    AIC        BIC        LogLikelihood    Deviance
    -26.984    -8.6978    23.492           -46.984

Fixed effects coefficients (95% CIs):
    Name                Estimate       SE            tStat       DF    pValue     Lower
    '(Intercept)'          0.079355      0.064912      1.2225    46    0.22775      -0.051307
    'measurement'        -0.0060858      0.022642    -0.26879    46    0.78929      -0.051661
    'drug'                 0.053617       0.11253     0.47647    46    0.63599       -0.17289
    'ketLvl'            -1.5855e-06    5.3958e-06    -0.29383    46    0.77021    -1.2447e-05
    'sex'                  0.019308      0.033361     0.57876    46    0.56558      -0.047844
    'age'                 -0.014192      0.022127    -0.64138    46    0.52446      -0.058731
    'drugOrder'            0.018725      0.031668     0.59129    46    0.55722       -0.04502
    'fiveDASC'           0.00091167      0.013462    0.067722    46     0.9463      -0.026186


    Upper
      0.21002
      0.03949
      0.28012
    9.2757e-06
      0.08646
     0.030347
      0.08247
     0.028009

Random effects covariance parameters (95% CIs):
Group: subject (27 Levels)
    Name1                Name2                Type         Estimate    Lower      Upper
    '(Intercept)'        '(Intercept)'        'std'        0.057449    0.03059    0.10789

Group: Error
    Name             Estimate    Lower       Upper
    'Res Std'        0.077966    0.058496    0.10392
```

```
 Contrast: cue win vs cue neutral        ROI: amygdala 5D-ASC subscore: asc_obn

lme = 


Linear mixed-effects model fit by REML

Model information:
    Number of observations              54
    Fixed effects coefficients           8
    Random effects coefficients         27
    Covariance parameters                2

Formula:
    ROI ~ 1 + measurement + drug + ketLvl + sex + age + drugOrder + fiveDASC + (1 | subject)

Model fit statistics:
    AIC       BIC       LogLikelihood    Deviance
    25.013    43.299    -2.5064          5.0128  

Fixed effects coefficients (95% CIs):
    Name                    Estimate      SE           tStat       DF    pValue      Lower      
    '(Intercept)'              0.20175      0.12326      1.6367    46      0.10852      -0.046371
    'measurement'            -0.018042     0.034034    -0.53011    46      0.59858       -0.08655
    'drug'                     0.22363      0.18329      1.2201    46      0.22864       -0.14531
    'ketLvl'                -1.363e-05    8.807e-06     -1.5476    46      0.12858    -3.1357e-05
    'sex'                     0.060634     0.066744     0.90846    46      0.36837      -0.073714
    'age'                     -0.06199     0.044721     -1.3862    46      0.17238       -0.15201
    'drugOrder'              -0.083976     0.063602     -1.3203    46      0.19325         -0.212
    'fiveDASC'               -0.037525     0.021152     -1.7741    46     0.082672      -0.080103


    Upper    
      0.44986
     0.050466
      0.59257
    4.098e-06
      0.19498
     0.028029
     0.044048
    0.0050518

Random effects covariance parameters (95% CIs):
Group: subject (27 Levels)
    Name1                Name2                Type         Estimate    Lower       Upper  
    '(Intercept)'        '(Intercept)'        'std'        0.13696     0.088796    0.21126

Group: Error
    Name             Estimate    Lower       Upper 
    'Res Std'        0.1198      0.089312    0.1607


 Contrast: cue win vs cue neutral        ROI: amygdala 5D-ASC subscore: asc_aed

lme = 


Linear mixed-effects model fit by REML

Model information:
    Number of observations              54
    Fixed effects coefficients           8
    Random effects coefficients         27
    Covariance parameters                2

Formula:
    ROI ~ 1 + measurement + drug + ketLvl + sex + age + drugOrder + fiveDASC + (1 | subject)

Model fit statistics:
    AIC       BIC       LogLikelihood    Deviance
    27.416    45.702    -3.7079          7.4159  

Fixed effects coefficients (95% CIs):
    Name                    Estimate      SE            tStat       DF    pValue     Lower    
    '(Intercept)'              0.18831       0.12816      1.4693    46     0.14856     -0.069671
    'measurement'           -0.0077954      0.033863     -0.2302    46     0.81896     -0.075958
    'drug'                     0.24451       0.18603      1.3144    46     0.19524      -0.12995
```

```
    'ketLvl'            -1.463e-05    8.9413e-06     -1.6362    46    0.10862    -3.2628e-05
    'sex'                 0.056885      0.069917     0.81361    46    0.42006       -0.08385
    'age'                -0.064809      0.046992     -1.3792    46    0.17452        -0.1594
    'drugOrder'           -0.08457       0.06714     -1.2596    46    0.21417       -0.21972
    'fiveDASC'           -0.016106      0.021996    -0.73221    46    0.46775      -0.060382


    Upper
      0.44628
     0.060368
      0.61897
    3.368e-06
      0.19762
     0.029781
     0.050577
      0.02817

Random effects covariance parameters (95% CIs):
Group: subject (27 Levels)
    Name1               Name2               Type         Estimate    Lower       Upper
    '(Intercept)'       '(Intercept)'       'std'        0.146       0.097103    0.21951

Group: Error
    Name             Estimate    Lower      Upper
    'Res Std'        0.12048     0.09022    0.16088


 Contrast: cue win vs cue neutral        ROI: amygdala 5D-ASC subscore: asc_vis

lme =


Linear mixed-effects model fit by REML

Model information:
    Number of observations              54
    Fixed effects coefficients           8
    Random effects coefficients         27
    Covariance parameters                2

Formula:
    ROI ~ 1 + measurement + drug + ketLvl + sex + age + drugOrder + fiveDASC + (1 | subject)

Model fit statistics:
    AIC       BIC       LogLikelihood    Deviance
    24.798    43.084    -2.3989          4.7979

Fixed effects coefficients (95% CIs):
    Name                Estimate       SE            tStat       DF    pValue      Lower
    '(Intercept)'          0.20597       0.12628        1.631    46     0.10972     -0.048227
    'measurement'        -0.026517       0.03426     -0.77401    46     0.44289     -0.095478
    'drug'                 0.26138       0.17779       1.4702    46     0.14832     -0.096488
    'ketLvl'            -1.5487e-05    8.5463e-06     -1.8122    46    0.076493     -3.269e-05
    'sex'                 0.062449      0.069014      0.90487    46     0.37025     -0.076469
    'age'                -0.062438      0.046304      -1.3484    46     0.18412      -0.15564
    'drugOrder'          -0.078788      0.066124      -1.1915    46     0.23956      -0.21189
    'fiveDASC'           -0.037929      0.020871      -1.8173    46    0.075691     -0.079941


    Upper
       0.46016
      0.042444
       0.61926
    1.7156e-06
       0.20137
      0.030767
      0.054313
     0.0040827

Random effects covariance parameters (95% CIs):
Group: subject (27 Levels)
    Name1               Name2               Type         Estimate    Lower       Upper
    '(Intercept)'       '(Intercept)'       'std'        0.14566     0.097972    0.21656

Group: Error
    Name             Estimate    Lower       Upper
    'Res Std'        0.11525     0.086317    0.15387
```

```
 Contrast: cue win vs cue neutral       ROI: amygdala 5D-ASC subscore: asc_aud

lme =


Linear mixed-effects model fit by REML

Model information:
    Number of observations             54
    Fixed effects coefficients          8
    Random effects coefficients        27
    Covariance parameters               2

Formula:
    ROI ~ 1 + measurement + drug + ketLvl + sex + age + drugOrder + fiveDASC + (1 | subject)

Model fit statistics:
    AIC       BIC       LogLikelihood    Deviance
    27.289    45.575    -3.6444          7.2889

Fixed effects coefficients (95% CIs):
    Name                     Estimate       SE             tStat       DF    pValue     Lower
    '(Intercept)'              0.19942        0.12648       1.5767     46    0.12171      -0.055168
    'measurement'           -0.0094466        0.03492     -0.27052     46    0.78797      -0.079737
    'drug'                     0.22527        0.18999       1.1857     46    0.24183       -0.15716
    'ketLvl'               -1.3694e-05     9.1296e-06      -1.4999     46    0.14047    -3.2071e-05
    'sex'                     0.055928       0.068095      0.82133     46     0.4157       -0.08114
    'age'                    -0.070132       0.045567      -1.5391     46    0.13063       -0.16185
    'drugOrder'              -0.090787       0.064793      -1.4012     46    0.16787       -0.22121
    'fiveDASC'               -0.019278       0.022482     -0.85746     46    0.39563      -0.064532


    Upper
       0.45401
      0.060844
       0.60769
    4.6832e-06
         0.193
       0.02159
      0.039634
      0.025977

Random effects covariance parameters (95% CIs):
Group: subject (27 Levels)
    Name1                Name2                 Type         Estimate    Lower       Upper
    '(Intercept)'        '(Intercept)'         'std'        0.1392      0.088256    0.21954

Group: Error
    Name             Estimate    Lower      Upper
    'Res Std'        0.12352     0.09141    0.1669


 Contrast: cue win vs cue neutral       ROI: amygdala 5D-ASC subscore: asc_vig

lme =


Linear mixed-effects model fit by REML

Model information:
    Number of observations             54
    Fixed effects coefficients          8
    Random effects coefficients        27
    Covariance parameters               2

Formula:
    ROI ~ 1 + measurement + drug + ketLvl + sex + age + drugOrder + fiveDASC + (1 | subject)

Model fit statistics:
    AIC       BIC       LogLikelihood    Deviance
    24.713    42.999    -2.3565          4.713

Fixed effects coefficients (95% CIs):
    Name                     Estimate       SE             tStat       DF    pValue      Lower
    '(Intercept)'              0.19577        0.12352       1.5849     46     0.11985      -0.052872
    'measurement'            -0.024059       0.034691     -0.69354     46     0.49146      -0.093889
    'drug'                     0.27761        0.18186       1.5265     46     0.13373      -0.088454
```

```
    'ketLvl'            -1.6275e-05    8.7418e-06     -1.8617    46    0.069043    -3.3871e-05
    'sex'                  0.058799      0.067099      0.8763    46     0.38542      -0.076265
    'age'                 -0.069517      0.044879      -1.549    46     0.12824       -0.15985
    'drugOrder'           -0.074192      0.064527     -1.1498    46     0.25618       -0.20408
    'fiveDASC'            -0.040891      0.022569     -1.8118    46    0.076541       -0.08632


    Upper
        0.4444
       0.04577
       0.64368
    1.3218e-06
       0.19386
       0.02082
      0.055694
     0.0045374

Random effects covariance parameters (95% CIs):
Group: subject (27 Levels)
    Name1                Name2                Type         Estimate    Lower       Upper
    '(Intercept)'        '(Intercept)'        'std'        0.13864     0.090656    0.21202

Group: Error
    Name             Estimate    Lower       Upper
    'Res Std'        0.11868     0.088631    0.1589


 Contrast: cue win vs cue neutral        ROI: amygdala 5D-ASC subscore: sum

lme =


Linear mixed-effects model fit by REML

Model information:
    Number of observations             54
    Fixed effects coefficients          8
    Random effects coefficients        27
    Covariance parameters               2

Formula:
    ROI ~ 1 + measurement + drug + ketLvl + sex + age + drugOrder + fiveDASC + (1 | subject)

Model fit statistics:
    AIC       BIC       LogLikelihood    Deviance
    24.725    43.012    -2.3626          4.7251

Fixed effects coefficients (95% CIs):
    Name                 Estimate       SE            tStat       DF    pValue     Lower
    '(Intercept)'           0.20377       0.12306      1.6558    46    0.10457     -0.043946
    'measurement'         -0.025203      0.035152    -0.71698    46    0.47701      -0.09596
    'drug'                  0.22925       0.18277      1.2543    46    0.21606      -0.13864
    'ketLvl'            -1.3917e-05    8.7815e-06     -1.5848    46    0.11987    -3.1593e-05
    'sex'                  0.060047       0.06658     0.90188    46    0.37182     -0.073971
    'age'                  -0.06274      0.044582     -1.4073    46    0.16606      -0.15248
    'drugOrder'           -0.077911      0.063722     -1.2227    46    0.22768      -0.20618
    'fiveDASC'            -0.041018      0.022503     -1.8228    46    0.07484     -0.086313


    Upper
       0.45148
      0.045554
       0.59714
    3.7596e-06
       0.19406
      0.026999
      0.050355
     0.0042781

Random effects covariance parameters (95% CIs):
Group: subject (27 Levels)
    Name1                Name2                Type         Estimate    Lower       Upper
    '(Intercept)'        '(Intercept)'        'std'        0.13658     0.088477    0.21085

Group: Error
    Name             Estimate    Lower       Upper
    'Res Std'        0.11966     0.089239    0.16045
```

Contrast: cue loss vs neutral   ROI: striatum 5D-ASC subscore: asc_obn

lme =

Linear mixed-effects model fit by REML

Model information:

Number of observations	54
Fixed effects coefficients	8
Random effects coefficients	27
Covariance parameters	2

Formula:
ROI ~ 1 + measurement + drug + ketLvl + sex + age + drugOrder + fiveDASC + (1 | subject)

Model fit statistics:

AIC	BIC	LogLikelihood	Deviance
-39.485	-21.199	29.743	-59.485

Fixed effects coefficients (95% CIs):

Name	Estimate	SE	tStat	DF	pValue	Lower
'(Intercept)'	0.040655	0.057286	0.70968	46	0.48148	-0.074656
'measurement'	-0.0082372	0.01876	-0.43908	46	0.66266	-0.046
'drug'	-0.018218	0.097002	-0.1878	46	0.85186	-0.21347
'ketLvl'	2.4737e-07	4.6538e-06	0.053155	46	0.95784	-9.1202e-06
'sex'	0.029135	0.029804	0.97757	46	0.3334	-0.030856
'age'	-0.020153	0.019824	-1.0166	46	0.31466	-0.060058
'drugOrder'	0.019707	0.02817	0.69958	46	0.48771	-0.036996
'fiveDASC'	0.00025654	0.011087	0.02314	46	0.98164	-0.02206

Upper
0.15597
0.029525
0.17704
9.6149e-06
0.089126
0.019751
0.076409
0.022573

Random effects covariance parameters (95% CIs):
Group: subject (27 Levels)

Name1	Name2	Type	Estimate	Lower	Upper
'(Intercept)'	'(Intercept)'	'std'	0.053674	0.028915	0.099634

Group: Error

Name	Estimate	Lower	Upper
'Res Std'	0.066318	0.048985	0.089784

Contrast: cue loss vs neutral   ROI: striatum 5D-ASC subscore: asc_aed

lme =

Linear mixed-effects model fit by REML

Model information:

Number of observations	54
Fixed effects coefficients	8
Random effects coefficients	27
Covariance parameters	2

Formula:
ROI ~ 1 + measurement + drug + ketLvl + sex + age + drugOrder + fiveDASC + (1 | subject)

Model fit statistics:

AIC	BIC	LogLikelihood	Deviance
-39.528	-21.241	29.764	-59.528

Fixed effects coefficients (95% CIs):

Name	Estimate	SE	tStat	DF	pValue	Lower
'(Intercept)'	0.040758	0.057148	0.71321	46	0.47932	-0.074274
'measurement'	-0.008479	0.018559	-0.45686	46	0.64992	-0.045837
'drug'	-0.018717	0.097361	-0.19224	46	0.8484	-0.21469

```
    'ketLvl'             2.7129e-07    4.6715e-06     0.058074    46    0.95394    -9.132e-06
    'sex'                  0.029155      0.029792      0.97864    46    0.33288     -0.030812
    'age'                 -0.020037      0.019868      -1.0085    46    0.31849      -0.06003
    'drugOrder'            0.019894      0.028424      0.69991    46    0.48751      -0.03732
    'fiveDASC'          -0.00038654      0.011321    -0.034144    46    0.97291     -0.023174


    Upper
       0.15579
      0.028879
       0.17726
    9.6745e-06
      0.089123
      0.019956
      0.077108
      0.022401

Random effects covariance parameters (95% CIs):
Group: subject (27 Levels)
    Name1                Name2                Type         Estimate    Lower       Upper
    '(Intercept)'        '(Intercept)'        'std'        0.053708    0.029001    0.099465

Group: Error
    Name             Estimate    Lower       Upper
    'Res Std'        0.066301    0.049007    0.089699


 Contrast: cue loss vs neutral   ROI: striatum 5D-ASC subscore: asc_vis

lme =


Linear mixed-effects model fit by REML

Model information:
    Number of observations             54
    Fixed effects coefficients          8
    Random effects coefficients        27
    Covariance parameters               2

Formula:
    ROI ~ 1 + measurement + drug + ketLvl + sex + age + drugOrder + fiveDASC + (1 | subject)

Model fit statistics:
    AIC        BIC       LogLikelihood    Deviance
    -39.617    -21.33    29.808           -59.617

Fixed effects coefficients (95% CIs):
    Name                 Estimate      SE            tStat       DF    pValue     Lower
    '(Intercept)'          0.042415      0.057149     0.74218    46    0.46175       -0.07262
    'measurement'         -0.010548      0.019616    -0.53774    46    0.59335      -0.050033
    'drug'                -0.021725       0.09684    -0.22434    46    0.82349       -0.21665
    'ketLvl'             4.1435e-07    4.6454e-06    0.089195    46    0.92931    -8.9364e-06
    'sex'                  0.029379      0.029607     0.99231    46    0.32624      -0.030216
    'age'                  -0.01964      0.019663    -0.99882    46    0.32311       -0.05922
    'drugOrder'            0.020878      0.028114     0.74262    46    0.46149      -0.035713
    'fiveDASC'           -0.0035594      0.011305    -0.31485    46     0.7543      -0.026316


    Upper
       0.15745
      0.028936
        0.1732
    9.7651e-06
      0.088974
       0.01994
      0.077469
      0.019197

Random effects covariance parameters (95% CIs):
Group: subject (27 Levels)
    Name1                Name2                Type         Estimate    Lower       Upper
    '(Intercept)'        '(Intercept)'        'std'        0.052747    0.027758    0.10023

Group: Error
    Name             Estimate    Lower       Upper
    'Res Std'        0.06666     0.049113    0.090477
```

```
 Contrast: cue loss vs neutral   ROI: striatum 5D-ASC subscore: asc_aud

lme = 


Linear mixed-effects model fit by REML

Model information:
    Number of observations              54
    Fixed effects coefficients           8
    Random effects coefficients         27
    Covariance parameters                2

Formula:
    ROI ~ 1 + measurement + drug + ketLvl + sex + age + drugOrder + fiveDASC + (1 | subject)

Model fit statistics:
    AIC        BIC        LogLikelihood    Deviance
    -40.142    -21.855    30.071           -60.142 

Fixed effects coefficients (95% CIs):
    Name                   Estimate      SE            tStat       DF    pValue     Lower     
    '(Intercept)'            0.046059      0.057219     0.80496    46    0.42499     -0.069117
    'measurement'           -0.011758      0.018506    -0.63539    46    0.52832     -0.049008
    'drug'                  -0.027901       0.09678    -0.28829    46    0.77442      -0.22271
    'ketLvl'               7.1356e-07    4.6438e-06     0.15366    46    0.87855    -8.634e-06
    'sex'                    0.028996      0.029648     0.97802    46    0.33318     -0.030682
    'age'                   -0.021055      0.019696      -1.069    46    0.29064       -0.0607
    'drugOrder'              0.019592      0.027982     0.70016    46    0.48735     -0.036733
    'fiveDASC'             -0.0088593      0.011207     -0.7905    46    0.43329     -0.031418


    Upper     
       0.16123
      0.025492
       0.16691
    1.0061e-05
      0.088674
       0.01859
      0.075917
        0.0137

Random effects covariance parameters (95% CIs):
Group: subject (27 Levels)
    Name1                Name2                Type         Estimate    Lower       Upper   
    '(Intercept)'        '(Intercept)'        'std'        0.053627    0.029126    0.098739

Group: Error
    Name             Estimate    Lower       Upper   
    'Res Std'        0.065724    0.048623    0.088841


 Contrast: cue loss vs neutral   ROI: striatum 5D-ASC subscore: asc_vig

lme = 


Linear mixed-effects model fit by REML

Model information:
    Number of observations              54
    Fixed effects coefficients           8
    Random effects coefficients         27
    Covariance parameters                2

Formula:
    ROI ~ 1 + measurement + drug + ketLvl + sex + age + drugOrder + fiveDASC + (1 | subject)

Model fit statistics:
    AIC        BIC        LogLikelihood    Deviance
    -39.598    -21.312    29.799           -59.598 

Fixed effects coefficients (95% CIs):
    Name                   Estimate      SE            tStat       DF    pValue     Lower      
    '(Intercept)'            0.040508      0.057176     0.70848    46    0.48222      -0.074582
    'measurement'          -0.0076728      0.019187    -0.39989    46    0.69109      -0.046295
    'drug'                  -0.018874      0.096704    -0.19518    46    0.84611       -0.21353
```

```
    'ketLvl'             2.8045e-07    4.6403e-06    0.060438    46    0.95207    -9.0599e-06
    'sex'                  0.029123       0.02978     0.97794    46    0.33322      -0.030821
    'age'                 -0.020062      0.019748     -1.0159    46      0.315      -0.059812
    'drugOrder'            0.019244      0.028486     0.67555    46    0.50271      -0.038095
    'fiveDASC'            0.0012684      0.011666     0.10873    46    0.91389      -0.022214


    Upper
       0.1556
     0.030949
      0.17578
    9.6208e-06
     0.089068
     0.019689
     0.076582
     0.024751

Random effects covariance parameters (95% CIs):
Group: subject (27 Levels)
    Name1                Name2                Type         Estimate    Lower       Upper
    '(Intercept)'        '(Intercept)'        'std'        0.053667    0.028732    0.10024

Group: Error
    Name             Estimate    Lower       Upper
    'Res Std'        0.06631     0.048934    0.089857


 Contrast: cue loss vs neutral   ROI: striatum 5D-ASC subscore: sum

lme =


Linear mixed-effects model fit by REML

Model information:
    Number of observations             54
    Fixed effects coefficients          8
    Random effects coefficients        27
    Covariance parameters               2

Formula:
    ROI ~ 1 + measurement + drug + ketLvl + sex + age + drugOrder + fiveDASC + (1 | subject)

Model fit statistics:
    AIC        BIC        LogLikelihood    Deviance
    -39.598    -21.312    29.799           -59.598

Fixed effects coefficients (95% CIs):
    Name                 Estimate     SE            tStat       DF    pValue     Lower
    '(Intercept)'         0.040256      0.057373     0.70166    46    0.48642      -0.075229
    'measurement'        -0.007639      0.019265    -0.39653    46    0.69355      -0.046417
    'drug'               -0.016653      0.096796    -0.17204    46    0.86416       -0.21149
    'ketLvl'             1.721e-07    4.6437e-06    0.037061    46     0.9706    -9.1752e-06
    'sex'                 0.029136       0.02985     0.97608    46    0.33413      -0.030949
    'age'                -0.020266      0.019841     -1.0214    46     0.3124      -0.060204
    'drugOrder'           0.019347      0.028388     0.68151    46    0.49896      -0.037796
    'fiveDASC'           0.0012949      0.011655     0.11111    46    0.91201      -0.022165


    Upper
      0.15574
     0.031139
      0.17819
    9.5194e-06
     0.089222
     0.019672
      0.07649
     0.024755

Random effects covariance parameters (95% CIs):
Group: subject (27 Levels)
    Name1                Name2                Type         Estimate    Lower       Upper
    '(Intercept)'        '(Intercept)'        'std'        0.05396     0.029277    0.09945

Group: Error
    Name             Estimate    Lower       Upper
    'Res Std'        0.066171    0.048939    0.089472
```

```
 Contrast: cue loss vs neutral   ROI: nucleus accumbens 5D-ASC subscore: asc_obn

lme = 


Linear mixed-effects model fit by REML

Model information:
    Number of observations              54
    Fixed effects coefficients           8
    Random effects coefficients         27
    Covariance parameters                2

Formula:
    ROI ~ 1 + measurement + drug + ketLvl + sex + age + drugOrder + fiveDASC + (1 | subject)

Model fit statistics:
    AIC        BIC       LogLikelihood    Deviance
    -6.2941    11.992    13.147           -26.294 

Fixed effects coefficients (95% CIs):
    Name                   Estimate       SE            tStat       DF    pValue      Lower     
    '(Intercept)'              0.11604      0.080521      1.4411    46      0.15634     -0.046045
    'measurement'           -0.0073595      0.028001    -0.26283    46      0.79386     -0.063723
    'drug'                     0.22513       0.14196      1.5859    46      0.11961     -0.060615
    'ketLvl'               -1.2032e-05    6.8052e-06      -1.768    46     0.083689    -2.573e-05
    'sex'                    -0.020202      0.041084    -0.49172    46      0.62525       -0.1029
    'age'                    -0.046966       0.02724     -1.7242    46     0.091394       -0.1018
    'drugOrder'               0.043461      0.038692      1.1233    46      0.26716     -0.034422
    'fiveDASC'                0.029917      0.016153      1.8521    46     0.070439    -0.0025978


    Upper     
       0.27812
      0.049004
       0.51088
    1.6664e-06
      0.062496
      0.007865
       0.12134
      0.062432

Random effects covariance parameters (95% CIs):
Group: subject (27 Levels)
    Name1                Name2                Type         Estimate    Lower       Upper  
    '(Intercept)'        '(Intercept)'        'std'        0.068263    0.034035    0.13691

Group: Error
    Name             Estimate    Lower       Upper 
    'Res Std'        0.099171    0.074167    0.1326


 Contrast: cue loss vs neutral   ROI: nucleus accumbens 5D-ASC subscore: asc_aed

lme = 


Linear mixed-effects model fit by REML

Model information:
    Number of observations              54
    Fixed effects coefficients           8
    Random effects coefficients         27
    Covariance parameters                2

Formula:
    ROI ~ 1 + measurement + drug + ketLvl + sex + age + drugOrder + fiveDASC + (1 | subject)

Model fit statistics:
    AIC        BIC       LogLikelihood    Deviance
    -4.5257    13.761    12.263           -24.526 

Fixed effects coefficients (95% CIs):
    Name                   Estimate       SE            tStat       DF    pValue      Lower      
    '(Intercept)'              0.12658      0.080314       1.576    46      0.12187      -0.035085
    'measurement'              -0.0133       0.02955    -0.45008    46      0.65477      -0.072782
    'drug'                     0.20853        0.1483      1.4061    46      0.16641      -0.089989
```

```
    'ketLvl'             -1.1232e-05    7.1029e-06     -1.5813    46     0.12065    -2.5529e-05
    'sex'                   -0.01748      0.040062    -0.43633    46     0.66464      -0.098121
    'age'                  -0.045981       0.02654     -1.7326    46    0.089875      -0.099403
    'drugOrder'             0.041682       0.03801      1.0966    46     0.27852      -0.034829
    'fiveDASC'              0.019029      0.016999      1.1194    46     0.26876      -0.015188


    Upper
       0.28824
      0.046182
       0.50704
    3.0653e-06
       0.06316
       0.00744
       0.11819
      0.053246

Random effects covariance parameters (95% CIs):
Group: subject (27 Levels)
    Name1                Name2                Type         Estimate    Lower       Upper
    '(Intercept)'        '(Intercept)'        'std'        0.058517    0.023559    0.14535

Group: Error
    Name             Estimate    Lower       Upper
    'Res Std'        0.10592     0.079601    0.14093


 Contrast: cue loss vs neutral   ROI: nucleus accumbens 5D-ASC subscore: asc_vis

lme =


Linear mixed-effects model fit by REML

Model information:
    Number of observations              54
    Fixed effects coefficients           8
    Random effects coefficients         27
    Covariance parameters                2

Formula:
    ROI ~ 1 + measurement + drug + ketLvl + sex + age + drugOrder + fiveDASC + (1 | subject)

Model fit statistics:
    AIC        BIC       LogLikelihood    Deviance
    -11.553    6.7336    15.776           -31.553

Fixed effects coefficients (95% CIs):
    Name                 Estimate       SE            tStat       DF    pValue       Lower
    '(Intercept)'           0.10445       0.07661      1.3634    46       0.17938      -0.049754
    'measurement'          0.010256      0.027221     0.37678    46       0.70807      -0.044537
    'drug'                  0.21241       0.13295      1.5976    46       0.11697       -0.05521
    'ketLvl'            -1.1379e-05    6.3747e-06      -1.785    46      0.080853    -2.4211e-05
    'sex'                 -0.022646      0.039235    -0.57719    46       0.56663       -0.10162
    'age'                 -0.048929      0.026007     -1.8814    46      0.066254       -0.10128
    'drugOrder'            0.033752      0.037193     0.90746    46       0.36889      -0.041115
    'fiveDASC'             0.047822      0.015503      3.0847    46     0.0034419       0.016617


    Upper
       0.25866
       0.06505
       0.48003
    1.4527e-06
       0.05633
     0.0034196
       0.10862
      0.079028

Random effects covariance parameters (95% CIs):
Group: subject (27 Levels)
    Name1                Name2                Type         Estimate    Lower       Upper
    '(Intercept)'        '(Intercept)'        'std'        0.066717    0.034932    0.12743

Group: Error
    Name             Estimate    Lower       Upper
    'Res Std'        0.092692    0.069506    0.12361
```

Contrast: cue loss vs neutral   ROI: nucleus accumbens 5D-ASC subscore: asc_aud

```
lme =


Linear mixed-effects model fit by REML

Model information:
    Number of observations              54
    Fixed effects coefficients           8
    Random effects coefficients         27
    Covariance parameters                2

Formula:
    ROI ~ 1 + measurement + drug + ketLvl + sex + age + drugOrder + fiveDASC + (1 | subject)

Model fit statistics:
    AIC        BIC       LogLikelihood    Deviance
    -7.3959    10.891    13.698           -27.396

Fixed effects coefficients (95% CIs):
    Name                 Estimate       SE            tStat       DF    pValue      Lower
    '(Intercept)'            0.10665      0.079072      1.3488    46     0.18402      -0.052515
    'measurement'         -0.0068021      0.028219    -0.24104    46     0.81059      -0.063605
    'drug'                   0.23199       0.14246      1.6285    46     0.11025      -0.054762
    'ketLvl'             -1.2365e-05    6.8261e-06     -1.8115    46    0.076596    -2.6106e-05
    'sex'                  -0.016995      0.039592    -0.42924    46     0.66976       -0.09669
    'age'                  -0.038635      0.026148     -1.4776    46     0.14634      -0.091267
    'drugOrder'             0.049309      0.037125      1.3282    46     0.19067      -0.025419
    'fiveDASC'              0.033989      0.016214      2.0963    46    0.041581      0.0013529


    Upper
       0.26581
          0.05
       0.51875
    1.3748e-06
      0.062701
      0.013998
       0.12404
      0.066625

Random effects covariance parameters (95% CIs):
Group: subject (27 Levels)
    Name1                Name2                Type         Estimate    Lower       Upper
    '(Intercept)'        '(Intercept)'        'std'        0.061714    0.027832    0.13684

Group: Error
    Name             Estimate    Lower       Upper
    'Res Std'        0.10054     0.075421    0.13403
```

Contrast: cue loss vs neutral   ROI: nucleus accumbens 5D-ASC subscore: asc_vig

```
lme =


Linear mixed-effects model fit by REML

Model information:
    Number of observations              54
    Fixed effects coefficients           8
    Random effects coefficients         27
    Covariance parameters                2

Formula:
    ROI ~ 1 + measurement + drug + ketLvl + sex + age + drugOrder + fiveDASC + (1 | subject)

Model fit statistics:
    AIC        BIC      LogLikelihood    Deviance
    -3.7665    14.52    11.883           -23.766

Fixed effects coefficients (95% CIs):
    Name                 Estimate       SE            tStat       DF    pValue     Lower
    '(Intercept)'            0.12471       0.08147      1.5308    46    0.13268     -0.039279
    'measurement'          -0.013461      0.030314    -0.44407    46    0.65908     -0.074479
    'drug'                   0.18776       0.14753      1.2727    46    0.20953      -0.10921
```

```
    'ketLvl'            -1.0219e-05    7.0675e-06     -1.4459    46    0.15499    -2.4445e-05
    'sex'                 -0.018255      0.040882    -0.44652    46    0.65732       -0.10055
    'age'                 -0.041812      0.026929     -1.5527    46    0.12735      -0.096017
    'drugOrder'            0.043923      0.038934      1.1281    46    0.26512      -0.034448
    'fiveDASC'              0.01199      0.017415     0.68848    46    0.49461      -0.023065


    Upper
       0.2887
     0.047557
      0.48473
    4.0073e-06
     0.064037
     0.012392
      0.12229
     0.047045

Random effects covariance parameters (95% CIs):
Group: subject (27 Levels)
    Name1                Name2                Type         Estimate    Lower       Upper
    '(Intercept)'        '(Intercept)'        'std'        0.062174    0.026448    0.14616

Group: Error
    Name             Estimate    Lower       Upper
    'Res Std'        0.10553     0.079006    0.14096


 Contrast: cue loss vs neutral   ROI: nucleus accumbens 5D-ASC subscore: sum

lme =


Linear mixed-effects model fit by REML

Model information:
    Number of observations             54
    Fixed effects coefficients          8
    Random effects coefficients        27
    Covariance parameters               2

Formula:
    ROI ~ 1 + measurement + drug + ketLvl + sex + age + drugOrder + fiveDASC + (1 | subject)

Model fit statistics:
    AIC        BIC       LogLikelihood    Deviance
    -7.3337    10.953    13.667           -27.334

Fixed effects coefficients (95% CIs):
    Name                 Estimate       SE            tStat         DF    pValue
    '(Intercept)'            0.11332      0.080054        1.4156    46     0.16363
    'measurement'        -0.00011446      0.028241    -0.0040529    46     0.99678
    'drug'                   0.22856       0.13986        1.6342    46     0.10905
    'ketLvl'             -1.2186e-05    6.7055e-06       -1.8173    46    0.075684
    'sex'                  -0.019497      0.040981      -0.47577    46     0.63649
    'age'                  -0.046672      0.027162       -1.7183    46    0.092475
    'drugOrder'             0.037675      0.038879       0.96905    46     0.33759
    'fiveDASC'              0.035659      0.016698        2.1355    46     0.03808


    Lower          Upper
      -0.047817       0.27447
      -0.056961      0.056732
      -0.052968        0.5101
    -2.5684e-05    1.3114e-06
       -0.10199      0.062992
       -0.10135     0.0080031
      -0.040583       0.11593
      0.0020468      0.069271

Random effects covariance parameters (95% CIs):
Group: subject (27 Levels)
    Name1                Name2                Type         Estimate    Lower       Upper
    '(Intercept)'        '(Intercept)'        'std'        0.069396    0.035641    0.13512

Group: Error
    Name             Estimate    Lower       Upper
    'Res Std'        0.097324    0.072827    0.13006
```

```
 Contrast: cue loss vs neutral   ROI: insula 5D-ASC subscore: asc_obn

lme = 


Linear mixed-effects model fit by REML

Model information:
    Number of observations              54
    Fixed effects coefficients           8
    Random effects coefficients         27
    Covariance parameters                2

Formula:
    ROI ~ 1 + measurement + drug + ketLvl + sex + age + drugOrder + fiveDASC + (1 | subject)

Model fit statistics:
    AIC       BIC       LogLikelihood    Deviance
    24.594    42.881    -2.2972          4.5945  

Fixed effects coefficients (95% CIs):
    Name                     Estimate       SE            tStat        DF    pValue     Lower      
    '(Intercept)'               0.02959       0.12081      0.24492    46    0.80761        -0.2136
    'measurement'              0.030896      0.034671      0.89112    46     0.3775      -0.038893
    'drug'                      0.13751        0.1853      0.74212    46    0.46179       -0.23547
    'ketLvl'                -6.7791e-06    8.9011e-06      -0.7616    46    0.45018    -2.4696e-05
    'sex'                      0.041204       0.06493      0.63458    46    0.52884      -0.089494
    'age'                      -0.04093      0.043443     -0.94215    46    0.35104       -0.12838
    'drugOrder'              -0.0038671      0.061774    -0.062601    46    0.95036       -0.12821
    'fiveDASC'                 0.011742      0.021344      0.55013    46    0.58489      -0.031221


    Upper     
       0.27278
       0.10069
        0.5105
    1.1138e-05
        0.1719
      0.046517
       0.12048
      0.054705

Random effects covariance parameters (95% CIs):
Group: subject (27 Levels)
    Name1                Name2                Type         Estimate    Lower       Upper  
    '(Intercept)'        '(Intercept)'        'std'        0.13038     0.083441    0.20373

Group: Error
    Name             Estimate    Lower       Upper  
    'Res Std'        0.12215     0.091409    0.16322


 Contrast: cue loss vs neutral   ROI: insula 5D-ASC subscore: asc_aed

lme = 


Linear mixed-effects model fit by REML

Model information:
    Number of observations              54
    Fixed effects coefficients           8
    Random effects coefficients         27
    Covariance parameters                2

Formula:
    ROI ~ 1 + measurement + drug + ketLvl + sex + age + drugOrder + fiveDASC + (1 | subject)

Model fit statistics:
    AIC       BIC       LogLikelihood    Deviance
    24.809    43.095    -2.4043          4.8086  

Fixed effects coefficients (95% CIs):
    Name                     Estimate       SE            tStat         DF    pValue     Lower      
    '(Intercept)'              0.034019       0.12103       0.28108    46    0.77991        -0.2096
    'measurement'              0.024401      0.034332       0.71073    46    0.48084      -0.044706
    'drug'                      0.12544       0.18615       0.67387    46    0.50377       -0.24926
```

```
    'ketLvl'            -6.2016e-06    8.9428e-06      -0.69347    46     0.4915    -2.4202e-05
    'sex'                  0.042369      0.065181       0.65002    46    0.51891      -0.088834
    'age'                 -0.038187      0.043709      -0.87367    46    0.38684       -0.12617
    'drugOrder'         -0.00020684      0.062473    -0.0033108    46    0.99737       -0.12596
    'fiveDASC'           -0.0044016      0.021901      -0.20097    46     0.8416      -0.048486


    Upper
       0.27764
      0.093508
       0.50014
    1.1799e-05
       0.17357
      0.049794
       0.12554
      0.039683

Random effects covariance parameters (95% CIs):
Group: subject (27 Levels)
    Name1                Name2                Type         Estimate    Lower       Upper
    '(Intercept)'        '(Intercept)'        'std'        0.13116     0.083912    0.20502

Group: Error
    Name             Estimate    Lower      Upper
    'Res Std'        0.1223      0.09146    0.16353


 Contrast: cue loss vs neutral   ROI: insula 5D-ASC subscore: asc_vis

lme =


Linear mixed-effects model fit by REML

Model information:
    Number of observations             54
    Fixed effects coefficients          8
    Random effects coefficients        27
    Covariance parameters               2

Formula:
    ROI ~ 1 + measurement + drug + ketLvl + sex + age + drugOrder + fiveDASC + (1 | subject)

Model fit statistics:
    AIC       BIC       LogLikelihood    Deviance
    24.861    43.148    -2.4307          4.8614

Fixed effects coefficients (95% CIs):
    Name                Estimate       SE            tStat        DF    pValue     Lower
    '(Intercept)'          0.033023       0.12133      0.27217    46    0.78671       -0.21121
    'measurement'          0.027124      0.036318      0.74686    46    0.45895       -0.04598
    'drug'                  0.12877       0.18531      0.69491    46     0.4906       -0.24423
    'ketLvl'            -6.3593e-06    8.9015e-06     -0.71441    46    0.47859    -2.4277e-05
    'sex'                  0.042181      0.065116      0.64777    46    0.52035      -0.088892
    'age'                 -0.039318       0.04353     -0.90324    46    0.37111       -0.12694
    'drugOrder'          -0.0024181      0.062189    -0.038883    46    0.96915        -0.1276
    'fiveDASC'            0.0018906      0.021712     0.087076    46    0.93099      -0.041813


    Upper
       0.27725
       0.10023
       0.50177
    1.1559e-05
       0.17325
      0.048303
       0.12276
      0.045594

Random effects covariance parameters (95% CIs):
Group: subject (27 Levels)
    Name1                Name2                Type         Estimate    Lower       Upper
    '(Intercept)'        '(Intercept)'        'std'        0.13064     0.083453    0.20452

Group: Error
    Name             Estimate    Lower       Upper
    'Res Std'        0.12261     0.091689    0.16396
```

Contrast: cue loss vs neutral   ROI: insula 5D-ASC subscore: asc_aud

lme =

Linear mixed-effects model fit by REML

Model information:

Number of observations	54
Fixed effects coefficients	8
Random effects coefficients	27
Covariance parameters	2

Formula:
ROI ~ 1 + measurement + drug + ketLvl + sex + age + drugOrder + fiveDASC + (1 | subject)

Model fit statistics:

AIC	BIC	LogLikelihood	Deviance
24.474	42.761	-2.2371	4.4743

Fixed effects coefficients (95% CIs):

Name	Estimate	SE	tStat	DF	pValue	Lower
'(Intercept)'	0.04185	0.12101	0.34584	46	0.73104	-0.20173
'measurement'	0.02087	0.034566	0.60376	46	0.54897	-0.048709
'drug'	0.11148	0.18673	0.59701	46	0.55343	-0.26438
'ketLvl'	-5.5267e-06	8.9707e-06	-0.61608	46	0.54088	-2.3584e-05
'sex'	0.041948	0.064702	0.64833	46	0.52	-0.08829
'age'	-0.040494	0.043237	-0.93656	46	0.35387	-0.12753
'drugOrder'	-0.0020491	0.06147	-0.033334	46	0.97355	-0.12578
'fiveDASC'	-0.013271	0.022006	-0.60305	46	0.54944	-0.057567

Upper
0.28543
0.090449
0.48734
1.253e-05
0.17219
0.046538
0.12168
0.031025

Random effects covariance parameters (95% CIs):
Group: subject (27 Levels)

Name1	Name2	Type	Estimate	Lower	Upper
'(Intercept)'	'(Intercept)'	'std'	0.12969	0.082718	0.20332

Group: Error

Name	Estimate	Lower	Upper
'Res Std'	0.12236	0.091592	0.16347

Contrast: cue loss vs neutral   ROI: insula 5D-ASC subscore: asc_vig

lme =

Linear mixed-effects model fit by REML

Model information:

Number of observations	54
Fixed effects coefficients	8
Random effects coefficients	27
Covariance parameters	2

Formula:
ROI ~ 1 + measurement + drug + ketLvl + sex + age + drugOrder + fiveDASC + (1 | subject)

Model fit statistics:

AIC	BIC	LogLikelihood	Deviance
24.529	42.816	-2.2645	4.5291

Fixed effects coefficients (95% CIs):

Name	Estimate	SE	tStat	DF	pValue	Lower
'(Intercept)'	0.03179	0.12057	0.26366	46	0.79322	-0.21091
'measurement'	0.031861	0.035704	0.89236	46	0.37685	-0.040008
'drug'	0.12211	0.18537	0.65876	46	0.51334	-0.25102

'ketLvl'	-6.0258e-06	8.9068e-06	-0.67654	46	0.50208	-2.3954e-05
'sex'	0.041895	0.064823	0.6463	46	0.5213	-0.088587
'age'	-0.038633	0.04326	-0.89302	46	0.37649	-0.12571
'drugOrder'	-0.0062552	0.062253	-0.10048	46	0.9204	-0.13156
'fiveDASC'	0.011097	0.022835	0.48597	46	0.62929	-0.034868

Upper
0.27449
0.10373
0.49525
1.1903e-05
0.17238
0.048446
0.11905
0.057063

Random effects covariance parameters (95% CIs):
Group: subject (27 Levels)

Name1	Name2	Type	Estimate	Lower	Upper
'(Intercept)'	'(Intercept)'	'std'	0.12998	0.082998	0.20356

Group: Error

Name	Estimate	Lower	Upper
'Res Std'	0.12247	0.091715	0.16355

Contrast: cue loss vs neutral   ROI: insula 5D-ASC subscore: sum

lme =

Linear mixed-effects model fit by REML

Model information:

Number of observations	54
Fixed effects coefficients	8
Random effects coefficients	27
Covariance parameters	2

Formula:
ROI ~ 1 + measurement + drug + ketLvl + sex + age + drugOrder + fiveDASC + (1 | subject)

Model fit statistics:

AIC	BIC	LogLikelihood	Deviance
24.483	42.769	-2.2414	4.4828

Fixed effects coefficients (95% CIs):

Name	Estimate	SE	tStat	DF	pValue	Lower
'(Intercept)'	0.029185	0.12103	0.24113	46	0.81052	-0.21444
'measurement'	0.032796	0.035761	0.9171	46	0.36387	-0.039186
'drug'	0.13644	0.18485	0.73811	46	0.4642	-0.23564
'ketLvl'	-6.7232e-06	8.8792e-06	-0.75719	46	0.4528	-2.4596e-05
'sex'	0.041509	0.06504	0.6382	46	0.52651	-0.08941
'age'	-0.04061	0.043493	-0.93372	46	0.35532	-0.12816
'drugOrder'	-0.005597	0.062177	-0.090016	46	0.92867	-0.13075
'fiveDASC'	0.012264	0.022667	0.54108	46	0.59107	-0.033361

Upper
0.27281
0.10478
0.50852
1.115e-05
0.17243
0.046936
0.11956
0.05789

Random effects covariance parameters (95% CIs):
Group: subject (27 Levels)

Name1	Name2	Type	Estimate	Lower	Upper
'(Intercept)'	'(Intercept)'	'std'	0.13086	0.083938	0.204

Group: Error

Name	Estimate	Lower	Upper
'Res Std'	0.12194	0.091317	0.16282

```
 Contrast: cue loss vs neutral   ROI: thalamus 5D-ASC subscore: asc_obn

lme = 


Linear mixed-effects model fit by REML

Model information:
    Number of observations              54
    Fixed effects coefficients           8
    Random effects coefficients         27
    Covariance parameters                2

Formula:
    ROI ~ 1 + measurement + drug + ketLvl + sex + age + drugOrder + fiveDASC + (1 | subject)

Model fit statistics:
    AIC        BIC        LogLikelihood    Deviance
    -40.505    -22.218    30.252           -60.505  

Fixed effects coefficients (95% CIs):
    Name                   Estimate       SE            tStat       DF    pValue      Lower      
    '(Intercept)'            0.038455      0.056741     0.67773    46     0.50134       -0.075759
    'measurement'             0.01482      0.018502       0.801    46     0.42725       -0.022422
    'drug'                    0.19305      0.095789      2.0153    46    0.049733      0.00023495
    'ketLvl'              -8.5102e-06    4.5958e-06     -1.8517    46    0.070488     -1.7761e-05
    'sex'                    0.040395      0.029558      1.3667    46     0.17838       -0.019101
    'age'                   -0.010354      0.019665    -0.52653    46     0.60105       -0.049938
    'drugOrder'              0.021838      0.027944      0.7815    46     0.43851        -0.03441
    'fiveDASC'              0.0092088      0.010951     0.84091    46     0.40475       -0.012834


    Upper     
       0.15267
      0.052061
       0.38586
    7.4062e-07
      0.099891
       0.02923
      0.078086
      0.031252

Random effects covariance parameters (95% CIs):
Group: subject (27 Levels)
    Name1                  Name2                Type         Estimate    Lower       Upper   
    '(Intercept)'          '(Intercept)'        'std'        0.053486    0.030146    0.094897

Group: Error
    Name             Estimate    Lower       Upper   
    'Res Std'        0.065396    0.048861    0.087526


 Contrast: cue loss vs neutral   ROI: thalamus 5D-ASC subscore: asc_aed

lme = 


Linear mixed-effects model fit by REML

Model information:
    Number of observations              54
    Fixed effects coefficients           8
    Random effects coefficients         27
    Covariance parameters                2

Formula:
    ROI ~ 1 + measurement + drug + ketLvl + sex + age + drugOrder + fiveDASC + (1 | subject)

Model fit statistics:
    AIC        BIC        LogLikelihood    Deviance
    -40.341    -22.054    30.17            -60.341  

Fixed effects coefficients (95% CIs):
    Name                   Estimate       SE            tStat       DF    pValue      Lower      
    '(Intercept)'             0.04169      0.056739     0.73476    46     0.46622        -0.07252
    'measurement'            0.013596      0.018341     0.74133    46     0.46226       -0.023321
    'drug'                    0.19446      0.096344      2.0184    46    0.049401      0.00053152
```

```
    'ketLvl'            -8.5814e-06    4.6229e-06     -1.8563    46    0.069827    -1.7887e-05
    'sex'                  0.041638      0.029619      1.4058    46      0.1665      -0.017982
    'age'                 -0.010391      0.019757    -0.52592    46     0.60147       -0.05016
    'drugOrder'              0.0205      0.028264     0.72532    46     0.47193      -0.036391
    'fiveDASC'            0.0079039      0.011208     0.70522    46     0.48423      -0.014656


    Upper
       0.1559
     0.050514
      0.38839
    7.2408e-07
      0.10126
     0.029378
     0.077392
     0.030464

Random effects covariance parameters (95% CIs):
Group: subject (27 Levels)
    Name1                Name2                Type         Estimate    Lower       Upper
    '(Intercept)'        '(Intercept)'        'std'        0.053671    0.030272    0.095156

Group: Error
    Name             Estimate    Lower       Upper
    'Res Std'        0.065512    0.048951    0.087676


 Contrast: cue loss vs neutral   ROI: thalamus 5D-ASC subscore: asc_vis

lme =


Linear mixed-effects model fit by REML

Model information:
    Number of observations             54
    Fixed effects coefficients          8
    Random effects coefficients        27
    Covariance parameters               2

Formula:
    ROI ~ 1 + measurement + drug + ketLvl + sex + age + drugOrder + fiveDASC + (1 | subject)

Model fit statistics:
    AIC        BIC       LogLikelihood    Deviance
    -40.466    -22.18    30.233           -60.466

Fixed effects coefficients (95% CIs):
    Name                 Estimate       SE            tStat       DF    pValue      Lower
    '(Intercept)'           0.037717      0.057125     0.66025    46     0.51238      -0.07727
    'measurement'           0.016441      0.019133     0.85929    46     0.39464     -0.022072
    'drug'                   0.18927      0.095112        1.99    46    0.052559    -0.0021823
    'ketLvl'             -8.3229e-06    4.5639e-06     -1.8236    46    0.074709    -1.751e-05
    'sex'                   0.040437      0.029815      1.3563    46     0.18163     -0.019577
    'age'                    -0.0101      0.019827    -0.50939    46     0.61292      -0.05001
    'drugOrder'             0.020696      0.028344     0.73016    46     0.46899     -0.036358
    'fiveDASC'             0.0087621      0.011112     0.78853    46     0.43443     -0.013605


    Upper
      0.1527
    0.054953
     0.38072
    8.638e-07
     0.10045
    0.029811
     0.07775
    0.031129

Random effects covariance parameters (95% CIs):
Group: subject (27 Levels)
    Name1                Name2                Type         Estimate    Lower       Upper
    '(Intercept)'        '(Intercept)'        'std'        0.054611    0.031472    0.094763

Group: Error
    Name             Estimate    Lower       Upper
    'Res Std'        0.064932    0.048589    0.086773
```

Contrast: cue loss vs neutral   ROI: thalamus 5D-ASC subscore: asc_aud

lme =

Linear mixed-effects model fit by REML

Model information:

Number of observations	54
Fixed effects coefficients	8
Random effects coefficients	27
Covariance parameters	2

Formula:

ROI ~ 1 + measurement + drug + ketLvl + sex + age + drugOrder + fiveDASC + (1 | subject)

Model fit statistics:

AIC	BIC	LogLikelihood	Deviance
-40.285	-21.999	30.142	-60.285

Fixed effects coefficients (95% CIs):

Name	Estimate	SE	tStat	DF	pValue	Lower	Upper
'(Intercept)'	0.037498	0.057548	0.6516	46	0.5179	-0.07834	0.15334
'measurement'	0.01365	0.018236	0.74851	46	0.45796	-0.023057	0.050356
'drug'	0.19812	0.095955	2.0648	46	0.044609	0.0049768	0.39127
'ketLvl'	-8.7601e-06	4.6054e-06	-1.9022	46	0.063423	-1.803e-05	5.1001e-07
'sex'	0.041803	0.029996	1.3936	46	0.17012	-0.018575	0.10218
'age'	-0.0080488	0.019948	-0.40348	46	0.68846	-0.048203	0.032105
'drugOrder'	0.023537	0.028345	0.83039	46	0.41061	-0.033518	0.080592
'fiveDASC'	0.0073119	0.011147	0.65593	46	0.51514	-0.015126	0.02975

Random effects covariance parameters (95% CIs):

Group: subject (27 Levels)

Name1	Name2	Type	Estimate	Lower	Upper
'(Intercept)'	'(Intercept)'	'std'	0.055429	0.03228	0.095179

Group: Error

Name	Estimate	Lower	Upper
'Res Std'	0.064728	0.048486	0.086409

Contrast: cue loss vs neutral   ROI: thalamus 5D-ASC subscore: asc_vig

lme =

Linear mixed-effects model fit by REML

Model information:

Number of observations	54
Fixed effects coefficients	8
Random effects coefficients	27
Covariance parameters	2

Formula:

ROI ~ 1 + measurement + drug + ketLvl + sex + age + drugOrder + fiveDASC + (1 | subject)

Model fit statistics:

AIC	BIC	LogLikelihood	Deviance
-41.141	-22.854	30.57	-61.141

Fixed effects coefficients (95% CIs):

Name	Estimate	SE	tStat	DF	pValue	Lower
'(Intercept)'	0.039449	0.056617	0.69676	46	0.48946	-0.074516
'measurement'	0.017617	0.01865	0.94463	46	0.34978	-0.019923
'drug'	0.18367	0.09448	1.944	46	0.058025	-0.0065084

```
    'ketLvl'            -8.0475e-06    4.5346e-06    -1.7747    46    0.082567    -1.7175e-05
    'sex'                 0.041131      0.029652      1.3871    46     0.17208      -0.018554
    'age'               -0.0083417      0.019683     -0.4238    46     0.67369      -0.047962
    'drugOrder'           0.018333      0.028382     0.64595    46     0.52152      -0.038796
    'fiveDASC'            0.012708      0.011437      1.1111    46     0.27229      -0.010314


    Upper
       0.15341
      0.055157
       0.37385
    1.0802e-06
       0.10082
      0.031279
      0.075463
       0.03573

Random effects covariance parameters (95% CIs):
Group: subject (27 Levels)
    Name1               Name2               Type         Estimate    Lower       Upper
    '(Intercept)'       '(Intercept)'       'std'        0.054537    0.031667    0.093924

Group: Error
    Name             Estimate    Lower       Upper
    'Res Std'        0.064374    0.048273    0.085847


 Contrast: cue loss vs neutral   ROI: thalamus 5D-ASC subscore: sum

lme =


Linear mixed-effects model fit by REML

Model information:
    Number of observations             54
    Fixed effects coefficients          8
    Random effects coefficients        27
    Covariance parameters               2

Formula:
    ROI ~ 1 + measurement + drug + ketLvl + sex + age + drugOrder + fiveDASC + (1 | subject)

Model fit statistics:
    AIC        BIC        LogLikelihood    Deviance
    -41.248    -22.961    30.624           -61.248

Fixed effects coefficients (95% CIs):
    Name                Estimate       SE            tStat       DF    pValue      Lower
    '(Intercept)'          0.03668      0.056332     0.65115    46     0.51819       -0.07671
    'measurement'         0.018431      0.018935     0.97336    46     0.33547      -0.019684
    'drug'                 0.19409      0.095112      2.0406    46     0.04705      0.0026389
    'ketLvl'            -8.5545e-06    4.5629e-06    -1.8748    46    0.067177    -1.7739e-05
    'sex'                 0.040316      0.029299       1.376    46     0.17548       -0.01866
    'age'                -0.010589      0.019474    -0.54377    46     0.58922      -0.049788
    'drugOrder'           0.019329      0.027863     0.69371    46     0.49135      -0.036757
    'fiveDASC'            0.013384       0.01145      1.1689    46     0.24846     -0.0096635


    Upper
      0.15007
     0.056545
      0.38554
    6.301e-07
     0.099293
     0.028609
     0.075414
     0.036432

Random effects covariance parameters (95% CIs):
Group: subject (27 Levels)
    Name1               Name2               Type         Estimate    Lower       Upper
    '(Intercept)'       '(Intercept)'       'std'        0.0529      0.029796    0.093919

Group: Error
    Name             Estimate    Lower       Upper
    'Res Std'        0.065043    0.048665    0.086935
```

Contrast: cue loss vs neutral ROI: amygdala 5D-ASC subscore: asc_obn

lme =

Linear mixed-effects model fit by REML

Model information:

Number of observations	54
Fixed effects coefficients	8
Random effects coefficients	27
Covariance parameters	2

Formula:
ROI ~ 1 + measurement + drug + ketLvl + sex + age + drugOrder + fiveDASC + (1 | subject)

Model fit statistics:

AIC	BIC	LogLikelihood	Deviance
15.384	33.671	2.3079	-4.6159

Fixed effects coefficients (95% CIs):

Name	Estimate	SE	tStat	DF	pValue	Lower
'(Intercept)'	0.23991	0.10333	2.3217	46	0.024731	0.031911
'measurement'	-0.048404	0.034477	-1.4039	46	0.16706	-0.1178
'drug'	0.18464	0.17725	1.0417	46	0.303	-0.17214
'ketLvl'	-1.1744e-05	8.5017e-06	-1.3813	46	0.17385	-2.8857e-05
'sex'	0.020337	0.053456	0.38045	46	0.70536	-0.087265
'age'	-0.052467	0.035524	-1.477	46	0.1465	-0.12397
'drugOrder'	-0.079395	0.050472	-1.5731	46	0.12256	-0.18099
'fiveDASC'	-0.027314	0.020232	-1.3501	46	0.18359	-0.068038

Upper
0.44792
0.020996
0.54142
5.3695e-06
0.12794
0.019038
0.022199
0.01341

Random effects covariance parameters (95% CIs):
Group: subject (27 Levels)

Name1	Name2	Type	Estimate	Lower	Upper
'(Intercept)'	'(Intercept)'	'std'	0.094174	0.049265	0.18002

Group: Error

Name	Estimate	Lower	Upper
'Res Std'	0.12195	0.090175	0.16491

Contrast: cue loss vs neutral ROI: amygdala 5D-ASC subscore: asc_aed

lme =

Linear mixed-effects model fit by REML

Model information:

Number of observations	54
Fixed effects coefficients	8
Random effects coefficients	27
Covariance parameters	2

Formula:
ROI ~ 1 + measurement + drug + ketLvl + sex + age + drugOrder + fiveDASC + (1 | subject)

Model fit statistics:

AIC	BIC	LogLikelihood	Deviance
15.99	34.277	2.0048	-4.0096

Fixed effects coefficients (95% CIs):

Name	Estimate	SE	tStat	DF	pValue	Lower
'(Intercept)'	0.23039	0.10532	2.1875	46	0.033824	0.018392
'measurement'	-0.044283	0.033456	-1.3236	46	0.19217	-0.11163
'drug'	0.20037	0.17661	1.1345	46	0.26244	-0.15513

```
    'ketLvl'            -1.2504e-05    8.4761e-06    -1.4752    46     0.14697    -2.9566e-05
    'sex'                  0.018053      0.055247    0.32677    46     0.74533      -0.093153
    'age'                 -0.052627      0.036881    -1.4269    46     0.16035       -0.12687
    'drugOrder'           -0.076471      0.052754    -1.4496    46     0.15396       -0.18266
    'fiveDASC'            -0.020959       0.02058    -1.0184    46     0.31381      -0.062384


    Upper
       0.44239
       0.02306
       0.55587
    4.5574e-06
       0.12926
      0.021611
      0.029718
      0.020467

Random effects covariance parameters (95% CIs):
Group: subject (27 Levels)
    Name1                Name2                Type         Estimate    Lower      Upper
    '(Intercept)'        '(Intercept)'        'std'        0.10193     0.05834    0.17809

Group: Error
    Name             Estimate    Lower       Upper
    'Res Std'        0.11945     0.089006    0.16032


 Contrast: cue loss vs neutral   ROI: amygdala 5D-ASC subscore: asc_vis

lme =


Linear mixed-effects model fit by REML

Model information:
    Number of observations             54
    Fixed effects coefficients          8
    Random effects coefficients        27
    Covariance parameters               2

Formula:
    ROI ~ 1 + measurement + drug + ketLvl + sex + age + drugOrder + fiveDASC + (1 | subject)

Model fit statistics:
    AIC       BIC       LogLikelihood    Deviance
    14.935    33.221    2.5326           -5.0653

Fixed effects coefficients (95% CIs):
    Name                 Estimate       SE            tStat      DF    pValue      Lower
    '(Intercept)'           0.24393       0.10536     2.3151    46    0.025122       0.031841
    'measurement'          -0.05599      0.034349      -1.63    46     0.10993       -0.12513
    'drug'                  0.22644       0.17197     1.3167    46     0.19445       -0.11971
    'ketLvl'            -1.3797e-05    8.2542e-06    -1.6715    46     0.10141    -3.0412e-05
    'sex'                  0.022976      0.055408    0.41467    46     0.68031      -0.088555
    'age'                 -0.052367      0.036898    -1.4193    46     0.16257       -0.12664
    'drugOrder'           -0.075172      0.052739    -1.4254    46     0.16081       -0.18133
    'fiveDASC'            -0.029378      0.020106    -1.4611    46     0.15078      -0.069849


    Upper
       0.45601
      0.013151
       0.57258
    2.8177e-06
       0.13451
      0.021904
      0.030986
      0.011094

Random effects covariance parameters (95% CIs):
Group: subject (27 Levels)
    Name1                Name2                Type         Estimate    Lower       Upper
    '(Intercept)'        '(Intercept)'        'std'        0.10422     0.061557    0.17646

Group: Error
    Name             Estimate    Lower       Upper
    'Res Std'        0.11641     0.086866    0.15601
```

Contrast: cue loss vs neutral   ROI: amygdala 5D-ASC subscore: asc_aud

lme =

Linear mixed-effects model fit by REML

Model information:

Number of observations	54
Fixed effects coefficients	8
Random effects coefficients	27
Covariance parameters	2

Formula:
ROI ~ 1 + measurement + drug + ketLvl + sex + age + drugOrder + fiveDASC + (1 | subject)

Model fit statistics:

AIC	BIC	LogLikelihood	Deviance
13.204	31.49	3.398	-6.796

Fixed effects coefficients (95% CIs):

Name	Estimate	SE	tStat	DF	pValue	Lower	Upper
'(Intercept)'	0.2542	0.10144	2.5058	46	0.015817	0.050006	0.45839
'measurement'	-0.052435	0.033497	-1.5654	46	0.12435	-0.11986	0.014991
'drug'	0.15843	0.17403	0.91038	46	0.36737	-0.19187	0.50873
'ketLvl'	-1.0472e-05	8.3482e-06	-1.2544	46	0.21604	-2.7276e-05	6.3323e-06
'sex'	0.016501	0.052226	0.31596	46	0.75346	-0.088624	0.12163
'age'	-0.061216	0.034655	-1.7664	46	0.083957	-0.13097	0.0085409
'drugOrder'	-0.084841	0.049229	-1.7234	46	0.091533	-0.18393	0.014252
'fiveDASC'	-0.040682	0.020084	-2.0255	46	0.048637	-0.08111	-0.0002539

Random effects covariance parameters (95% CIs):
Group: subject (27 Levels)

Name1	Name2	Type	Estimate	Lower	Upper
'(Intercept)'	'(Intercept)'	'std'	0.092198	0.04917	0.17288

Group: Error

Name	Estimate	Lower	Upper
'Res Std'	0.11904	0.088512	0.1601

Contrast: cue loss vs neutral   ROI: amygdala 5D-ASC subscore: asc_vig

lme =

Linear mixed-effects model fit by REML

Model information:

Number of observations	54
Fixed effects coefficients	8
Random effects coefficients	27
Covariance parameters	2

Formula:
ROI ~ 1 + measurement + drug + ketLvl + sex + age + drugOrder + fiveDASC + (1 | subject)

Model fit statistics:

AIC	BIC	LogLikelihood	Deviance
14.58	32.867	2.7099	-5.4199

Fixed effects coefficients (95% CIs):

Name	Estimate	SE	tStat	DF	pValue	Lower
'(Intercept)'	0.23621	0.10291	2.2953	46	0.026327	0.029061
'measurement'	-0.054603	0.034645	-1.5761	46	0.12186	-0.12434
'drug'	0.22407	0.17445	1.2844	46	0.20543	-0.12709

```
    'ketLvl'            -1.3679e-05    8.3706e-06    -1.6342    46     0.10905    -3.0528e-05
    'sex'                  0.019021       0.05355    0.35521    46     0.72406      -0.088769
    'age'                 -0.058099      0.035503    -1.6364    46     0.10857       -0.12956
    'drugOrder'           -0.070877      0.051216    -1.3839    46     0.17308       -0.17397
    'fiveDASC'            -0.033236      0.021033    -1.5802    46     0.12092      -0.075572


    Upper
       0.44336
      0.015133
       0.57522
    3.1701e-06
       0.12681
      0.013365
      0.032216
     0.0091014

Random effects covariance parameters (95% CIs):
Group: subject (27 Levels)
    Name1                Name2                Type         Estimate    Lower       Upper
    '(Intercept)'        '(Intercept)'        'std'        0.096147    0.052164    0.17722

Group: Error
    Name             Estimate    Lower       Upper
    'Res Std'        0.11975     0.088863    0.16139


 Contrast: cue loss vs neutral   ROI: amygdala 5D-ASC subscore: sum

lme =


Linear mixed-effects model fit by REML

Model information:
    Number of observations             54
    Fixed effects coefficients          8
    Random effects coefficients        27
    Covariance parameters               2

Formula:
    ROI ~ 1 + measurement + drug + ketLvl + sex + age + drugOrder + fiveDASC + (1 | subject)

Model fit statistics:
    AIC       BIC       LogLikelihood    Deviance
    14.339    32.625    2.8307           -5.6615

Fixed effects coefficients (95% CIs):
    Name                Estimate       SE            tStat      DF    pValue      Lower
    '(Intercept)'          0.24348        0.1023       2.38     46    0.021515       0.037554
    'measurement'        -0.056628      0.035109    -1.6129     46      0.1136        -0.1273
    'drug'                 0.18231       0.17533     1.0398     46     0.30388       -0.17062
    'ketLvl'            -1.1645e-05    8.4092e-06    -1.3847    46     0.17281    -2.8571e-05
    'sex'                  0.020095      0.052863    0.38013    46      0.7056      -0.086313
    'age'                 -0.052351      0.035095    -1.4917    46     0.14261       -0.12299
    'drugOrder'           -0.073278      0.050222    -1.4591    46     0.15134       -0.17437
    'fiveDASC'            -0.035336      0.021035    -1.6799    46     0.09976      -0.077676


    Upper
        0.4494
      0.014043
       0.53523
    5.2822e-06
        0.1265
      0.018292
      0.027813
     0.0070047

Random effects covariance parameters (95% CIs):
Group: subject (27 Levels)
    Name1                Name2                Type         Estimate    Lower      Upper
    '(Intercept)'        '(Intercept)'        'std'        0.093066    0.04875    0.17766

Group: Error
    Name             Estimate    Lower       Upper
    'Res Std'        0.12076     0.089428    0.16308
```

```
 Contrast: fb success vs fb neutral      ROI: striatum 5D-ASC subscore: asc_obn

lme = 


Linear mixed-effects model fit by REML

Model information:
    Number of observations              54
    Fixed effects coefficients           8
    Random effects coefficients         27
    Covariance parameters                2

Formula:
    ROI ~ 1 + measurement + drug + ketLvl + sex + age + drugOrder + fiveDASC + (1 | subject)

Model fit statistics:
    AIC       BIC       LogLikelihood    Deviance 
    19.948    38.234    0.026191         -0.052381

Fixed effects coefficients (95% CIs):
    Name                    Estimate       SE            tStat        DF    pValue     Lower     
    '(Intercept)'              -0.1758       0.10692      -1.6442    46    0.10695       -0.39102
    'measurement'             0.047031      0.037375       1.2583    46    0.21462      -0.028201
    'drug'                     0.10409       0.18913       0.5504    46    0.58471        -0.2766
    'ketLvl'               -7.3654e-06    9.0655e-06     -0.81246    46    0.42071    -2.5613e-05
    'sex'                    -0.001882      0.054452    -0.034561    46    0.97258       -0.11149
    'age'                     0.025286      0.036093      0.70059    46    0.48709      -0.047365
    'drugOrder'               0.045194      0.051265      0.88157    46    0.38259      -0.057997
    'fiveDASC'                0.024527      0.021511       1.1402    46     0.2601      -0.018773


    Upper     
      0.039419
       0.12226
       0.48478
    1.0883e-05
       0.10772
      0.097937
       0.14838
      0.067828

Random effects covariance parameters (95% CIs):
Group: subject (27 Levels)
    Name1                Name2                Type         Estimate    Lower       Upper  
    '(Intercept)'        '(Intercept)'        'std'        0.08971     0.043469    0.18514

Group: Error
    Name             Estimate    Lower       Upper  
    'Res Std'        0.13239     0.098684    0.17762


 Contrast: fb success vs fb neutral      ROI: striatum 5D-ASC subscore: asc_aed

lme = 


Linear mixed-effects model fit by REML

Model information:
    Number of observations              54
    Fixed effects coefficients           8
    Random effects coefficients         27
    Covariance parameters                2

Formula:
    ROI ~ 1 + measurement + drug + ketLvl + sex + age + drugOrder + fiveDASC + (1 | subject)

Model fit statistics:
    AIC       BIC       LogLikelihood    Deviance 
    20.006    38.293    -0.0030476       0.0060952

Fixed effects coefficients (95% CIs):
    Name                    Estimate      SE            tStat        DF    pValue     Lower     
    '(Intercept)'             -0.16744       0.10863      -1.5415    46    0.13006        -0.3861
    'measurement'             0.046175      0.035769       1.2909    46    0.20318      -0.025824
    'drug'                    0.095017       0.18687      0.50848    46    0.61355       -0.28112
```

```
    'ketLvl'            -6.924e-06    8.9646e-06     -0.77237    46    0.44385    -2.4969e-05
    'sex'               0.00019534      0.056396    0.0034638    46    0.99725       -0.11332
    'age'                  0.02382      0.037586      0.63374    46    0.52939      -0.051837
    'drugOrder'           0.039556      0.053777      0.73555    46    0.46574      -0.068692
    'fiveDASC'               0.027      0.021698       1.2444    46    0.21967      -0.016675


    Upper
       0.05121
       0.11817
       0.47116
    1.1121e-05
       0.11372
      0.099477
        0.1478
      0.070674

Random effects covariance parameters (95% CIs):
Group: subject (27 Levels)
    Name1                Name2                Type         Estimate    Lower       Upper
    '(Intercept)'        '(Intercept)'        'std'        0.10006     0.050837    0.19696

Group: Error
    Name             Estimate    Lower       Upper
    'Res Std'        0.12782     0.093471    0.1748


 Contrast: fb success vs fb neutral      ROI: striatum 5D-ASC subscore: asc_vis

lme =


Linear mixed-effects model fit by REML

Model information:
    Number of observations             54
    Fixed effects coefficients          8
    Random effects coefficients        27
    Covariance parameters               2

Formula:
    ROI ~ 1 + measurement + drug + ketLvl + sex + age + drugOrder + fiveDASC + (1 | subject)

Model fit statistics:
    AIC       BIC     LogLikelihood    Deviance
    20.414    38.7    -0.20681         0.41361

Fixed effects coefficients (95% CIs):
    Name                 Estimate       SE            tStat        DF    pValue     Lower
    '(Intercept)'          -0.17558       0.10709      -1.6395    46    0.10793      -0.39115
    'measurement'          0.048396      0.039531       1.2243    46    0.22709     -0.031176
    'drug'                 0.095095       0.19052      0.49912    46    0.62007      -0.28841
    'ketLvl'            -6.9238e-06    9.1296e-06     -0.75838    46    0.45209    -2.5301e-05
    'sex'                -0.0010857      0.054074    -0.020078    46    0.98407      -0.10993
    'age'                  0.026638      0.035759      0.74493    46     0.4601     -0.045341
    'drugOrder'            0.043569      0.051155      0.85171    46    0.39879       -0.0594
    'fiveDASC'             0.018742      0.022185      0.84483    46    0.40258     -0.025913


    Upper
       0.03999
       0.12797
        0.4786
    1.1453e-05
       0.10776
      0.098616
       0.14654
      0.063397

Random effects covariance parameters (95% CIs):
Group: subject (27 Levels)
    Name1                Name2                Type         Estimate    Lower       Upper
    '(Intercept)'        '(Intercept)'        'std'        0.086132    0.039687    0.18693

Group: Error
    Name             Estimate    Lower      Upper
    'Res Std'        0.13493     0.10073    0.18075
```

```
 Contrast: fb success vs fb neutral      ROI: striatum 5D-ASC subscore: asc_aud

lme = 


Linear mixed-effects model fit by REML

Model information:
    Number of observations              54
    Fixed effects coefficients           8
    Random effects coefficients         27
    Covariance parameters                2

Formula:
    ROI ~ 1 + measurement + drug + ketLvl + sex + age + drugOrder + fiveDASC + (1 | subject)

Model fit statistics:
    AIC       BIC      LogLikelihood    Deviance
    20.854    39.14    -0.42681         0.85362 

Fixed effects coefficients (95% CIs):
    Name                   Estimate       SE            tStat       DF    pValue     Lower      
    '(Intercept)'             -0.15982       0.10688     -1.4953    46    0.14166       -0.37495
    'measurement'              0.03189      0.038887     0.82005    46    0.41641      -0.046386
    'drug'                    0.091674        0.1947     0.47086    46    0.63997       -0.30023
    'ketLvl'               -6.7867e-06    9.3259e-06    -0.72773    46    0.47047    -2.5559e-05
    'sex'                    0.0018303      0.053086    0.034478    46    0.97265       -0.10503
    'age'                      0.02823      0.035014     0.80626    46    0.42424      -0.042249
    'drugOrder'               0.049114      0.049706     0.98809    46    0.32828      -0.050939
    'fiveDASC'               -0.011371      0.022074    -0.51514    46    0.60892      -0.055804


    Upper     
      0.055316
       0.11017
       0.48358
    1.1985e-05
       0.10869
      0.098709
       0.14917
      0.033062

Random effects covariance parameters (95% CIs):
Group: subject (27 Levels)
    Name1                Name2                Type         Estimate    Lower      Upper 
    '(Intercept)'        '(Intercept)'        'std'        0.079223    0.03257    0.1927

Group: Error
    Name             Estimate    Lower      Upper  
    'Res Std'        0.13864     0.10385    0.18509


 Contrast: fb success vs fb neutral      ROI: striatum 5D-ASC subscore: asc_vig

lme = 


Linear mixed-effects model fit by REML

Model information:
    Number of observations              54
    Fixed effects coefficients           8
    Random effects coefficients         27
    Covariance parameters                2

Formula:
    ROI ~ 1 + measurement + drug + ketLvl + sex + age + drugOrder + fiveDASC + (1 | subject)

Model fit statistics:
    AIC       BIC      LogLikelihood    Deviance
    21.024    39.31    -0.51185         1.0237  

Fixed effects coefficients (95% CIs):
    Name                   Estimate       SE            tStat       DF    pValue     Lower      
    '(Intercept)'             -0.16579       0.10631     -1.5594    46    0.12575       -0.37979
    'measurement'             0.033929      0.039983     0.84858    46    0.40051      -0.046553
    'drug'                      0.1067       0.19374     0.55073    46    0.58448       -0.28329
```

```
    'ketLvl'            -7.5163e-06    9.2794e-06       -0.81    46    0.42211    -2.6195e-05
    'sex'                 0.0022702      0.053102    0.042752    46    0.96608       -0.10462
    'age'                   0.02928       0.03495     0.83777    46    0.40649      -0.041071
    'drugOrder'            0.051056      0.050546      1.0101    46    0.31773      -0.050687
    'fiveDASC'           -0.0043637      0.022813    -0.19128    46    0.84914      -0.050283


    Upper
      0.048209
       0.11441
       0.49669
    1.1162e-05
       0.10916
      0.099631
        0.1528
      0.041556

Random effects covariance parameters (95% CIs):
Group: subject (27 Levels)
    Name1                Name2                Type         Estimate    Lower       Upper
    '(Intercept)'        '(Intercept)'        'std'        0.078654    0.030019    0.20609

Group: Error
    Name             Estimate    Lower      Upper
    'Res Std'        0.13931     0.10332    0.18783


 Contrast: fb success vs fb neutral      ROI: striatum 5D-ASC subscore: sum

lme =


Linear mixed-effects model fit by REML

Model information:
    Number of observations             54
    Fixed effects coefficients          8
    Random effects coefficients        27
    Covariance parameters               2

Formula:
    ROI ~ 1 + measurement + drug + ketLvl + sex + age + drugOrder + fiveDASC + (1 | subject)

Model fit statistics:
    AIC       BIC       LogLikelihood    Deviance
    20.404    38.691    -0.20224         0.40449

Fixed effects coefficients (95% CIs):
    Name                 Estimate       SE            tStat        DF    pValue     Lower
    '(Intercept)'          -0.17446       0.10787     -1.6173     46    0.11266       -0.39159
    'measurement'          0.047846      0.038421      1.2453     46    0.21933      -0.029491
    'drug'                 0.095648       0.18969     0.50423     46    0.61651       -0.28618
    'ketLvl'            -6.9518e-06    9.0931e-06    -0.76451     46    0.44847    -2.5255e-05
    'sex'                -0.0010143      0.055029   -0.018431     46    0.98537       -0.11178
    'age'                  0.026653      0.036453     0.73116     46    0.46839      -0.046723
    'drugOrder'            0.043354      0.052181     0.83085     46    0.41035       -0.06168
    'fiveDASC'               0.0199      0.022608     0.88023     46    0.38331      -0.025607


    Upper
      0.042676
       0.12518
       0.47748
    1.1352e-05
       0.10975
       0.10003
       0.14839
      0.065407

Random effects covariance parameters (95% CIs):
Group: subject (27 Levels)
    Name1                Name2                Type         Estimate    Lower       Upper
    '(Intercept)'        '(Intercept)'        'std'        0.091777    0.043782    0.19239

Group: Error
    Name             Estimate    Lower       Upper
    'Res Std'        0.13249     0.097993    0.17914
```

```
 Contrast: fb success vs fb neutral      ROI: nucleus accumbens 5D-ASC subscore: asc_obn

lme =


Linear mixed-effects model fit by REML

Model information:
    Number of observations              54
    Fixed effects coefficients           8
    Random effects coefficients         27
    Covariance parameters                2

Formula:
    ROI ~ 1 + measurement + drug + ketLvl + sex + age + drugOrder + fiveDASC + (1 | subject)

Model fit statistics:
    AIC       BIC      LogLikelihood    Deviance
    36.384    54.67    -8.192           16.384

Fixed effects coefficients (95% CIs):
    Name                    Estimate       SE            tStat       DF    pValue     Lower
    '(Intercept)'           -0.15625       0.12174     -1.2835     46    0.20574       -0.40129
    'measurement'           0.074065      0.051885      1.4275     46    0.16019      -0.030374
    'drug'                   0.16336       0.23701     0.68923     46    0.49414       -0.31372
    'ketLvl'             -5.7099e-06    1.1308e-05    -0.50496     46      0.616    -2.8471e-05
    'sex'                   0.049241      0.055976     0.87968     46     0.3836      -0.063432
    'age'                    0.04454      0.036583      1.2175     46    0.22962      -0.029097
    'drugOrder'           -0.0096367      0.051878    -0.18576     46    0.85345       -0.11406
    'fiveDASC'              0.007953      0.026411     0.30113     46    0.76468      -0.045209


    Upper
       0.08879
        0.1785
       0.64043
    1.7051e-05
       0.16191
       0.11818
      0.094788
      0.061115

Random effects covariance parameters (95% CIs):
Group: subject (27 Levels)
    Name1                Name2                Type         Estimate      Lower
    '(Intercept)'        '(Intercept)'        'std'        0.00012964    5.113e-272


    Upper
    3.2871e+263

Group: Error
    Name             Estimate    Lower      Upper
    'Res Std'        0.18529     0.15105    0.2273


 Contrast: fb success vs fb neutral      ROI: nucleus accumbens 5D-ASC subscore: asc_aed

lme =


Linear mixed-effects model fit by REML

Model information:
    Number of observations              54
    Fixed effects coefficients           8
    Random effects coefficients         27
    Covariance parameters                2

Formula:
    ROI ~ 1 + measurement + drug + ketLvl + sex + age + drugOrder + fiveDASC + (1 | subject)

Model fit statistics:
    AIC       BIC       LogLikelihood    Deviance
    36.189    54.475    -8.0943          16.189

Fixed effects coefficients (95% CIs):
```

Name	Estimate	SE	tStat	DF	pValue	Lower
'(Intercept)'	-0.15308	0.12113	-1.2638	46	0.21267	-0.3969
'measurement'	0.0662	0.051359	1.289	46	0.20385	-0.03718
'drug'	0.13735	0.23839	0.57616	46	0.56731	-0.3425
'ketLvl'	-4.455e-06	1.1377e-05	-0.39159	46	0.69717	-2.7355e-05
'sex'	0.049144	0.055843	0.88003	46	0.38342	-0.063263
'age'	0.048353	0.036619	1.3204	46	0.19322	-0.025357
'drugOrder'	-0.0031671	0.052537	-0.060283	46	0.95219	-0.10892
'fiveDASC'	-0.013604	0.02673	-0.50892	46	0.61324	-0.067409

Upper
0.090735
0.16958
0.6172
1.8445e-05
0.16155
0.12206
0.10259
0.040202

Random effects covariance parameters (95% CIs):
Group: subject (27 Levels)

Name1	Name2	Type	Estimate	Lower
'(Intercept)'	'(Intercept)'	'std'	0.00011161	4.1913e-276

Upper
2.9721e+267

Group: Error

Name	Estimate	Lower	Upper
'Res Std'	0.18495	0.15077	0.22688

Contrast: fb success vs fb neutral   ROI: nucleus accumbens 5D-ASC subscore: asc_vis

lme =

Linear mixed-effects model fit by REML

Model information:

Number of observations	54
Fixed effects coefficients	8
Random effects coefficients	27
Covariance parameters	2

Formula:
ROI ~ 1 + measurement + drug + ketLvl + sex + age + drugOrder + fiveDASC + (1 | subject)

Model fit statistics:

AIC	BIC	LogLikelihood	Deviance
36.317	54.603	-8.1585	16.317

Fixed effects coefficients (95% CIs):

Name	Estimate	SE	tStat	DF	pValue	Lower
'(Intercept)'	-0.14932	0.12202	-1.2237	46	0.22729	-0.39493
'measurement'	0.06535	0.053522	1.221	46	0.22832	-0.042385
'drug'	0.15434	0.23595	0.65414	46	0.51628	-0.32059
'ketLvl'	-5.2858e-06	1.1256e-05	-0.46961	46	0.64085	-2.7942e-05
'sex'	0.050952	0.056002	0.90984	46	0.36765	-0.061773
'age'	0.047	0.036541	1.2862	46	0.2048	-0.026553
'drugOrder'	-0.0056069	0.052362	-0.10708	46	0.91519	-0.11101
'fiveDASC'	-0.0084811	0.02722	-0.31157	46	0.75677	-0.063272

Upper
0.096294
0.17308
0.62928
1.7371e-05
0.16368
0.12055
0.099792
0.04631

Random effects covariance parameters (95% CIs):

```
Group: subject (27 Levels)
    Name1                Name2                Type         Estimate      Lower      
    '(Intercept)'        '(Intercept)'        'std'        0.00012171    5.6382e-261


    Upper      
    2.6274e+252

Group: Error
    Name             Estimate    Lower      Upper  
    'Res Std'        0.18528     0.15103    0.22728


 Contrast: fb success vs fb neutral      ROI: nucleus accumbens 5D-ASC subscore: asc_aud

lme = 


Linear mixed-effects model fit by REML

Model information:
    Number of observations              54
    Fixed effects coefficients           8
    Random effects coefficients         27
    Covariance parameters                2

Formula:
    ROI ~ 1 + measurement + drug + ketLvl + sex + age + drugOrder + fiveDASC + (1 | subject)

Model fit statistics:
    AIC      BIC       LogLikelihood    Deviance
    28.43    46.716    -4.215           8.4301  

Fixed effects coefficients (95% CIs):
    Name                 Estimate       SE           tStat       DF    pValue      Lower      
    '(Intercept)'           -0.11088      0.11217     -0.9885    46     0.32808       -0.33667
    'measurement'           0.043431     0.047364     0.91696    46     0.36395      -0.051908
    'drug'                  0.080724      0.21776     0.37071    46     0.71255        -0.3576
    'ketLvl'             -1.7327e-06    1.039e-05    -0.16677    46     0.86828    -2.2646e-05
    'sex'                   0.048683     0.051273     0.94948    46     0.34734      -0.054525
    'age'                   0.038271     0.033416      1.1453    46     0.25801      -0.028992
    'drugOrder'           -0.0095267     0.047379    -0.20107    46     0.84153        -0.1049
    'fiveDASC'             -0.070827     0.023838     -2.9712    46    0.004704       -0.11881


    Upper     
       0.11491
       0.13877
       0.51904
    1.9181e-05
       0.15189
       0.10553
      0.085843
     -0.022844

Random effects covariance parameters (95% CIs):
Group: subject (27 Levels)
    Name1                Name2                Type         Estimate      Lower      
    '(Intercept)'        '(Intercept)'        'std'        0.00010544    2.6054e-258


    Upper      
    4.2673e+249

Group: Error
    Name             Estimate    Lower      Upper 
    'Res Std'        0.16989     0.13849    0.2084


 Contrast: fb success vs fb neutral      ROI: nucleus accumbens 5D-ASC subscore: asc_vig

lme = 


Linear mixed-effects model fit by REML

Model information:
    Number of observations              54
```

```
    Fixed effects coefficients           8
    Random effects coefficients         27
    Covariance parameters                2

Formula:
    ROI ~ 1 + measurement + drug + ketLvl + sex + age + drugOrder + fiveDASC + (1 | subject)

Model fit statistics:
    AIC       BIC       LogLikelihood    Deviance
    36.021    54.308    -8.0107          16.021

Fixed effects coefficients (95% CIs):
    Name                 Estimate       SE            tStat        DF    pValue     Lower
    '(Intercept)'           -0.15006         0.121      -1.2401    46    0.22121       -0.39362
    'measurement'           0.061611      0.052357       1.1767    46    0.24535      -0.043779
    'drug'                   0.15385       0.23503       0.6546    46    0.51599       -0.31925
    'ketLvl'             -5.2686e-06    1.1213e-05     -0.46989    46    0.64066    -2.7838e-05
    'sex'                   0.049847      0.055715      0.89468    46    0.37562      -0.062301
    'age'                   0.044996      0.036228        1.242    46    0.22052      -0.027927
    'drugOrder'          -0.0011854      0.052593    -0.022539    46    0.98212       -0.10705
    'fiveDASC'             -0.017497      0.026618     -0.65733    46    0.51424      -0.071076


    Upper
      0.093505
         0.167
       0.62695
    1.7301e-05
       0.16199
       0.11792
       0.10468
      0.036082

Random effects covariance parameters (95% CIs):
Group: subject (27 Levels)
    Name1                Name2                Type         Estimate      Lower
    '(Intercept)'        '(Intercept)'        'std'        0.00010502    2.4407e-280


    Upper
    4.5185e+271

Group: Error
    Name             Estimate    Lower      Upper
    'Res Std'        0.18461     0.15049    0.22646


 Contrast: fb success vs fb neutral      ROI: nucleus accumbens 5D-ASC subscore: sum

lme =


Linear mixed-effects model fit by REML

Model information:
    Number of observations              54
    Fixed effects coefficients           8
    Random effects coefficients         27
    Covariance parameters                2

Formula:
    ROI ~ 1 + measurement + drug + ketLvl + sex + age + drugOrder + fiveDASC + (1 | subject)

Model fit statistics:
    AIC       BIC       LogLikelihood    Deviance
    36.251    54.538    -8.1257          16.251

Fixed effects coefficients (95% CIs):
    Name                 Estimate       SE            tStat        DF    pValue     Lower
    '(Intercept)'           -0.14895       0.12167      -1.2242    46    0.22712       -0.39387
    'measurement'           0.064399      0.052796       1.2198    46    0.22877      -0.041875
    'drug'                   0.14573       0.23699      0.61492    46    0.54164       -0.33131
    'ketLvl'             -4.8672e-06    1.1306e-05     -0.43049    46    0.66885    -2.7626e-05
    'sex'                   0.050579      0.055885      0.90506    46    0.37015      -0.061911
    'age'                   0.047215       0.03646        1.295    46    0.20179      -0.026176
    'drugOrder'          -0.0047286      0.052294    -0.090423    46    0.92834       -0.10999
    'fiveDASC'             -0.011393       0.02699     -0.42211    46    0.67491      -0.065722
```

```
    Upper    
      0.095966
       0.17067
       0.62277
    1.7891e-05
       0.16307
       0.12061
       0.10053
      0.042936

Random effects covariance parameters (95% CIs):
Group: subject (27 Levels)
    Name1                    Name2                    Type           Estimate      Lower     
    '(Intercept)'        '(Intercept)'        'std'          0.00011313    9.511e-270


    Upper     
    1.3456e+261

Group: Error
    Name               Estimate    Lower     Upper  
    'Res Std'          0.18511     0.1509    0.22708


 Contrast: fb success vs fb neutral      ROI: insula 5D-ASC subscore: asc_obn

lme = 


Linear mixed-effects model fit by REML

Model information:
    Number of observations              54
    Fixed effects coefficients           8
    Random effects coefficients         27
    Covariance parameters                2

Formula:
    ROI ~ 1 + measurement + drug + ketLvl + sex + age + drugOrder + fiveDASC + (1 | subject)

Model fit statistics:
    AIC       BIC       LogLikelihood    Deviance
    88.585    106.87    -34.293          68.585  

Fixed effects coefficients (95% CIs):
    Name                    Estimate       SE            tStat        DF    pValue      Lower      
    '(Intercept)'            -0.40469         0.2333     -1.7347      46    0.089499       -0.87429
    'measurement'             0.11002       0.073853      1.4898      46     0.14311      -0.038635
    'drug'                     0.0266        0.38558    0.068987      46      0.9453       -0.74954
    'ketLvl'              -3.0762e-06     1.8506e-05    -0.16623      46      0.8687    -4.0326e-05
    'sex'                    0.049079        0.12254     0.40052      46     0.69063       -0.19758
    'age'                    0.067078       0.081643      0.8216      46     0.41554      -0.097261
    'drugOrder'               0.19224        0.11603      1.6568      46     0.10437      -0.041321
    'fiveDASC'               0.038734       0.044167     0.87698      46     0.38505       -0.05017


    Upper    
      0.064912
       0.25868
       0.80274
    3.4174e-05
       0.29573
       0.23142
       0.42581
       0.12764

Random effects covariance parameters (95% CIs):
Group: subject (27 Levels)
    Name1                    Name2                    Type           Estimate    Lower      Upper  
    '(Intercept)'        '(Intercept)'        'std'          0.22849     0.13691    0.38134

Group: Error
    Name               Estimate    Lower      Upper  
    'Res Std'          0.26082     0.19628    0.34658


 Contrast: fb success vs fb neutral      ROI: insula 5D-ASC subscore: asc_aed
```

```
lme = 


Linear mixed-effects model fit by REML

Model information:
    Number of observations              54
    Fixed effects coefficients           8
    Random effects coefficients         27
    Covariance parameters                2

Formula:
    ROI ~ 1 + measurement + drug + ketLvl + sex + age + drugOrder + fiveDASC + (1 | subject)

Model fit statistics:
    AIC       BIC       LogLikelihood    Deviance
    88.837    107.12    -34.418          68.837  

Fixed effects coefficients (95% CIs):
    Name                    Estimate       SE            tStat       DF    pValue     Lower    
    '(Intercept)'             -0.39107       0.23431      -1.669    46    0.10191      -0.86272
    'measurement'              0.10429      0.072852      1.4316    46    0.15902     -0.042349
    'drug'                     0.02721       0.38679    0.070347    46    0.94422      -0.75136
    'ketLvl'               -3.1163e-06    1.8567e-05    -0.16784    46    0.86745    -4.049e-05
    'sex'                     0.053985       0.12361     0.43674    46    0.66435      -0.19483
    'age'                     0.067252      0.082595     0.81423    46    0.41971     -0.099004
    'drugOrder'                0.18735       0.11812      1.5861    46    0.11958     -0.050419
    'fiveDASC'                 0.03133      0.045162     0.69372    46    0.49134     -0.059577


    Upper     
      0.080583
       0.25094
       0.80578
    3.4258e-05
        0.3028
       0.23351
       0.42512
       0.12224

Random effects covariance parameters (95% CIs):
Group: subject (27 Levels)
    Name1                   Name2                   Type         Estimate    Lower      Upper  
    '(Intercept)'           '(Intercept)'           'std'        0.23268     0.14022    0.38612

Group: Error
    Name                Estimate    Lower      Upper  
    'Res Std'           0.26        0.19539    0.34596


 Contrast: fb success vs fb neutral      ROI: insula 5D-ASC subscore: asc_vis

lme = 


Linear mixed-effects model fit by REML

Model information:
    Number of observations              54
    Fixed effects coefficients           8
    Random effects coefficients         27
    Covariance parameters                2

Formula:
    ROI ~ 1 + measurement + drug + ketLvl + sex + age + drugOrder + fiveDASC + (1 | subject)

Model fit statistics:
    AIC       BIC       LogLikelihood    Deviance
    87.763    106.05    -33.881          67.763  

Fixed effects coefficients (95% CIs):
    Name                    Estimate       SE           tStat        DF    pValue     Lower      
    '(Intercept)'              -0.4169      0.23208      -1.7963    46    0.07901       -0.88405
    'measurement'              0.12915     0.076052       1.6982    46    0.09622      -0.023931
    'drug'                    0.003565      0.38025    0.0093755    46    0.99256       -0.76184
    'ketLvl'               -1.9175e-06    1.825e-05     -0.10506    46    0.91678    -3.8654e-05
    'sex'                     0.046203      0.12188       0.3791    46    0.70636       -0.19912
```

```
    'age'                   0.065317      0.08114      0.80499    46    0.42497      -0.098009
    'drugOrder'              0.18157      0.11598       1.5656    46    0.12431      -0.051882
    'fiveDASC'              0.055942     0.044452       1.2585    46    0.21457      -0.033536


    Upper
       0.05026
       0.28224
       0.76897
    3.4819e-05
       0.29153
       0.22864
       0.41502
       0.14542

Random effects covariance parameters (95% CIs):
Group: subject (27 Levels)
    Name1                Name2                Type         Estimate    Lower      Upper
    '(Intercept)'        '(Intercept)'        'std'        0.22815     0.13737    0.37893

Group: Error
    Name             Estimate    Lower      Upper
    'Res Std'        0.25781     0.19397    0.34267


 Contrast: fb success vs fb neutral      ROI: insula 5D-ASC subscore: asc_aud

lme =


Linear mixed-effects model fit by REML

Model information:
    Number of observations              54
    Fixed effects coefficients           8
    Random effects coefficients         27
    Covariance parameters                2

Formula:
    ROI ~ 1 + measurement + drug + ketLvl + sex + age + drugOrder + fiveDASC + (1 | subject)

Model fit statistics:
    AIC       BIC       LogLikelihood    Deviance
    88.401    106.69    -34.2            68.401

Fixed effects coefficients (95% CIs):
    Name                 Estimate       SE            tStat       DF    pValue      Lower
    '(Intercept)'           -0.41619       0.23422     -1.7769    46     0.08219       -0.88765
    'measurement'            0.11012      0.073231      1.5037    46     0.13949      -0.037288
    'drug'                  0.047857       0.38682     0.12372    46     0.90208       -0.73076
    'ketLvl'             -4.1134e-06    1.8568e-05    -0.22153    46     0.82566    -4.1489e-05
    'sex'                   0.054226       0.12254     0.44253    46     0.66018       -0.19243
    'age'                   0.077927      0.081547     0.95561    46     0.34426      -0.086218
    'drugOrder'              0.19971       0.11588      1.7234    46    0.091533      -0.033547
    'fiveDASC'              0.043085      0.045028     0.95684    46     0.34365      -0.047552


    Upper
      0.055264
       0.25753
       0.82648
    3.3262e-05
       0.30088
       0.24207
       0.43296
       0.13372

Random effects covariance parameters (95% CIs):
Group: subject (27 Levels)
    Name1                Name2                Type         Estimate    Lower      Upper
    '(Intercept)'        '(Intercept)'        'std'        0.22936     0.13794    0.38138

Group: Error
    Name             Estimate    Lower      Upper
    'Res Std'        0.25984     0.19566    0.34506


 Contrast: fb success vs fb neutral      ROI: insula 5D-ASC subscore: asc_vig
```

```
lme = 


Linear mixed-effects model fit by REML

Model information:
    Number of observations              54
    Fixed effects coefficients           8
    Random effects coefficients         27
    Covariance parameters                2

Formula:
    ROI ~ 1 + measurement + drug + ketLvl + sex + age + drugOrder + fiveDASC + (1 | subject)

Model fit statistics:
    AIC       BIC       LogLikelihood    Deviance
    88.792    107.08    -34.396          68.792  

Fixed effects coefficients (95% CIs):
    Name                    Estimate       SE            tStat       DF    pValue      Lower      
    '(Intercept)'             -0.38441       0.23272     -1.6519    46     0.10537       -0.85285
    'measurement'             0.076896      0.076415      1.0063    46     0.31954       -0.07692
    'drug'                     0.01188       0.38745    0.030662    46     0.97567       -0.76802
    'ketLvl'               -2.4177e-06    1.8597e-05    -0.13001    46     0.89713    -3.9851e-05
    'sex'                     0.054275       0.12199     0.44493    46     0.65846       -0.19127
    'age'                     0.072071      0.080992     0.88986    46     0.37817      -0.090956
    'drugOrder'                 0.2115       0.11678      1.8112    46    0.076649      -0.023558
    'fiveDASC'               -0.031385       0.04693    -0.66876    46     0.50699       -0.12585


    Upper     
      0.084017
       0.23071
       0.79178
    3.5015e-05
       0.29982
        0.2351
       0.44656
       0.06308

Random effects covariance parameters (95% CIs):
Group: subject (27 Levels)
    Name1                   Name2                   Type         Estimate    Lower      Upper  
    '(Intercept)'           '(Intercept)'           'std'        0.2251      0.13307    0.38078

Group: Error
    Name               Estimate    Lower     Upper  
    'Res Std'          0.26372     0.1986    0.35019


 Contrast: fb success vs fb neutral      ROI: insula 5D-ASC subscore: sum

lme = 


Linear mixed-effects model fit by REML

Model information:
    Number of observations              54
    Fixed effects coefficients           8
    Random effects coefficients         27
    Covariance parameters                2

Formula:
    ROI ~ 1 + measurement + drug + ketLvl + sex + age + drugOrder + fiveDASC + (1 | subject)

Model fit statistics:
    AIC       BIC       LogLikelihood    Deviance
    88.435    106.72    -34.218          68.435  

Fixed effects coefficients (95% CIs):
    Name                    Estimate       SE            tStat       DF    pValue     Lower      
    '(Intercept)'             -0.40673       0.23431     -1.7359    46    0.08928      -0.87837
    'measurement'              0.11735      0.075516       1.554    46    0.12703     -0.034651
    'drug'                    0.020219       0.38337    0.052739    46    0.95817      -0.75147
    'ketLvl'               -2.7515e-06    1.8401e-05    -0.14953    46    0.88179    -3.979e-05
    'sex'                     0.049656       0.12334     0.40258    46    0.68912      -0.19862
```

```
    'age'                  0.067863      0.082159     0.82599    46    0.41307     -0.097515
    'drugOrder'             0.18603       0.11752       1.583    46    0.12028     -0.050526
    'fiveDASC'             0.042214      0.046456      0.9087    46    0.36825     -0.051296


    Upper
      0.064907
       0.26936
       0.79191
    3.4287e-05
       0.29794
       0.23324
       0.42258
       0.13572

Random effects covariance parameters (95% CIs):
Group: subject (27 Levels)
    Name1                Name2                Type         Estimate    Lower      Upper
    '(Intercept)'        '(Intercept)'        'std'        0.23249     0.14062    0.38438

Group: Error
    Name             Estimate    Lower      Upper
    'Res Std'        0.25873     0.19463    0.34394


 Contrast: fb success vs fb neutral      ROI: thalamus 5D-ASC subscore: asc_obn

lme =


Linear mixed-effects model fit by REML

Model information:
    Number of observations              54
    Fixed effects coefficients           8
    Random effects coefficients         27
    Covariance parameters                2

Formula:
    ROI ~ 1 + measurement + drug + ketLvl + sex + age + drugOrder + fiveDASC + (1 | subject)

Model fit statistics:
    AIC        BIC       LogLikelihood    Deviance
    -1.6037    16.683    10.802           -21.604

Fixed effects coefficients (95% CIs):
    Name                 Estimate      SE            tStat      DF    pValue     Lower
    '(Intercept)'          0.043807      0.081379     0.5383    46    0.59296          -0.12
    'measurement'          0.013387      0.032788    0.40828    46    0.68497      -0.052613
    'drug'                 -0.16673       0.15535    -1.0733    46    0.28876       -0.47945
    'ketLvl'             6.4834e-06    7.4252e-06    0.87316    46    0.38711    -8.4627e-06
    'sex'                 -0.059154      0.038819    -1.5238    46     0.1344       -0.13729
    'age'                  0.023809      0.025487    0.93417    46     0.3551      -0.027493
    'drugOrder'            0.027151      0.036161    0.75082    46    0.45658      -0.045638
    'fiveDASC'             0.022126      0.017432     1.2692    46    0.21074      -0.012964


    Upper
       0.20762
      0.079386
       0.14598
    2.1429e-05
      0.018985
      0.075111
      0.099939
      0.057215

Random effects covariance parameters (95% CIs):
Group: subject (27 Levels)
    Name1                Name2                Type         Estimate    Lower        Upper
    '(Intercept)'        '(Intercept)'        'std'        0.039105    0.0051287    0.29816

Group: Error
    Name             Estimate    Lower       Upper
    'Res Std'        0.11678     0.087996    0.15499


 Contrast: fb success vs fb neutral      ROI: thalamus 5D-ASC subscore: asc_aed
```

```
lme =


Linear mixed-effects model fit by REML

Model information:
    Number of observations              54
    Fixed effects coefficients           8
    Random effects coefficients         27
    Covariance parameters                2

Formula:
    ROI ~ 1 + measurement + drug + ketLvl + sex + age + drugOrder + fiveDASC + (1 | subject)

Model fit statistics:
    AIC          BIC       LogLikelihood    Deviance
    -0.052621    18.234    10.026           -20.053

Fixed effects coefficients (95% CIs):
    Name                 Estimate       SE            tStat        DF    pValue     Lower
    '(Intercept)'           0.051962      0.082597      0.62911    46    0.53239        -0.1143
    'measurement'             0.0039      0.032968       0.1183    46    0.90635       -0.06246
    'drug'                  -0.18536       0.15896       -1.166    46    0.24962       -0.50533
    'ketLvl'              7.3749e-06    7.6002e-06      0.97036    46    0.33694    -7.9234e-06
    'sex'                  -0.056988      0.039578      -1.4399    46    0.15666       -0.13665
    'age'                   0.027421      0.026076       1.0516    46     0.2985      -0.025068
    'drugOrder'             0.031154      0.037381      0.83341    46    0.40892      -0.044091
    'fiveDASC'           -0.00042103      0.018009    -0.023379    46    0.98145      -0.036671


    Upper
       0.21822
       0.07026
       0.13462
    2.2673e-05
      0.022678
      0.079909
        0.1064
      0.035829

Random effects covariance parameters (95% CIs):
Group: subject (27 Levels)
    Name1                Name2                Type         Estimate    Lower        Upper
    '(Intercept)'        '(Intercept)'        'std'        0.040954    0.0058998    0.28429

Group: Error
    Name             Estimate    Lower      Upper
    'Res Std'        0.11847     0.08916    0.15741


 Contrast: fb success vs fb neutral      ROI: thalamus 5D-ASC subscore: asc_vis

lme =


Linear mixed-effects model fit by REML

Model information:
    Number of observations              54
    Fixed effects coefficients           8
    Random effects coefficients         27
    Covariance parameters                2

Formula:
    ROI ~ 1 + measurement + drug + ketLvl + sex + age + drugOrder + fiveDASC + (1 | subject)

Model fit statistics:
    AIC         BIC    LogLikelihood    Deviance
    -0.28671    18     10.143           -20.287

Fixed effects coefficients (95% CIs):
    Name                 Estimate      SE            tStat      DF    pValue     Lower
    '(Intercept)'          0.047982      0.082786    0.57959    46    0.56502       -0.11866
    'measurement'         0.0093699      0.034424    0.27219    46    0.78669      -0.059922
    'drug'                 -0.18383       0.15703    -1.1707    46    0.24774       -0.49991
    'ketLvl'             7.3105e-06    7.5044e-06    0.97416    46    0.33507    -7.7951e-06
    'sex'                 -0.057995       0.03941    -1.4716    46    0.14795       -0.13732
```

```
    'age'                 0.026146      0.025836      1.012    46    0.31684       -0.02586
    'drugOrder'           0.028389         0.037    0.76726    46    0.44685      -0.046089
    'fiveDASC'           0.0083898      0.018179    0.46151    46    0.64661      -0.028203


    Upper
       0.21462
      0.078662
       0.13224
    2.2416e-05
      0.021334
      0.078151
       0.10287
      0.044982

Random effects covariance parameters (95% CIs):
Group: subject (27 Levels)
    Name1                Name2                Type         Estimate    Lower        Upper
    '(Intercept)'        '(Intercept)'        'std'        0.039581    0.0051216    0.30589

Group: Error
    Name             Estimate    Lower       Upper
    'Res Std'        0.11857     0.089308    0.15742


 Contrast: fb success vs fb neutral      ROI: thalamus 5D-ASC subscore: asc_aud

lme =


Linear mixed-effects model fit by REML

Model information:
    Number of observations              54
    Fixed effects coefficients           8
    Random effects coefficients         27
    Covariance parameters                2

Formula:
    ROI ~ 1 + measurement + drug + ketLvl + sex + age + drugOrder + fiveDASC + (1 | subject)

Model fit statistics:
    AIC          BIC       LogLikelihood    Deviance
    -0.050313    18.236    10.025           -20.05

Fixed effects coefficients (95% CIs):
    Name                 Estimate      SE            tStat       DF    pValue     Lower
    '(Intercept)'          0.054168      0.08317      0.65129    46     0.5181       -0.11324
    'measurement'         0.0026304     0.033184     0.079269    46    0.93716      -0.064165
    'drug'                 -0.18957      0.15843      -1.1965    46    0.23762       -0.50847
    'ketLvl'             7.5783e-06    7.573e-06       1.0007    46    0.32221    -7.6654e-06
    'sex'                 -0.057095     0.039446      -1.4474    46    0.15456        -0.1365
    'age'                  0.026938     0.025836       1.0426    46    0.30257      -0.025068
    'drugOrder'            0.030936     0.036651      0.84407    46      0.403      -0.042839
    'fiveDASC'           -0.0037029     0.017599      -0.2104    46    0.83429      -0.039129


    Upper
       0.22158
      0.069426
       0.12934
    2.2822e-05
      0.022306
      0.078944
       0.10471
      0.031723

Random effects covariance parameters (95% CIs):
Group: subject (27 Levels)
    Name1                Name2                Type         Estimate    Lower        Upper
    '(Intercept)'        '(Intercept)'        'std'        0.039847    0.0050963    0.31156

Group: Error
    Name             Estimate    Lower       Upper
    'Res Std'        0.11873     0.089311    0.15784


 Contrast: fb success vs fb neutral      ROI: thalamus 5D-ASC subscore: asc_vig
```

```
lme = 


Linear mixed-effects model fit by REML

Model information:
    Number of observations              54
    Fixed effects coefficients           8
    Random effects coefficients         27
    Covariance parameters                2

Formula:
    ROI ~ 1 + measurement + drug + ketLvl + sex + age + drugOrder + fiveDASC + (1 | subject)

Model fit statistics:
    AIC          BIC       LogLikelihood    Deviance
    -0.071466    18.215    10.036           -20.071 

Fixed effects coefficients (95% CIs):
    Name                   Estimate     SE            tStat       DF    pValue     Lower      
    '(Intercept)'           0.051665      0.082672     0.62494    46     0.5351       -0.11474
    'measurement'          0.0048472      0.033753     0.14361    46    0.88644      -0.063095
    'drug'                  -0.18467       0.15719     -1.1748    46    0.24611       -0.50107
    'ketLvl'               7.343e-06    7.5134e-06     0.97731    46    0.33353    -7.7808e-06
    'sex'                  -0.056959      0.039583      -1.439    46    0.15693       -0.13664
    'age'                   0.027412       0.02588      1.0592    46    0.29505      -0.024682
    'drugOrder'             0.030373      0.037508     0.80976    46    0.42225      -0.045127
    'fiveDASC'              0.001547      0.018117    0.085392    46    0.93232       -0.03492


    Upper     
       0.21808
      0.072789
       0.13173
    2.2467e-05
      0.022718
      0.079505
       0.10587
      0.038014

Random effects covariance parameters (95% CIs):
Group: subject (27 Levels)
    Name1                Name2                Type         Estimate    Lower        Upper  
    '(Intercept)'        '(Intercept)'        'std'        0.041167    0.0060971    0.27795

Group: Error
    Name             Estimate    Lower       Upper  
    'Res Std'        0.11839     0.089204    0.15714


 Contrast: fb success vs fb neutral      ROI: thalamus 5D-ASC subscore: sum

lme = 


Linear mixed-effects model fit by REML

Model information:
    Number of observations              54
    Fixed effects coefficients           8
    Random effects coefficients         27
    Covariance parameters                2

Formula:
    ROI ~ 1 + measurement + drug + ketLvl + sex + age + drugOrder + fiveDASC + (1 | subject)

Model fit statistics:
    AIC         BIC       LogLikelihood    Deviance
    -0.50724    17.779    10.254           -20.507 

Fixed effects coefficients (95% CIs):
    Name                   Estimate      SE            tStat      DF    pValue     Lower      
    '(Intercept)'            0.047371      0.082465    0.57443    46    0.56847       -0.11862
    'measurement'            0.010676      0.033877    0.31514    46    0.75408      -0.057515
    'drug'                   -0.17517       0.15734    -1.1133    46    0.27136       -0.49188
    'ketLvl'               6.8901e-06    7.5199e-06    0.91625    46    0.36431    -8.2467e-06
    'sex'                   -0.057694      0.039316    -1.4674    46    0.14906       -0.13683
```

```
    'age'                 0.025848      0.025777     1.0028    46    0.32122      -0.026038
    'drugOrder'           0.027324      0.036948    0.73954    46    0.46334      -0.047048
    'fiveDASC'            0.011912      0.018184    0.65511    46    0.51566       -0.02469


    Upper
       0.21336
      0.078867
       0.14154
    2.2027e-05
      0.021445
      0.077733
        0.1017
      0.048515

Random effects covariance parameters (95% CIs):
Group: subject (27 Levels)
    Name1                Name2                Type         Estimate    Lower        Upper
    '(Intercept)'        '(Intercept)'        'std'        0.039911    0.0054001    0.29497

Group: Error
    Name               Estimate    Lower       Upper
    'Res Std'          0.11817     0.089083    0.15676


 Contrast: fb success vs fb neutral      ROI: amygdala 5D-ASC subscore: asc_obn

lme =


Linear mixed-effects model fit by REML

Model information:
    Number of observations              54
    Fixed effects coefficients           8
    Random effects coefficients         27
    Covariance parameters                2

Formula:
    ROI ~ 1 + measurement + drug + ketLvl + sex + age + drugOrder + fiveDASC + (1 | subject)

Model fit statistics:
    AIC     BIC       LogLikelihood    Deviance
    60.9    79.186    -20.45           40.9

Fixed effects coefficients (95% CIs):
    Name                 Estimate      SE             tStat       DF    pValue      Lower
    '(Intercept)'         -0.063556       0.16383    -0.38795    46      0.69984       -0.39332
    'measurement'         -0.040849      0.060861    -0.67119    46      0.50546       -0.16336
    'drug'                 -0.19741       0.30053    -0.65687    46      0.51454       -0.80234
    'ketLvl'             7.1481e-06    1.4391e-05     0.49672    46      0.62175    -2.1819e-05
    'sex'                 -0.074493      0.081435    -0.91476    46      0.36509       -0.23841
    'age'                  0.026329      0.053776      0.4896    46      0.62674      -0.081916
    'drugOrder'             0.15373      0.076349      2.0136    46     0.049925     5.2478e-05
    'fiveDASC'             0.024875      0.034005      0.7315    46      0.46818      -0.043573


    Upper
       0.26621
      0.081657
       0.40752
    3.6115e-05
      0.089427
       0.13457
       0.30742
      0.093322

Random effects covariance parameters (95% CIs):
Group: subject (27 Levels)
    Name1                Name2                Type         Estimate    Lower       Upper
    '(Intercept)'        '(Intercept)'        'std'        0.11798     0.047453    0.29334

Group: Error
    Name               Estimate    Lower      Upper
    'Res Std'          0.21605     0.16273    0.28685


 Contrast: fb success vs fb neutral      ROI: amygdala 5D-ASC subscore: asc_aed
```

```
lme = 


Linear mixed-effects model fit by REML

Model information:
    Number of observations              54
    Fixed effects coefficients           8
    Random effects coefficients         27
    Covariance parameters                2

Formula:
    ROI ~ 1 + measurement + drug + ketLvl + sex + age + drugOrder + fiveDASC + (1 | subject)

Model fit statistics:
    AIC       BIC       LogLikelihood    Deviance
    61.382    79.668    -20.691          41.382  

Fixed effects coefficients (95% CIs):
    Name                     Estimate      SE            tStat        DF    pValue      Lower      
    '(Intercept)'             -0.054344       0.16483     -0.32971    46     0.74312       -0.38612
    'measurement'             -0.052296      0.060108     -0.87002    46     0.38881       -0.17329
    'drug'                     -0.22143       0.30279     -0.73128    46     0.46832       -0.83092
    'ketLvl'                 8.2996e-06    1.4505e-05      0.57221    46     0.56997    -2.0897e-05
    'sex'                     -0.072191      0.082527     -0.87475    46     0.38625       -0.23831
    'age'                       0.03083      0.054701      0.56362    46     0.57575      -0.079276
    'drugOrder'                 0.15911      0.078336       2.0311    46    0.048047      0.0014262
    'fiveDASC'               -0.0028139      0.034747    -0.080982    46     0.93581      -0.072756


    Upper     
       0.27743
      0.068696
       0.38806
    3.7496e-05
      0.093928
       0.14094
       0.31679
      0.067129

Random effects covariance parameters (95% CIs):
Group: subject (27 Levels)
    Name1                    Name2                    Type         Estimate    Lower       Upper  
    '(Intercept)'            '(Intercept)'            'std'        0.12325     0.052965    0.28681

Group: Error
    Name                 Estimate    Lower      Upper  
    'Res Std'            0.21539     0.16243    0.28561


 Contrast: fb success vs fb neutral      ROI: amygdala 5D-ASC subscore: asc_vis

lme = 


Linear mixed-effects model fit by REML

Model information:
    Number of observations              54
    Fixed effects coefficients           8
    Random effects coefficients         27
    Covariance parameters                2

Formula:
    ROI ~ 1 + measurement + drug + ketLvl + sex + age + drugOrder + fiveDASC + (1 | subject)

Model fit statistics:
    AIC       BIC       LogLikelihood    Deviance
    61.276    79.562    -20.638          41.276  

Fixed effects coefficients (95% CIs):
    Name                     Estimate      SE            tStat       DF    pValue      Lower      
    '(Intercept)'              -0.04908       0.16552    -0.29653    46     0.76816       -0.38225
    'measurement'             -0.058477      0.062871    -0.93012    46     0.35716       -0.18503
    'drug'                     -0.22012       0.29965    -0.73459    46     0.46632       -0.82327
    'ketLvl'                 8.2244e-06    1.4351e-05     0.57308    46     0.56938    -2.0663e-05
    'sex'                     -0.070735      0.082579    -0.85657    46     0.39612       -0.23696
```

```
    'age'                 0.031889      0.054506     0.58506    46     0.56136      -0.077825
    'drugOrder'            0.16156      0.077991      2.0715    46    0.043945      0.0045729
    'fiveDASC'           -0.011244      0.034849    -0.32266    46     0.74842      -0.081392


    Upper
       0.28409
      0.068075
       0.38304
    3.7112e-05
      0.095488
        0.1416
       0.31855
      0.058903

Random effects covariance parameters (95% CIs):
Group: subject (27 Levels)
    Name1                Name2                Type         Estimate    Lower       Upper
    '(Intercept)'        '(Intercept)'        'std'        0.12346     0.053269    0.28615

Group: Error
    Name             Estimate    Lower      Upper
    'Res Std'        0.21502     0.16209    0.28522


 Contrast: fb success vs fb neutral      ROI: amygdala 5D-ASC subscore: asc_aud

lme =


Linear mixed-effects model fit by REML

Model information:
    Number of observations              54
    Fixed effects coefficients           8
    Random effects coefficients         27
    Covariance parameters                2

Formula:
    ROI ~ 1 + measurement + drug + ketLvl + sex + age + drugOrder + fiveDASC + (1 | subject)

Model fit statistics:
    AIC       BIC       LogLikelihood    Deviance
    59.681    77.967    -19.84           39.681

Fixed effects coefficients (95% CIs):
    Name                 Estimate      SE             tStat       DF    pValue      Lower
    '(Intercept)'        -0.027217        0.1648    -0.16515    46     0.86955       -0.35895
    'measurement'        -0.068742      0.057813      -1.189    46     0.24053       -0.18511
    'drug'                -0.27177       0.29389    -0.92473    46     0.35993       -0.86334
    'ketLvl'             1.0727e-05    1.4086e-05      0.7615    46     0.45025    -1.7627e-05
    'sex'                -0.073291      0.083077    -0.88221    46     0.38225       -0.24051
    'age'                 0.025403      0.054927     0.46249    46     0.64591      -0.085158
    'drugOrder'            0.15728      0.077995      2.0165    46    0.049605     0.00028356
    'fiveDASC'           -0.045421      0.033558     -1.3535    46     0.18251       -0.11297


    Upper
       0.30452
       0.04763
        0.3198
    3.9081e-05
      0.093934
       0.13596
       0.31427
      0.022128

Random effects covariance parameters (95% CIs):
Group: subject (27 Levels)
    Name1                Name2                Type         Estimate    Lower       Upper
    '(Intercept)'        '(Intercept)'        'std'        0.1339      0.065115    0.27537

Group: Error
    Name             Estimate    Lower      Upper
    'Res Std'        0.20586     0.15494    0.27351


 Contrast: fb success vs fb neutral      ROI: amygdala 5D-ASC subscore: asc_vig
```

```
lme = 


Linear mixed-effects model fit by REML

Model information:
    Number of observations              54
    Fixed effects coefficients           8
    Random effects coefficients         27
    Covariance parameters                2

Formula:
    ROI ~ 1 + measurement + drug + ketLvl + sex + age + drugOrder + fiveDASC + (1 | subject)

Model fit statistics:
    AIC       BIC      LogLikelihood    Deviance
    61.244    79.53    -20.622          41.244  

Fixed effects coefficients (95% CIs):
    Name                   Estimate      SE             tStat       DF    pValue      Lower     
    '(Intercept)'           -0.056657       0.16443    -0.34456    46       0.732       -0.38764
    'measurement'           -0.045052      0.062059    -0.72596    46     0.47154       -0.16997
    'drug'                   -0.21981       0.30028    -0.73204    46     0.46786       -0.82424
    'ketLvl'               8.2339e-06    1.4381e-05     0.57257    46     0.56972    -2.0713e-05
    'sex'                   -0.072223      0.082001    -0.88076    46     0.38303       -0.23728
    'age'                    0.030807      0.053957     0.57095    46     0.57081      -0.077803
    'drugOrder'               0.15325       0.07804      1.9638    46    0.055618     -0.0038341
    'fiveDASC'               0.011967      0.035326     0.33877    46     0.73633      -0.059141


    Upper     
       0.27433
      0.079867
       0.38461
    3.7181e-05
      0.092836
       0.13942
       0.31034
      0.083076

Random effects covariance parameters (95% CIs):
Group: subject (27 Levels)
    Name1                  Name2                   Type         Estimate    Lower      Upper  
    '(Intercept)'          '(Intercept)'           'std'        0.12033     0.04897    0.29569

Group: Error
    Name               Estimate    Lower      Upper  
    'Res Std'          0.21628     0.16269    0.28754


 Contrast: fb success vs fb neutral      ROI: amygdala 5D-ASC subscore: sum

lme = 


Linear mixed-effects model fit by REML

Model information:
    Number of observations              54
    Fixed effects coefficients           8
    Random effects coefficients         27
    Covariance parameters                2

Formula:
    ROI ~ 1 + measurement + drug + ketLvl + sex + age + drugOrder + fiveDASC + (1 | subject)

Model fit statistics:
    AIC       BIC       LogLikelihood    Deviance
    61.261    79.547    -20.63           41.261  

Fixed effects coefficients (95% CIs):
    Name                   Estimate      SE             tStat       DF    pValue      Lower     
    '(Intercept)'            -0.05838       0.16511    -0.35358    46     0.72527       -0.39073
    'measurement'           -0.045581      0.062276    -0.73192    46     0.46793       -0.17094
    'drug'                   -0.21023       0.30115    -0.69809    46     0.48864        -0.8164
    'ketLvl'               7.7651e-06    1.4422e-05     0.53843    46     0.59287    -2.1264e-05
    'sex'                   -0.072731      0.082301    -0.88371    46     0.38145       -0.23839
```

```
    'age'                  0.028994      0.054314     0.53381    46     0.59604      -0.080335
    'drugOrder'             0.15488      0.077787       1.991    46    0.052435        -0.0017
    'fiveDASC'             0.010338      0.035491     0.29129    46     0.77214      -0.061101


    Upper
       0.27397
      0.079774
       0.39595
    3.6794e-05
      0.092933
       0.13832
       0.31145
      0.081777

Random effects covariance parameters (95% CIs):
Group: subject (27 Levels)
    Name1                Name2                Type         Estimate    Lower       Upper
    '(Intercept)'        '(Intercept)'        'std'        0.122       0.051399    0.2896

Group: Error
    Name             Estimate    Lower      Upper
    'Res Std'        0.21568     0.16255    0.28616


 Contrast: fb failure vs fb neutral      ROI: striatum 5D-ASC subscore: asc_obn

lme =


Linear mixed-effects model fit by REML

Model information:
    Number of observations              54
    Fixed effects coefficients           8
    Random effects coefficients         27
    Covariance parameters                2

Formula:
    ROI ~ 1 + measurement + drug + ketLvl + sex + age + drugOrder + fiveDASC + (1 | subject)

Model fit statistics:
    AIC      BIC       LogLikelihood    Deviance
    16.11    34.396    1.9451           -3.8902

Fixed effects coefficients (95% CIs):
    Name                 Estimate       SE            tStat       DF    pValue       Lower
    '(Intercept)'           -0.29872       0.10519     -2.8397    46    0.0067022       -0.51046
    'measurement'           0.076745      0.034115      2.2496    46     0.029305      0.0080742
    'drug'                   0.15011       0.17691     0.84851    46      0.40055       -0.20599
    'ketLvl'             -8.4028e-06    8.4882e-06    -0.98994    46      0.32738    -2.5489e-05
    'sex'                   0.015795      0.054882     0.28779    46       0.7748      -0.094678
    'age'                   0.050359      0.036524      1.3788    46      0.17462       -0.02316
    'drugOrder'             0.051317      0.051902     0.98873    46      0.32797      -0.053156
    'fiveDASC'              0.032383      0.020232      1.6006    46      0.11632     -0.0083421


    Upper
     -0.086978
       0.14542
        0.5062
    8.6831e-06
       0.12627
       0.12388
       0.15579
      0.073109

Random effects covariance parameters (95% CIs):
Group: subject (27 Levels)
    Name1                Name2                Type         Estimate    Lower       Upper
    '(Intercept)'        '(Intercept)'        'std'        0.099893    0.055552    0.17963

Group: Error
    Name             Estimate    Lower       Upper
    'Res Std'        0.12057     0.089504    0.16241


 Contrast: fb failure vs fb neutral      ROI: striatum 5D-ASC subscore: asc_aed
```

```
lme =


Linear mixed-effects model fit by REML

Model information:
    Number of observations              54
    Fixed effects coefficients           8
    Random effects coefficients         27
    Covariance parameters                2

Formula:
    ROI ~ 1 + measurement + drug + ketLvl + sex + age + drugOrder + fiveDASC + (1 | subject)

Model fit statistics:
    AIC       BIC       LogLikelihood    Deviance
    18.157    36.444    0.92129          -1.8426

Fixed effects coefficients (95% CIs):
    Name                 Estimate       SE            tStat       DF    pValue       Lower
    '(Intercept)'           -0.28674       0.10489     -2.7337    46    0.0088599       -0.49787
    'measurement'           0.064694      0.036103      1.7919    46     0.079727     -0.0079783
    'drug'                    0.1569       0.18591     0.84396    46      0.40306       -0.21732
    'ketLvl'             -8.7562e-06    8.9136e-06    -0.98234    46      0.33107    -2.6698e-05
    'sex'                   0.021236      0.053682      0.3956    46      0.69423      -0.086819
    'age'                   0.054625      0.035697      1.5302    46      0.13281       -0.01723
    'drugOrder'             0.054389      0.051094      1.0645    46      0.29265      -0.048456
    'fiveDASC'             0.0063849      0.021484      0.2972    46      0.76765      -0.036859


    Upper
    -0.075606
      0.13737
      0.53112
    9.186e-06
      0.12929
      0.12648
      0.15724
     0.049629

Random effects covariance parameters (95% CIs):
Group: subject (27 Levels)
    Name1                Name2                Type         Estimate    Lower       Upper
    '(Intercept)'        '(Intercept)'        'std'        0.089652    0.041712    0.19269

Group: Error
    Name             Estimate    Lower       Upper
    'Res Std'        0.12916     0.094864    0.17586


 Contrast: fb failure vs fb neutral      ROI: striatum 5D-ASC subscore: asc_vis

lme =


Linear mixed-effects model fit by REML

Model information:
    Number of observations              54
    Fixed effects coefficients           8
    Random effects coefficients         27
    Covariance parameters                2

Formula:
    ROI ~ 1 + measurement + drug + ketLvl + sex + age + drugOrder + fiveDASC + (1 | subject)

Model fit statistics:
    AIC       BIC       LogLikelihood    Deviance
    17.995    36.282    1.0023           -2.0045

Fixed effects coefficients (95% CIs):
    Name                 Estimate       SE            tStat       DF    pValue       Lower
    '(Intercept)'           -0.29152       0.10486     -2.7799    46    0.0078507        -0.5026
    'measurement'            0.06907      0.038082      1.8137    46     0.076244     -0.0075841
    'drug'                   0.15537       0.18462     0.84153    46       0.4044       -0.21626
    'ketLvl'             -8.6734e-06    8.8493e-06    -0.98013    46      0.33215    -2.6486e-05
    'sex'                   0.020028      0.053283     0.37588    46      0.70874      -0.087225
```

```
    'age'                  0.05441      0.035272      1.5426    46      0.12978      -0.016588
    'drugOrder'           0.053492      0.050451      1.0603    46      0.29456      -0.048062
    'fiveDASC'            0.010424      0.021511     0.48458    46      0.63027      -0.032876


    Upper
     -0.080434
       0.14572
         0.527
    9.1393e-06
       0.12728
       0.12541
       0.15505
      0.053723

Random effects covariance parameters (95% CIs):
Group: subject (27 Levels)
    Name1                Name2                Type         Estimate    Lower       Upper
    '(Intercept)'        '(Intercept)'        'std'        0.087471    0.042577    0.1797

Group: Error
    Name             Estimate    Lower       Upper
    'Res Std'        0.12985     0.096981    0.17385


 Contrast: fb failure vs fb neutral      ROI: striatum 5D-ASC subscore: asc_aud

lme =


Linear mixed-effects model fit by REML

Model information:
    Number of observations              54
    Fixed effects coefficients           8
    Random effects coefficients         27
    Covariance parameters                2

Formula:
    ROI ~ 1 + measurement + drug + ketLvl + sex + age + drugOrder + fiveDASC + (1 | subject)

Model fit statistics:
    AIC       BIC       LogLikelihood    Deviance
    17.499    35.786    1.2503           -2.5006

Fixed effects coefficients (95% CIs):
    Name                 Estimate       SE            tStat       DF    pValue      Lower
    '(Intercept)'           -0.27556       0.10441     -2.6393    46    0.011303       -0.48572
    'measurement'           0.055369      0.036391      1.5215    46     0.13497      -0.017882
    'drug'                   0.13424       0.18546     0.72383    46     0.47283       -0.23906
    'ketLvl'             -7.6671e-06    8.8898e-06    -0.86246    46     0.39291    -2.5561e-05
    'sex'                   0.020947      0.052759     0.39703    46     0.69318       -0.08525
    'age'                   0.053936      0.034896      1.5456    46     0.12904      -0.016305
    'drugOrder'             0.056403      0.049554      1.1382    46     0.26092      -0.043344
    'fiveDASC'             -0.018392      0.021202     -0.8675    46     0.39017      -0.061069


    Upper
     -0.065402
       0.12862
       0.50754
    1.0227e-05
       0.12714
       0.12418
       0.15615
      0.024284

Random effects covariance parameters (95% CIs):
Group: subject (27 Levels)
    Name1                Name2                Type         Estimate    Lower       Upper
    '(Intercept)'        '(Intercept)'        'std'        0.086021    0.041569    0.17801

Group: Error
    Name             Estimate    Lower       Upper
    'Res Std'        0.12955     0.096978    0.17306


 Contrast: fb failure vs fb neutral      ROI: striatum 5D-ASC subscore: asc_vig
```

```
lme = 


Linear mixed-effects model fit by REML

Model information:
    Number of observations              54
    Fixed effects coefficients           8
    Random effects coefficients         27
    Covariance parameters                2

Formula:
    ROI ~ 1 + measurement + drug + ketLvl + sex + age + drugOrder + fiveDASC + (1 | subject)

Model fit statistics:
    AIC       BIC       LogLikelihood    Deviance
    18.071    36.357    0.96454          -1.9291 

Fixed effects coefficients (95% CIs):
    Name                   Estimate       SE            tStat       DF    pValue       Lower      
    '(Intercept)'             -0.28496       0.10361     -2.7504    46    0.0084813       -0.49351
    'measurement'             0.057933      0.038185      1.5171    46      0.13607       -0.01893
    'drug'                     0.16775       0.18654     0.89929    46      0.37318       -0.20773
    'ketLvl'               -9.2981e-06    8.9378e-06     -1.0403    46      0.30364    -2.7289e-05
    'sex'                     0.022359      0.052198     0.42835    46       0.6704       -0.08271
    'age'                     0.055699      0.034405      1.6189    46      0.11231      -0.013556
    'drugOrder'               0.059902      0.049733      1.2045    46      0.23457      -0.040206
    'fiveDASC'              -0.0081053      0.022068    -0.36728    46      0.71509      -0.052527


    Upper     
     -0.076415
        0.1348
       0.54324
    8.6929e-06
       0.12743
       0.12495
       0.16001
      0.036316

Random effects covariance parameters (95% CIs):
Group: subject (27 Levels)
    Name1                Name2                Type         Estimate    Lower       Upper  
    '(Intercept)'        '(Intercept)'        'std'        0.081108    0.033493    0.19642

Group: Error
    Name             Estimate    Lower       Upper  
    'Res Std'        0.13284     0.098086    0.17991


 Contrast: fb failure vs fb neutral      ROI: striatum 5D-ASC subscore: sum

lme = 


Linear mixed-effects model fit by REML

Model information:
    Number of observations              54
    Fixed effects coefficients           8
    Random effects coefficients         27
    Covariance parameters                2

Formula:
    ROI ~ 1 + measurement + drug + ketLvl + sex + age + drugOrder + fiveDASC + (1 | subject)

Model fit statistics:
    AIC       BIC       LogLikelihood    Deviance
    17.669    35.955    1.1655           -2.331  

Fixed effects coefficients (95% CIs):
    Name                   Estimate       SE            tStat       DF    pValue       Lower      
    '(Intercept)'             -0.29331       0.10577      -2.773    46    0.0079937       -0.50623
    'measurement'              0.07238      0.036591      1.9781    46     0.053929     -0.0012744
    'drug'                      0.1499       0.18231     0.82223    46      0.41519       -0.21707
    'ketLvl'               -8.4006e-06    8.7428e-06    -0.96086    46      0.34165    -2.5999e-05
    'sex'                     0.018883      0.054512      0.3464    46      0.73062      -0.090845
```

```
    'age'                  0.053525      0.036173      1.4797    46      0.14577      -0.019288
    'drugOrder'            0.051566      0.051768      0.9961    46      0.32441      -0.052638
    'fiveDASC'              0.01717      0.021841     0.78614    46      0.43582      -0.026794


    Upper
     -0.080404
       0.14603
       0.51687
    9.1978e-06
       0.12861
       0.12634
       0.15577
      0.061135

Random effects covariance parameters (95% CIs):
Group: subject (27 Levels)
    Name1               Name2               Type        Estimate    Lower      Upper
    '(Intercept)'       '(Intercept)'       'std'       0.094958    0.04842    0.18622

Group: Error
    Name            Estimate    Lower       Upper
    'Res Std'       0.12593     0.092991    0.17054


 Contrast: fb failure vs fb neutral      ROI: nucleus accumbens 5D-ASC subscore: asc_obn

lme =


Linear mixed-effects model fit by REML

Model information:
    Number of observations             54
    Fixed effects coefficients          8
    Random effects coefficients        27
    Covariance parameters               2

Formula:
    ROI ~ 1 + measurement + drug + ketLvl + sex + age + drugOrder + fiveDASC + (1 | subject)

Model fit statistics:
    AIC       BIC       LogLikelihood    Deviance
    57.391    75.678    -18.696          37.391

Fixed effects coefficients (95% CIs):
    Name                Estimate      SE            tStat        DF    pValue      Lower
    '(Intercept)'         -0.27625       0.15296       -1.806    46    0.077464       -0.58415
    'measurement'         0.086381      0.065195        1.325    46     0.19172      -0.044848
    'drug'                0.038451       0.29781      0.12911    46     0.89783       -0.56101
    'ketLvl'            6.1637e-07    1.4208e-05     0.043381    46     0.96559    -2.7983e-05
    'sex'                 0.030951      0.070334      0.44005    46     0.66196       -0.11063
    'age'                 0.087352      0.045967       1.9003    46    0.063667     -0.0051742
    'drugOrder'         -0.0059613      0.065185    -0.091452    46     0.92753       -0.13717
    'fiveDASC'           -0.027451      0.033186     -0.82721    46     0.41239      -0.094251


    Upper
      0.031648
       0.21761
       0.63791
    2.9216e-05
       0.17253
       0.17988
       0.12525
      0.039348

Random effects covariance parameters (95% CIs):
Group: subject (27 Levels)
    Name1               Name2               Type        Estimate      Lower
    '(Intercept)'       '(Intercept)'       'std'       0.00036497    5.7712e-287


    Upper
    2.308e+279

Group: Error
    Name            Estimate    Lower      Upper
```

```
    'Res Std'        0.23282     0.18979    0.2856


 Contrast: fb failure vs fb neutral      ROI: nucleus accumbens 5D-ASC subscore: asc_aed

lme =


Linear mixed-effects model fit by REML

Model information:
    Number of observations             54
    Fixed effects coefficients          8
    Random effects coefficients        27
    Covariance parameters               2

Formula:
    ROI ~ 1 + measurement + drug + ketLvl + sex + age + drugOrder + fiveDASC + (1 | subject)

Model fit statistics:
    AIC       BIC       LogLikelihood    Deviance
    56.501    74.788    -18.251          36.501

Fixed effects coefficients (95% CIs):
    Name                 Estimate      SE            tStat       DF    pValue      Lower
    '(Intercept)'           -0.2856       0.15105     -1.8907    46    0.064972       -0.58965
    'measurement'          0.084087      0.064048      1.3129    46     0.19574      -0.044835
    'drug'                0.0042332       0.29729    0.014239    46      0.9887       -0.59417
    'ketLvl'             2.2821e-06    1.4188e-05     0.16085    46     0.87291    -2.6276e-05
    'sex'                  0.025768       0.06964     0.37002    46     0.71307       -0.11441
    'age'                  0.090812      0.045666      1.9886    46    0.052714     -0.0011095
    'drugOrder'           0.0048523      0.065517    0.074061    46     0.94128       -0.12703
    'fiveDASC'            -0.041739      0.033334     -1.2521    46     0.21686       -0.10884


    Upper
     0.018457
      0.21301
      0.60264
    3.084e-05
      0.16595
      0.18273
      0.13673
      0.02536

Random effects covariance parameters (95% CIs):
Group: subject (27 Levels)
    Name1                Name2                Type         Estimate      Lower
    '(Intercept)'        '(Intercept)'        'std'        0.00020558    3.3113e-301


    Upper
    1.2763e+293

Group: Error
    Name             Estimate    Lower      Upper
    'Res Std'        0.23065     0.18802    0.28294


 Contrast: fb failure vs fb neutral      ROI: nucleus accumbens 5D-ASC subscore: asc_vis

lme =


Linear mixed-effects model fit by REML

Model information:
    Number of observations             54
    Fixed effects coefficients          8
    Random effects coefficients        27
    Covariance parameters               2

Formula:
    ROI ~ 1 + measurement + drug + ketLvl + sex + age + drugOrder + fiveDASC + (1 | subject)

Model fit statistics:
    AIC       BIC       LogLikelihood    Deviance
    56.824    75.111    -18.412          36.824
```

Fixed effects coefficients (95% CIs):

Name	Estimate	SE	tStat	DF	pValue	Lower	Upper
'(Intercept)'	-0.26872	0.15249	-1.7623	46	0.084666	-0.57566	0.038215
'measurement'	0.07435	0.066887	1.1116	46	0.2721	-0.060287	0.20899
'drug'	0.053679	0.29486	0.18205	46	0.85635	-0.53985	0.6472
'ketLvl'	-1.4848e-07	1.4066e-05	-0.010556	46	0.99162	-2.8462e-05	2.8165e-05
'sex'	0.032595	0.069985	0.46574	46	0.6436	-0.10828	0.17347
'age'	0.088248	0.045665	1.9325	46	0.059468	-0.003671	0.18017
'drugOrder'	0.00087832	0.065436	0.013423	46	0.98935	-0.13084	0.13259
'fiveDASC'	-0.037287	0.034017	-1.0961	46	0.27873	-0.10576	0.031186

Random effects covariance parameters (95% CIs):
Group: subject (27 Levels)

Name1	Name2	Type	Estimate	Lower	Upper
'(Intercept)'	'(Intercept)'	'std'	0.00032842	2.3289e-283	4.6314e+275

Group: Error

Name	Estimate	Lower	Upper
'Res Std'	0.23154	0.18875	0.28404

Contrast: fb failure vs fb neutral   ROI: nucleus accumbens 5D-ASC subscore: asc_aud

lme =

Linear mixed-effects model fit by REML

Model information:

Number of observations	54
Fixed effects coefficients	8
Random effects coefficients	27
Covariance parameters	2

Formula:
ROI ~ 1 + measurement + drug + ketLvl + sex + age + drugOrder + fiveDASC + (1 | subject)

Model fit statistics:

AIC	BIC	LogLikelihood	Deviance
51.332	69.619	-15.666	31.332

Fixed effects coefficients (95% CIs):

Name	Estimate	SE	tStat	DF	pValue	Lower	Upper
'(Intercept)'	-0.23686	0.14388	-1.6463	46	0.10652	-0.52648	0.052749
'measurement'	0.066133	0.060752	1.0886	46	0.28202	-0.056155	0.18842
'drug'	-0.025611	0.27931	-0.091695	46	0.92734	-0.58783	0.5366
'ketLvl'	3.7061e-06	1.3327e-05	0.2781	46	0.78218	-2.3119e-05	3.0531e-05
'sex'	0.026841	0.065766	0.40812	46	0.68508	-0.10554	0.15922
'age'	0.074208	0.042862	1.7313	46	0.090093	-0.012068	0.16048
'drugOrder'	-0.012233	0.060771	-0.20129	46	0.84136	-0.13456	0.11009
'fiveDASC'	-0.082574	0.030576	-2.7006	46	0.0096539	-0.14412	-0.021029

Random effects covariance parameters (95% CIs):
Group: subject (27 Levels)

Name1	Name2	Type	Estimate	Lower	Upper
'(Intercept)'	'(Intercept)'	'std'	0.0003164	1.2369e-271	8.0939e+263

Group: Error

Name	Estimate	Lower	Upper
'Res Std'	0.21791	0.17763	0.26731

Contrast: fb failure vs fb neutral   ROI: nucleus accumbens 5D-ASC subscore: asc_vig

lme =

Linear mixed-effects model fit by REML

Model information:

Number of observations	54
Fixed effects coefficients	8
Random effects coefficients	27
Covariance parameters	2

Formula:
ROI ~ 1 + measurement + drug + ketLvl + sex + age + drugOrder + fiveDASC + (1 | subject)

Model fit statistics:

AIC	BIC	LogLikelihood	Deviance
57.41	75.696	-18.705	37.41

Fixed effects coefficients (95% CIs):

Name	Estimate	SE	tStat	DF	pValue	Lower	Upper
'(Intercept)'	-0.28132	0.15267	-1.8427	46	0.071828	-0.58864	0.025989
'measurement'	0.083936	0.066061	1.2706	46	0.21026	-0.049037	0.21691
'drug'	0.058709	0.29655	0.19797	46	0.84394	-0.53821	0.65563
'ketLvl'	-3.7667e-07	1.4147e-05	-0.026625	46	0.97887	-2.8853e-05	2.81e-05
'sex'	0.028144	0.070297	0.40036	46	0.69075	-0.11336	0.16964
'age'	0.081747	0.04571	1.7884	46	0.0803	-0.010262	0.17376
'drugOrder'	0.00012412	0.066358	0.0018704	46	0.99852	-0.13345	0.1337
'fiveDASC'	-0.026917	0.033585	-0.80148	46	0.42698	-0.09452	0.040685

Random effects covariance parameters (95% CIs):
Group: subject (27 Levels)

Name1	Name2	Type	Estimate	Lower	Upper
'(Intercept)'	'(Intercept)'	'std'	0.000419	4.3756e-303	4.0123e+295

Group: Error

Name	Estimate	Lower	Upper
'Res Std'	0.23292	0.18987	0.28573

Contrast: fb failure vs fb neutral   ROI: nucleus accumbens 5D-ASC subscore: sum

lme =

Linear mixed-effects model fit by REML

```
Model information:
    Number of observations             54
    Fixed effects coefficients          8
    Random effects coefficients        27
    Covariance parameters               2

Formula:
    ROI ~ 1 + measurement + drug + ketLvl + sex + age + drugOrder + fiveDASC + (1 | subject)

Model fit statistics:
    AIC       BIC       LogLikelihood    Deviance
    56.144    74.431    -18.072          36.144

Fixed effects coefficients (95% CIs):
    Name                    Estimate      SE            tStat       DF    pValue      Lower
    '(Intercept)'             -0.26852       0.15104     -1.7778    46    0.082052       -0.57256
    'measurement'               0.0722      0.065541      1.1016    46     0.27636      -0.059727
    'drug'                    0.019235        0.2942    0.065382    46     0.94815       -0.57296
    'ketLvl'                1.5285e-06    1.4035e-05      0.1089    46     0.91375    -2.6723e-05
    'sex'                     0.030765      0.069375     0.44346    46     0.65951       -0.10888
    'age'                     0.088741      0.045261      1.9606    46    0.055993     -0.0023653
    'drugOrder'              0.0036074      0.064917    0.055569    46     0.95593       -0.12706
    'fiveDASC'               -0.046426      0.033505     -1.3856    46     0.17255       -0.11387


    Upper
     0.035514
      0.20413
      0.61143
    2.978e-05
      0.17041
      0.17985
      0.13428
     0.021017

Random effects covariance parameters (95% CIs):
Group: subject (27 Levels)
    Name1                   Name2                   Type         Estimate      Lower
    '(Intercept)'           '(Intercept)'           'std'        0.00020536    4.3526e-289


    Upper
    9.6894e+280

Group: Error
    Name               Estimate    Lower      Upper
    'Res Std'          0.2298      0.18733    0.2819


 Contrast: fb failure vs fb neutral      ROI: insula 5D-ASC subscore: asc_obn

lme =


Linear mixed-effects model fit by REML

Model information:
    Number of observations             54
    Fixed effects coefficients          8
    Random effects coefficients        27
    Covariance parameters               2

Formula:
    ROI ~ 1 + measurement + drug + ketLvl + sex + age + drugOrder + fiveDASC + (1 | subject)

Model fit statistics:
    AIC       BIC       LogLikelihood    Deviance
    81.414    99.701    -30.707          61.414

Fixed effects coefficients (95% CIs):
    Name                    Estimate       SE            tStat       DF    pValue      Lower
    '(Intercept)'              -0.35445       0.22334     -1.5871    46     0.11935       -0.80401
    'measurement'               0.15545       0.06461       2.406    46    0.020202       0.025401
    'drug'                     0.009992       0.34472    0.028986    46       0.977        -0.6839
    'ketLvl'                -2.6963e-06    1.6558e-05    -0.16283    46     0.87136    -3.6026e-05
    'sex'                      0.014836       0.11983     0.12381    46     0.90201       -0.22637
    'age'                       0.11325      0.080152       1.413    46     0.16439      -0.048084
    'drugOrder'                 0.15585       0.11397      1.3675    46     0.17812      -0.073555
```

'fiveDASC'	0.06134	0.039691	1.5454	46	0.12909	-0.018554

Upper
0.095103
0.28551
0.70388
3.0634e-05
0.25605
0.27459
0.38525
0.14123

Random effects covariance parameters (95% CIs):
Group: subject (27 Levels)

Name1	Name2	Type	Estimate	Lower	Upper
'(Intercept)'	'(Intercept)'	'std'	0.23944	0.15365	0.37313

Group: Error

Name	Estimate	Lower	Upper
'Res Std'	0.22766	0.17091	0.30326

Contrast: fb failure vs fb neutral   ROI: insula 5D-ASC subscore: asc_aed

lme =

Linear mixed-effects model fit by REML

Model information:

Number of observations	54
Fixed effects coefficients	8
Random effects coefficients	27
Covariance parameters	2

Formula:
ROI ~ 1 + measurement + drug + ketLvl + sex + age + drugOrder + fiveDASC + (1 | subject)

Model fit statistics:

AIC	BIC	LogLikelihood	Deviance
83.348	101.63	-31.674	63.348

Fixed effects coefficients (95% CIs):

Name	Estimate	SE	tStat	DF	pValue	Lower
'(Intercept)'	-0.33228	0.22612	-1.4695	46	0.14851	-0.78745
'measurement'	0.13746	0.065997	2.0828	46	0.042858	0.0046125
'drug'	-0.0054702	0.3557	-0.015379	46	0.9878	-0.72147
'ketLvl'	-1.9742e-06	1.7085e-05	-0.11555	46	0.90851	-3.6364e-05
'sex'	0.022482	0.12107	0.1857	46	0.8535	-0.22122
'age'	0.11858	0.081102	1.4621	46	0.15051	-0.044671
'drugOrder'	0.15757	0.11594	1.359	46	0.18076	-0.075807
'fiveDASC'	0.023915	0.041757	0.5727	46	0.56964	-0.060139

Upper
0.12288
0.2703
0.71052
3.2415e-05
0.26618
0.28183
0.39094
0.10797

Random effects covariance parameters (95% CIs):
Group: subject (27 Levels)

Name1	Name2	Type	Estimate	Lower	Upper
'(Intercept)'	'(Intercept)'	'std'	0.23929	0.15176	0.37728

Group: Error

Name	Estimate	Lower	Upper
'Res Std'	0.23522	0.17661	0.31328

Contrast: fb failure vs fb neutral   ROI: insula 5D-ASC subscore: asc_vis

lme =

```
Linear mixed-effects model fit by REML

Model information:
    Number of observations              54
    Fixed effects coefficients           8
    Random effects coefficients         27
    Covariance parameters                2

Formula:
    ROI ~ 1 + measurement + drug + ketLvl + sex + age + drugOrder + fiveDASC + (1 | subject)

Model fit statistics:
    AIC       BIC       LogLikelihood    Deviance
    81.451    99.737    -30.725          61.451

Fixed effects coefficients (95% CIs):
    Name                 Estimate      SE            tStat        DF    pValue      Lower
    '(Intercept)'          -0.36094       0.22139      -1.6303    46     0.10986       -0.80659
    'measurement'           0.16839      0.068594       2.4549    46    0.017935       0.030318
    'drug'                -0.017444       0.34751    -0.050196    46     0.96018       -0.71695
    'ketLvl'             -1.334e-06    1.6688e-05    -0.079933    46     0.93664    -3.4926e-05
    'sex'                   0.01451       0.11792      0.12306    46      0.9026       -0.22284
    'age'                   0.11445       0.07871       1.4541    46     0.15272      -0.043985
    'drugOrder'             0.14724       0.11247       1.3091    46     0.19699      -0.079151
    'fiveDASC'              0.06165      0.040684       1.5153    46     0.13654      -0.020244


    Upper
      0.084701
       0.30646
       0.68206
    3.2258e-05
       0.25186
       0.27288
       0.37363
       0.14354

Random effects covariance parameters (95% CIs):
Group: subject (27 Levels)
    Name1                Name2                Type         Estimate    Lower      Upper
    '(Intercept)'        '(Intercept)'        'std'        0.23118     0.14528    0.36786

Group: Error
    Name             Estimate    Lower      Upper
    'Res Std'        0.23192     0.17396    0.30918


 Contrast: fb failure vs fb neutral      ROI: insula 5D-ASC subscore: asc_aud

lme =


Linear mixed-effects model fit by REML

Model information:
    Number of observations              54
    Fixed effects coefficients           8
    Random effects coefficients         27
    Covariance parameters                2

Formula:
    ROI ~ 1 + measurement + drug + ketLvl + sex + age + drugOrder + fiveDASC + (1 | subject)

Model fit statistics:
    AIC       BIC       LogLikelihood    Deviance
    82.475    100.76    -31.237          62.475

Fixed effects coefficients (95% CIs):
    Name                 Estimate      SE            tStat        DF    pValue      Lower
    '(Intercept)'           -0.35927       0.22366      -1.6063    46     0.11505       -0.80948
    'measurement'            0.14676      0.066599       2.2037    46    0.032592       0.012706
    'drug'                  0.034511       0.35632     0.096855    46     0.92326       -0.68272
    'ketLvl'             -3.9085e-06    1.7112e-05     -0.22841    46     0.82034    -3.8353e-05
    'sex'                    0.02368       0.11848      0.19987    46     0.84246        -0.2148
    'age'                    0.12825      0.079029       1.6228    46     0.11147      -0.030829
    'drugOrder'              0.16716       0.11233       1.4881    46     0.14354      -0.058948
```

```
    'fiveDASC'              0.046037      0.041767      1.1022    46     0.27609      -0.038035


    Upper
      0.090941
       0.28082
       0.75175
    3.0536e-05
       0.26216
       0.28733
       0.39327
       0.13011

Random effects covariance parameters (95% CIs):
Group: subject (27 Levels)
    Name1                Name2                Type         Estimate    Lower    Upper
    '(Intercept)'        '(Intercept)'        'std'        0.2309      0.144    0.37024

Group: Error
    Name             Estimate    Lower      Upper
    'Res Std'        0.236       0.17715    0.3144


 Contrast: fb failure vs fb neutral      ROI: insula 5D-ASC subscore: asc_vig

lme =


Linear mixed-effects model fit by REML

Model information:
    Number of observations             54
    Fixed effects coefficients          8
    Random effects coefficients        27
    Covariance parameters               2

Formula:
    ROI ~ 1 + measurement + drug + ketLvl + sex + age + drugOrder + fiveDASC + (1 | subject)

Model fit statistics:
    AIC       BIC       LogLikelihood    Deviance
    83.394    101.68    -31.697          63.394

Fixed effects coefficients (95% CIs):
    Name                 Estimate       SE            tStat        DF    pValue      Lower
    '(Intercept)'          -0.32803       0.22601      -1.4514    46     0.15347       -0.78297
    'measurement'           0.11884      0.068837       1.7264    46    0.090995      -0.019725
    'drug'                -0.016847       0.35534    -0.047411    46     0.96239       -0.73212
    'ketLvl'            -1.4526e-06    1.7069e-05    -0.085102    46     0.93255    -3.5812e-05
    'sex'                  0.022717       0.12077       0.1881    46     0.85162       -0.22037
    'age'                   0.12246      0.080491       1.5214    46     0.13501      -0.039563
    'drugOrder'             0.17423       0.11589       1.5034    46     0.13956      -0.059041
    'fiveDASC'            -0.019562      0.043588     -0.44879    46     0.65569        -0.1073


    Upper
       0.12692
        0.2574
       0.69842
    3.2906e-05
       0.26581
       0.28448
        0.4075
      0.068177

Random effects covariance parameters (95% CIs):
Group: subject (27 Levels)
    Name1                Name2                Type         Estimate    Lower      Upper
    '(Intercept)'        '(Intercept)'        'std'        0.23764     0.15018    0.37603

Group: Error
    Name             Estimate    Lower      Upper
    'Res Std'        0.2365      0.17774    0.31467


 Contrast: fb failure vs fb neutral      ROI: insula 5D-ASC subscore: sum

lme =
```

```
Linear mixed-effects model fit by REML

Model information:
    Number of observations             54
    Fixed effects coefficients          8
    Random effects coefficients        27
    Covariance parameters               2

Formula:
    ROI ~ 1 + measurement + drug + ketLvl + sex + age + drugOrder + fiveDASC + (1 | subject)

Model fit statistics:
    AIC       BIC       LogLikelihood    Deviance
    82.004    100.29    -31.002          62.004

Fixed effects coefficients (95% CIs):
    Name                 Estimate       SE            tStat         DF    pValue
    '(Intercept)'          -0.35287       0.22473       -1.5702    46     0.12322
    'measurement'           0.16003      0.067445        2.3727    46    0.021895
    'drug'               -0.0032591       0.34762    -0.0093756    46     0.99256
    'ketLvl'            -2.0413e-06    1.6696e-05      -0.12226    46     0.90322
    'sex'                  0.017027       0.12036       0.14146    46     0.88812
    'age'                   0.11613      0.080436        1.4438    46     0.15558
    'drugOrder'             0.14977         0.115        1.3023    46     0.19928
    'fiveDASC'             0.054467      0.042542        1.2803    46     0.20685


    Lower          Upper
       -0.80523      0.099487
       0.024267       0.29579
       -0.70298       0.69646
    -3.5648e-05    3.1566e-05
       -0.22525       0.25931
      -0.045779       0.27804
      -0.081715       0.38126
      -0.031165        0.1401

Random effects covariance parameters (95% CIs):
Group: subject (27 Levels)
    Name1                Name2                Type         Estimate    Lower      Upper
    '(Intercept)'        '(Intercept)'        'std'        0.23977     0.15325    0.37514

Group: Error
    Name             Estimate    Lower      Upper
    'Res Std'        0.23016     0.17282    0.30652


 Contrast: fb failure vs fb neutral      ROI: thalamus 5D-ASC subscore: asc_obn

lme =


Linear mixed-effects model fit by REML

Model information:
    Number of observations             54
    Fixed effects coefficients          8
    Random effects coefficients        27
    Covariance parameters               2

Formula:
    ROI ~ 1 + measurement + drug + ketLvl + sex + age + drugOrder + fiveDASC + (1 | subject)

Model fit statistics:
    AIC        BIC      LogLikelihood    Deviance
    -2.2565    16.03    11.128           -22.256

Fixed effects coefficients (95% CIs):
    Name                 Estimate      SE            tStat       DF    pValue      Lower
    '(Intercept)'         -0.072622      0.079986    -0.90794    46     0.36864       -0.23362
    'measurement'           0.06288      0.034091      1.8445    46    0.071555     -0.0057407
    'drug'                 -0.22681       0.15573     -1.4564    46     0.15206       -0.54027
    'ketLvl'             9.1869e-06    7.4296e-06      1.2365    46     0.22254    -5.7682e-06
    'sex'                 -0.024096      0.036779    -0.65516    46     0.51563      -0.098128
    'age'                  0.025641      0.024037      1.0668    46     0.29165      -0.022742
    'drugOrder'            0.046542      0.034086      1.3654    46     0.17876       -0.02207
```

```
    'fiveDASC'            0.0092154      0.017353     0.53106    46     0.59794      -0.025714


    Upper
       0.08838
        0.1315
      0.086654
    2.4142e-05
      0.049936
      0.074024
       0.11515
      0.044145

Random effects covariance parameters (95% CIs):
Group: subject (27 Levels)
    Name1                Name2                Type         Estimate     Lower
    '(Intercept)'        '(Intercept)'        'std'        0.0004557    5.9088e-290


    Upper
    3.5145e+282

Group: Error
    Name             Estimate    Lower       Upper
    'Res Std'        0.12174     0.099224    0.14937


 Contrast: fb failure vs fb neutral      ROI: thalamus 5D-ASC subscore: asc_aed

lme =


Linear mixed-effects model fit by REML

Model information:
    Number of observations              54
    Fixed effects coefficients           8
    Random effects coefficients         27
    Covariance parameters                2

Formula:
    ROI ~ 1 + measurement + drug + ketLvl + sex + age + drugOrder + fiveDASC + (1 | subject)

Model fit statistics:
    AIC        BIC       LogLikelihood    Deviance
    -2.9975    15.289    11.499           -22.998

Fixed effects coefficients (95% CIs):
    Name                 Estimate      SE             tStat       DF    pValue     Lower
    '(Intercept)'         -0.068918      0.079115     -0.87111    46    0.38822       -0.22817
    'measurement'          0.053187      0.033545       1.5855    46    0.11969      -0.014336
    'drug'                 -0.25938       0.15571      -1.6658    46    0.10255        -0.5728
    'ketLvl'             1.0759e-05    7.4308e-06       1.4479    46    0.15444    -4.1987e-06
    'sex'                 -0.024317      0.036475     -0.66667    46    0.50831      -0.097737
    'age'                  0.030386      0.023918       1.2704    46    0.21032      -0.017759
    'drugOrder'             0.05469      0.034316       1.5937    46    0.11784      -0.014383
    'fiveDASC'            -0.017506      0.017459      -1.0027    46    0.32126      -0.052649


    Upper
      0.090333
       0.12071
      0.054043
    2.5716e-05
      0.049103
      0.078531
       0.12376
      0.017638

Random effects covariance parameters (95% CIs):
Group: subject (27 Levels)
    Name1                Name2                Type         Estimate      Lower
    '(Intercept)'        '(Intercept)'        'std'        0.00053777    4.7666e-275


    Upper
    6.0671e+267
```

```
Group: Error
    Name             Estimate    Lower       Upper  
    'Res Std'        0.1208      0.098443    0.14824


 Contrast: fb failure vs fb neutral      ROI: thalamus 5D-ASC subscore: asc_vis

lme = 


Linear mixed-effects model fit by REML

Model information:
    Number of observations              54
    Fixed effects coefficients           8
    Random effects coefficients         27
    Covariance parameters                2

Formula:
    ROI ~ 1 + measurement + drug + ketLvl + sex + age + drugOrder + fiveDASC + (1 | subject)

Model fit statistics:
    AIC        BIC       LogLikelihood    Deviance
    -2.0444    16.242    11.022           -22.044 

Fixed effects coefficients (95% CIs):
    Name                 Estimate      SE            tStat       DF    pValue      Lower      
    '(Intercept)'         -0.070018      0.080415    -0.87071    46     0.38843       -0.23189
    'measurement'          0.060051      0.035273      1.7025    46    0.095419       -0.01095
    'drug'                 -0.23451        0.1555     -1.5081    46     0.13836       -0.54751
    'ketLvl'             9.5576e-06    7.4179e-06      1.2885    46     0.20403    -5.3738e-06
    'sex'                 -0.023416      0.036907    -0.63446    46     0.52892      -0.097707
    'age'                  0.026871      0.024082      1.1158    46     0.27029      -0.021603
    'drugOrder'             0.04763      0.034508      1.3802    46     0.17418      -0.021832
    'fiveDASC'            0.0016628      0.017939    0.092692    46     0.92655      -0.034447


    Upper     
      0.091849
       0.13105
       0.07849
    2.4489e-05
      0.050874
      0.075345
       0.11709
      0.037772

Random effects covariance parameters (95% CIs):
Group: subject (27 Levels)
    Name1                Name2                Type         Estimate      Lower        
    '(Intercept)'        '(Intercept)'        'std'        0.00023242    1.0675e-265


    Upper     
    5.0601e+257

Group: Error
    Name             Estimate    Lower       Upper  
    'Res Std'        0.1221      0.099537    0.14979


 Contrast: fb failure vs fb neutral      ROI: thalamus 5D-ASC subscore: asc_aud

lme = 


Linear mixed-effects model fit by REML

Model information:
    Number of observations              54
    Fixed effects coefficients           8
    Random effects coefficients         27
    Covariance parameters                2

Formula:
    ROI ~ 1 + measurement + drug + ketLvl + sex + age + drugOrder + fiveDASC + (1 | subject)

Model fit statistics:
```

```
    AIC        BIC       LogLikelihood    Deviance
    -2.0574    16.229    11.029           -22.057

Fixed effects coefficients (95% CIs):
    Name                 Estimate      SE            tStat       DF    pValue      Lower
    '(Intercept)'         -0.072689      0.080532    -0.90262    46     0.37143      -0.23479
    'measurement'          0.061221      0.034004      1.8004    46    0.078357    -0.0072258
    'drug'                 -0.22875       0.15633     -1.4632    46     0.15021      -0.54343
    'ketLvl'             9.2785e-06    7.4592e-06      1.2439    46     0.21984    -5.736e-06
    'sex'                 -0.023121      0.036811    -0.62812    46     0.53303     -0.097217
    'age'                  0.027719       0.02399      1.1554    46     0.25389     -0.020571
    'drugOrder'            0.048252      0.034015      1.4186    46     0.16277     -0.020216
    'fiveDASC'             0.005768      0.017114     0.33704    46     0.73762      -0.02868


    Upper
      0.089413
       0.12967
      0.085936
    2.4293e-05
      0.050974
      0.076009
       0.11672
      0.040216

Random effects covariance parameters (95% CIs):
Group: subject (27 Levels)
    Name1                Name2                Type         Estimate      Lower
    '(Intercept)'        '(Intercept)'        'std'        0.00024194    7.3726e-281


    Upper
    7.9392e+272

Group: Error
    Name             Estimate    Lower       Upper
    'Res Std'        0.12197     0.099423    0.14962


 Contrast: fb failure vs fb neutral      ROI: thalamus 5D-ASC subscore: asc_vig

lme =


Linear mixed-effects model fit by REML

Model information:
    Number of observations              54
    Fixed effects coefficients           8
    Random effects coefficients         27
    Covariance parameters                2

Formula:
    ROI ~ 1 + measurement + drug + ketLvl + sex + age + drugOrder + fiveDASC + (1 | subject)

Model fit statistics:
    AIC        BIC       LogLikelihood    Deviance
    -2.0311    16.255    11.016           -22.031

Fixed effects coefficients (95% CIs):
    Name                 Estimate      SE            tStat       DF    pValue     Lower
    '(Intercept)'         -0.068638      0.080014    -0.85783    46    0.39543         -0.2297
    'measurement'          0.057342      0.034622      1.6562    46    0.10448       -0.012348
    'drug'                 -0.23536       0.15542     -1.5144    46    0.13677         -0.5482
    'ketLvl'             9.5944e-06    7.4143e-06       1.294    46    0.20211     -5.3299e-06
    'sex'                 -0.023254      0.036842    -0.63118    46    0.53105       -0.097413
    'age'                  0.026958      0.023956      1.1253    46    0.26629       -0.021263
    'drugOrder'            0.049434      0.034777      1.4214    46    0.16193       -0.020569
    'fiveDASC'           -0.0031848      0.017601    -0.18094    46    0.85721       -0.038614


    Upper
      0.092421
       0.12703
      0.077476
    2.4519e-05
      0.050905
      0.075179
```

```
       0.11944
      0.032245

Random effects covariance parameters (95% CIs):
Group: subject (27 Levels)
    Name1                Name2                Type         Estimate      Lower     
    '(Intercept)'        '(Intercept)'        'std'        0.00028784    2.1878e-277


    Upper     
    3.7868e+269

Group: Error
    Name             Estimate    Lower       Upper  
    'Res Std'        0.12207     0.099509    0.14975


 Contrast: fb failure vs fb neutral      ROI: thalamus 5D-ASC subscore: sum

lme = 


Linear mixed-effects model fit by REML

Model information:
    Number of observations              54
    Fixed effects coefficients           8
    Random effects coefficients         27
    Covariance parameters                2

Formula:
    ROI ~ 1 + measurement + drug + ketLvl + sex + age + drugOrder + fiveDASC + (1 | subject)

Model fit statistics:
    AIC       BIC       LogLikelihood    Deviance
    -2.021    16.265    11.01            -22.021 

Fixed effects coefficients (95% CIs):
    Name                 Estimate      SE            tStat       DF    pValue      Lower      
    '(Intercept)'         -0.069358      0.080266    -0.86411    46     0.39201       -0.23092
    'measurement'           0.05918      0.034829      1.6992    46    0.096038      -0.010926
    'drug'                  -0.2346       0.15634     -1.5006    46     0.14029       -0.54929
    'ketLvl'             9.5605e-06    7.4585e-06      1.2818    46     0.20632    -5.4526e-06
    'sex'                 -0.023245      0.036866    -0.63052    46     0.53148      -0.097452
    'age'                  0.027065      0.024052      1.1253    46     0.26631      -0.021349
    'drugOrder'            0.048047      0.034497      1.3928    46     0.17038      -0.021392
    'fiveDASC'           0.00032688      0.017805    0.018359    46     0.98543      -0.035513


    Upper     
      0.092209
       0.12929
      0.080091
    2.4574e-05
      0.050963
      0.075479
       0.11749
      0.036166

Random effects covariance parameters (95% CIs):
Group: subject (27 Levels)
    Name1                Name2                Type         Estimate      Lower     
    '(Intercept)'        '(Intercept)'        'std'        0.00025322    1.9877e-277


    Upper     
    3.2257e+269

Group: Error
    Name             Estimate    Lower       Upper 
    'Res Std'        0.12212     0.099545    0.1498


 Contrast: fb failure vs fb neutral      ROI: amygdala 5D-ASC subscore: asc_obn

lme = 
```

```
Linear mixed-effects model fit by REML

Model information:
    Number of observations              54
    Fixed effects coefficients           8
    Random effects coefficients         27
    Covariance parameters                2

Formula:
    ROI ~ 1 + measurement + drug + ketLvl + sex + age + drugOrder + fiveDASC + (1 | subject)

Model fit statistics:
    AIC       BIC       LogLikelihood    Deviance
    61.781    80.068    -20.891          41.781

Fixed effects coefficients (95% CIs):
    Name                   Estimate      SE            tStat       DF    pValue      Lower
    '(Intercept)'            -0.12992       0.16479    -0.78843    46     0.43449       -0.46162
    'measurement'            0.011921      0.062048     0.19213    46     0.84848       -0.11297
    'drug'                   -0.13536       0.30453    -0.44449    46     0.65877       -0.74834
    'ketLvl'               4.0774e-06    1.4578e-05     0.27969    46     0.78097    -2.5267e-05
    'sex'                     -0.1342      0.081417     -1.6483    46     0.10611       -0.29808
    'age'                    0.096053      0.053716      1.7882    46    0.080336      -0.012071
    'drugOrder'               0.12253      0.076255      1.6069    46     0.11492      -0.030959
    'fiveDASC'               0.047913      0.034413      1.3923    46     0.17053      -0.021358


    Upper
       0.20178
       0.13682
       0.47762
    3.3422e-05
      0.029685
       0.20418
       0.27603
       0.11718

Random effects covariance parameters (95% CIs):
Group: subject (27 Levels)
    Name1                    Name2                    Type         Estimate    Lower       Upper
    '(Intercept)'            '(Intercept)'            'std'        0.11348     0.039097    0.32939

Group: Error
    Name             Estimate    Lower      Upper
    'Res Std'        0.22038     0.16428    0.29564


 Contrast: fb failure vs fb neutral      ROI: amygdala 5D-ASC subscore: asc_aed

lme =


Linear mixed-effects model fit by REML

Model information:
    Number of observations              54
    Fixed effects coefficients           8
    Random effects coefficients         27
    Covariance parameters                2

Formula:
    ROI ~ 1 + measurement + drug + ketLvl + sex + age + drugOrder + fiveDASC + (1 | subject)

Model fit statistics:
    AIC       BIC       LogLikelihood    Deviance
    63.457    81.743    -21.729          43.457

Fixed effects coefficients (95% CIs):
    Name                   Estimate      SE            tStat       DF    pValue     Lower
    '(Intercept)'            -0.11254       0.16999    -0.66202    46    0.51126       -0.45472
    'measurement'           -0.006181      0.060298    -0.10251    46     0.9188       -0.12756
    'drug'                   -0.19189       0.30712    -0.62481    46    0.53518       -0.81009
    'ketLvl'               6.8036e-06    1.4719e-05     0.46225    46    0.64608    -2.2823e-05
    'sex'                    -0.13099      0.086058     -1.5222    46    0.13482       -0.30422
    'age'                     0.10248      0.057133      1.7937    46    0.07943      -0.012521
    'drugOrder'               0.12918      0.081797      1.5793    46    0.12112      -0.035465
    'fiveDASC'              0.0049541      0.035366     0.14008    46    0.88921      -0.066234
```

```
    Upper
       0.22964
       0.11519
       0.42631
    3.6431e-05
      0.042232
       0.21748
       0.29383
      0.076142

Random effects covariance parameters (95% CIs):
Group: subject (27 Levels)
    Name1                Name2                Type         Estimate    Lower       Upper  
    '(Intercept)'        '(Intercept)'        'std'        0.13641     0.063709    0.29209

Group: Error
    Name             Estimate    Lower      Upper  
    'Res Std'        0.2159      0.16202    0.28769


 Contrast: fb failure vs fb neutral      ROI: amygdala 5D-ASC subscore: asc_vis

lme = 


Linear mixed-effects model fit by REML

Model information:
    Number of observations              54
    Fixed effects coefficients           8
    Random effects coefficients         27
    Covariance parameters                2

Formula:
    ROI ~ 1 + measurement + drug + ketLvl + sex + age + drugOrder + fiveDASC + (1 | subject)

Model fit statistics:
    AIC       BIC       LogLikelihood    Deviance
    63.443    81.729    -21.721          43.443  

Fixed effects coefficients (95% CIs):
    Name                Estimate      SE            tStat       DF    pValue      Lower     
    '(Intercept)'         -0.10956       0.17111     -0.6403    46     0.52515      -0.45399
    'measurement'        -0.011681      0.062984    -0.18546    46     0.85368      -0.13846
    'drug'                -0.20107       0.30387     -0.6617    46     0.51147      -0.81274
    'ketLvl'            7.2416e-06    1.4562e-05      0.4973    46     0.62134    -2.207e-05
    'sex'                 -0.13067      0.086493     -1.5107    46      0.1377      -0.30477
    'age'                  0.10426      0.057207      1.8225    46    0.074876      -0.01089
    'drugOrder'            0.13295      0.081836      1.6245    46     0.11109     -0.031781
    'fiveDASC'          -0.0061184      0.035387     -0.1729    46     0.86349     -0.077348


    Upper
       0.23487
        0.1151
       0.41059
    3.6553e-05
      0.043435
       0.21942
       0.29768
      0.065111

Random effects covariance parameters (95% CIs):
Group: subject (27 Levels)
    Name1                Name2                Type         Estimate    Lower       Upper 
    '(Intercept)'        '(Intercept)'        'std'        0.13853     0.066125    0.2902

Group: Error
    Name             Estimate    Lower      Upper 
    'Res Std'        0.21494     0.16131    0.2864


 Contrast: fb failure vs fb neutral      ROI: amygdala 5D-ASC subscore: asc_aud

lme = 
```

```
Linear mixed-effects model fit by REML

Model information:
    Number of observations             54
    Fixed effects coefficients          8
    Random effects coefficients        27
    Covariance parameters               2

Formula:
    ROI ~ 1 + measurement + drug + ketLvl + sex + age + drugOrder + fiveDASC + (1 | subject)

Model fit statistics:
    AIC       BIC       LogLikelihood    Deviance
    62.816    81.102    -21.408          42.816

Fixed effects coefficients (95% CIs):
    Name                    Estimate     SE            tStat       DF    pValue      Lower
    '(Intercept)'           -0.094859       0.1719     -0.55182    46     0.58374       -0.44088
    'measurement'           -0.018894     0.058798     -0.32135    46      0.7494       -0.13725
    'drug'                   -0.24559      0.30177     -0.81381    46     0.41994       -0.85302
    'ketLvl'                9.401e-06   1.4469e-05      0.64971    46     0.51911    -1.9725e-05
    'sex'                      -0.133     0.087462      -1.5206    46     0.13519       -0.30905
    'age'                     0.10009     0.057917       1.7281    46    0.090681      -0.016495
    'drugOrder'               0.13061     0.082256       1.5878    46     0.11917      -0.034963
    'fiveDASC'              -0.029533     0.034617     -0.85311    46     0.39802      -0.099214


    Upper
       0.25116
      0.099459
       0.36185
    3.8527e-05
      0.043053
       0.21667
       0.29618
      0.040149

Random effects covariance parameters (95% CIs):
Group: subject (27 Levels)
    Name1                   Name2                   Type          Estimate    Lower       Upper
    '(Intercept)'           '(Intercept)'           'std'         0.14708     0.075093    0.28807

Group: Error
    Name               Estimate    Lower      Upper
    'Res Std'          0.20919     0.15676    0.27915


 Contrast: fb failure vs fb neutral      ROI: amygdala 5D-ASC subscore: asc_vig

lme =


Linear mixed-effects model fit by REML

Model information:
    Number of observations             54
    Fixed effects coefficients          8
    Random effects coefficients        27
    Covariance parameters               2

Formula:
    ROI ~ 1 + measurement + drug + ketLvl + sex + age + drugOrder + fiveDASC + (1 | subject)

Model fit statistics:
    AIC       BIC       LogLikelihood    Deviance
    62.738    81.024    -21.369          42.738

Fixed effects coefficients (95% CIs):
    Name                    Estimate      SE            tStat       DF    pValue      Lower
    '(Intercept)'             -0.11829      0.16737     -0.70679    46     0.48326       -0.45518
    'measurement'            0.0084631     0.062867      0.13462    46      0.8935       -0.11808
    'drug'                    -0.19047      0.30479     -0.62493    46     0.53511       -0.80398
    'ketLvl'                6.7607e-06   1.4598e-05      0.46312    46     0.64546    -2.2624e-05
    'sex'                     -0.13075      0.08364      -1.5633    46     0.12484       -0.29911
    'age'                      0.10491     0.055055       1.9055    46    0.062974     -0.0059117
    'drugOrder'                0.11831     0.079619       1.4859    46     0.14412      -0.041957
    'fiveDASC'                0.031452     0.035898      0.87615    46      0.3855      -0.040807
```

```
    Upper
       0.2186
      0.13501
      0.42304
   3.6145e-05
     0.037605
      0.21573
      0.27857
      0.10371

Random effects covariance parameters (95% CIs):
Group: subject (27 Levels)
    Name1                Name2                Type         Estimate    Lower       Upper  
    '(Intercept)'        '(Intercept)'        'std'        0.12427     0.049709    0.31068

Group: Error
    Name             Estimate    Lower      Upper  
    'Res Std'        0.21902     0.16357    0.29327


 Contrast: fb failure vs fb neutral      ROI: amygdala 5D-ASC subscore: sum

lme = 


Linear mixed-effects model fit by REML

Model information:
    Number of observations              54
    Fixed effects coefficients           8
    Random effects coefficients         27
    Covariance parameters                2

Formula:
    ROI ~ 1 + measurement + drug + ketLvl + sex + age + drugOrder + fiveDASC + (1 | subject)

Model fit statistics:
    AIC       BIC     LogLikelihood    Deviance
    62.814    81.1    -21.407          42.814  

Fixed effects coefficients (95% CIs):
    Name                Estimate      SE           tStat       DF    pValue      Lower      
    '(Intercept)'          -0.1238      0.16801    -0.73684    46     0.46496       -0.46198
    'measurement'        0.0084857     0.063259     0.13414    46     0.89388       -0.11885
    'drug'                -0.16323      0.30611    -0.53325    46     0.59643       -0.77941
    'ketLvl'            5.4316e-06    1.466e-05      0.3705    46     0.71271    -2.4077e-05
    'sex'                 -0.13224     0.083812     -1.5778    46     0.12146       -0.30095
    'age'                 0.099825     0.055318      1.8046    46    0.077691      -0.011524
    'drugOrder'             0.1218     0.079223      1.5374    46     0.13105      -0.037672
    'fiveDASC'            0.029703     0.036089     0.82304    46     0.41473      -0.042941


    Upper     
      0.21439
      0.13582
      0.45294
   3.4941e-05
     0.036463
      0.21117
      0.28126
      0.10235

Random effects covariance parameters (95% CIs):
Group: subject (27 Levels)
    Name1                Name2                Type         Estimate    Lower       Upper  
    '(Intercept)'        '(Intercept)'        'std'        0.1248      0.050245    0.30999

Group: Error
    Name             Estimate    Lower      Upper  
    'Res Std'        0.21905     0.16358    0.29333
```
