# Supplementary material for: Subacute effects of ketamine on neural correlates of reward processing
Source: Transl Psychiatry. 2026 Sep 25;16:521. doi: 10.1038/s41398-026-04485-6 (PMC13639076; doi:10.1038/s41398-026-04485-6)
Supplement: Supplementary file 2 [file 41398_2026_4485_MOESM1_ESM.docx]

**Supplement**

**Supplementary Tables:**

**Table S1. Plasma concentrations of ketamine (n = 28).** Mean, standard deviation, minimum, and maximum plasma levels of ketamine at each time point following a 40-minute intravenous ketamine infusion (0.5 mg/kg, infusion start = 0 min).

| **Time (min)** | **Ketamine_Mean (ng/ml)** | **Ketamine_SD (ng/ml)** | **Ketamine_Min (ng/ml)** | **Ketamine_Max (ng/ml)** |
| --- | --- | --- | --- | --- |
| **0** | 0.00 | 0.00 | 0.00 | 0.00 |
| **5** | 39.08 | 31.01 | 8.08 | 166.7 |
| **10** | 73.05 | 40.62 | 32.43 | 186.44 |
| **20** | 115.01 | 52.48 | 58.55 | 285.85 |
| **40** | 165.19 | 41.81 | 111.26 | 297.0 |
| **100** | 67.18 | 12.09 | 46.21 | 91.27 |
| **360** | 17.19 | 4.61 | 8.20 | 26.05 |

**Table S2. Plasma concentrations of norketamine (n = 28).** Mean, standard deviation, minimum, and maximum plasma levels of norketamine at each time point following a 40-minute intravenous ketamine infusion (0.5 mg/kg, infusion start = 0 min).

| **Time (min)** | **Norketamine_Mean (ng/ml)** | **Norketamine_SD (ng/ml)** | **Norketamine_Min (ng/ml)** | **Norketamine_Max (ng/ml)** |
| --- | --- | --- | --- | --- |
| **0** | 0.00 | 0.00 | 0.00 | 0.00 |
| **5** | 0.00 | 0.00 | 0.00 | 0.00 |
| **10** | 1.93 | 2.49 | 0.00 | 6.58 |
| **20** | 15.4 | 9.89 | 6.84 | 49.35 |
| **40** | 45.63 | 18.68 | 24.04 | 101.88 |
| **100** | 70.85 | 17.43 | 41.79 | 102.78 |
| **360** | 44.4 | 13.06 | 26.83 | 75.12 |

**Table S3. Descriptive 5D-ASC dimensional scores for placebo and ketamine conditions (n = 27). Mean summed scores and standard deviation are shown for the five canonical dimensions of the 5-Dimensional Altered States of Consciousness Rating Scale (5D-ASC): Oceanic Boundlessness (OBN), Anxious Ego Dissolution (AED), Visionary Restructuralization (VIS), Auditory Alterations (AUD), and Reduction of Vigilance (VIG), as well as the total sum score. Maximum possible scores for each dimension are additionally provided for contextual interpretation.**

| **Mean ± Std** | **OBN** | **AED** | **VIS** | **AUD** | **VIG** | **Sum** |
| --- | --- | --- | --- | --- | --- | --- |
| **Placebo** | 77 ± 163 | 31 ± 59 | 24 ± 50 | 2 ± 12 | 116 ± 174 | 252 ± 406 |
| **Ketamine** | 1043 ± 592 | 532 ±323 | 472 ± 321 | 200 ±184 | 722 ± 219 | 2970 ± 1363 |
| **Maxima** | 2700 | 2100 | 1800 | 1500 | 1300 | 9400 |

**Table S4. Whole-brain results from the flexible factorial model (F-tests) for substance effects on the MID task contrasts.** Statistical maps were thresholded at p < 0.001 (uncorrected) at the voxel level. For each contrast, the global maximum peak is reported. No clusters survived cluster-level family-wise error correction (pFWE < 0.05). Coordinates are reported in MNI space.

| **Contrast** | **x** | **y** | **z** | **F** | **k** | **pFWE** |
| --- | --- | --- | --- | --- | --- | --- |
| **WinCue > NeutralCue** | 24 | -40 | -52 | 20.40 | 7 | 0.944 |
| **LossCue > NeutralCue** | -9 | -31 | -16 | 22.94 | 12 | 0.853 |
| **SuccessFB > NeutralFB** | 9 | 20 | 62 | 18.26 | 13 | 0.833 |
| **FailureFB > NeutralFB** | -57 | -40 | 32 | 18.93 | 15 | 0.790 |
| **Congruent > Incongruent** | -9 | 17 | -25 | 21.50 | 5 | 0.965 |

**Table S5. Whole-brain paired t-test results comparing ketamine and placebo sessions for the MID task contrasts.** Statistical maps were thresholded at p < 0.001 (uncorrected) at the voxel level. No clusters survived cluster-level family-wise error correction (pFWE < 0.05). Coordinates are reported in MNI space.

| **Contrast** | **x** | **y** | **z** | **T** | **k** | **pFWE** |
| --- | --- | --- | --- | --- | --- | --- |
| **WinCue > NeutralCue** | 3 | 8 | 2 | 5.13 | 26 | 0.586 |
|  | -15 | 5 | 50 | 4.49 | 15 | 0.795 |
|  | -15 | -91 | -10 | 3.67 | 5 | 0.957 |
| **LossCue > NeutralCue** | -15 | 5 | 53 | 3.47 | 1 | 0.993 |
|  | -18 | 5 | 62 | 3.47 | 2 | 0.987 |
| **SuccessFB > NeutralFB** | 63 | -58 | -16 | 5.23 | 34 | 0.467 |
|  | -42 | -82 | 29 | 3.92 | 8 | 0.913 |
|  | 57 | -31 | -28 | 3.86 | 9 | 0.898 |
|  | 24 | 29 | -10 | 3.81 | 4 | 0.964 |
|  | -27 | -94 | 32 | 3.60 | 2 | 0.982 |
| **FailureFB > NeutralFB** | -12 | 41 | -19 | 3.48 | 1 | 0.988 |
| **Congruent > Incongruent** | 27 | -16 | -28 | 3.92 | 14 | 0.811 |
|  | 21 | 2 | 5 | 3.89 | 11 | 0.862 |
|  | -57 | -16 | -31 | 3.87 | 18 | 0.739 |
|  | 48 | -61 | 44 | 3.84 | 15 | 0.793 |
|  | 57 | -52 | 26 | 3.67 | 2 | 0.980 |
|  | -33 | -97 | 17 | 3.64 | 5 | 0.949 |
|  | 3 | -61 | -49 | 3.63 | 3 | 0.971 |
|  | 51 | -55 | 14 | 3.61 | 4 | 0.961 |
|  | -51 | -58 | 53 | 3.52 | 1 | 0.988 |
|  | 27 | -25 | -31 | 3.50 | 1 | 0.988 |
|  | -9 | -82 | -43 | 3.49 | 1 | 0.988 |

**Table S6. Linear mixed-effects results for ROI analyses across MID task contrasts (n=27). For each ROI, first-level contrast estimates (β values) were analyzed using LME models including drug condition (ketamine vs. placebo), individual ketamine exposure (ketLvl; area under the curve of ketamine plasma concentrations up to half the task duration), subjective drug experience (5D-ASC scores), age, sex, measurement session, and drug order as fixed effects. Reported values represent fixed-effect estimates (β), standard errors (SE), t statistics, and uncorrected p values, rounded to three decimal places. No effects survived FDR correction for multiple comparisons.**

| **Contrast** | **ROI** | **Predictor** | **β** | **SE** | **t** | **p** |
| --- | --- | --- | --- | --- | --- | --- |
| **WinCue > NeutralCue** | **Striatum** | measurement | -0.010 | 0.015 | -0.655 | 0.515 |
|  |  | drug | 0.031 | 0.078 | 0.402 | 0.690 |
|  |  | ketLvl | 0.000 | 0.000 | -0.431 | 0.669 |
|  |  | sex | 0.026 | 0.033 | 0.782 | 0.438 |
|  |  | age | -0.018 | 0.022 | -0.822 | 0.415 |
|  |  | drugOrder | 0.005 | 0.032 | 0.147 | 0.884 |
|  |  | fiveDASC | -0.009 | 0.010 | -0.956 | 0.344 |
| **WinCue > NeutralCue** | **Nucleus accumbens** | measurement | -0.019 | 0.028 | -0.661 | 0.512 |
|  |  | drug | 0.173 | 0.135 | 1.283 | 0.206 |
|  |  | ketLvl | 0.000 | 0.000 | -1.390 | 0.171 |
|  |  | sex | -0.052 | 0.036 | -1.459 | 0.151 |
|  |  | age | -0.032 | 0.024 | -1.371 | 0.177 |
|  |  | drugOrder | 0.073 | 0.034 | 2.176 | 0.035* |
|  |  | fiveDASC | -0.003 | 0.016 | -0.216 | 0.830 |
| **WinCue > NeutralCue** | **Insula** | measurement | 0.017 | 0.036 | 0.491 | 0.626 |
|  |  | drug | 0.085 | 0.186 | 0.456 | 0.651 |
|  |  | ketLvl | 0.000 | 0.000 | -0.383 | 0.704 |
|  |  | sex | 0.031 | 0.072 | 0.431 | 0.669 |
|  |  | age | -0.038 | 0.048 | -0.796 | 0.430 |
|  |  | drugOrder | -0.012 | 0.069 | -0.176 | 0.861 |
|  |  | fiveDASC | 0.003 | 0.023 | 0.134 | 0.894 |
| **WinCue > NeutralCue** | **Thalamus** | measurement | -0.006 | 0.023 | -0.269 | 0.789 |
|  |  | drug | 0.054 | 0.113 | 0.476 | 0.636 |
|  |  | ketLvl | 0.000 | 0.000 | -0.294 | 0.770 |
|  |  | sex | 0.019 | 0.033 | 0.579 | 0.566 |
|  |  | age | -0.014 | 0.022 | -0.641 | 0.524 |
|  |  | drugOrder | 0.019 | 0.032 | 0.591 | 0.557 |
|  |  | fiveDASC | 0.001 | 0.013 | 0.068 | 0.946 |
| **WinCue > NeutralCue** | **Amygdala** | measurement | -0.025 | 0.035 | -0.717 | 0.477 |
|  |  | drug | 0.229 | 0.183 | 1.254 | 0.216 |
|  |  | ketLvl | 0.000 | 0.000 | -1.585 | 0.120 |
|  |  | sex | 0.060 | 0.067 | 0.902 | 0.372 |
|  |  | age | -0.063 | 0.045 | -1.407 | 0.166 |
|  |  | drugOrder | -0.078 | 0.064 | -1.223 | 0.228 |
|  |  | fiveDASC | -0.041 | 0.023 | -1.823 | 0.075 |

| **Contrast** | **ROI** | **Predictor** | **β** | **SE** | **t** | **p** |
| --- | --- | --- | --- | --- | --- | --- |
| **LossCue > NeutralCue** | **Striatum** | measurement | -0.008 | 0.019 | -0.397 | 0.694 |
|  |  | drug | -0.017 | 0.097 | -0.172 | 0.864 |
|  |  | ketLvl | 0.000 | 0.000 | 0.037 | 0.971 |
|  |  | sex | 0.029 | 0.030 | 0.976 | 0.334 |
|  |  | age | -0.020 | 0.020 | -1.021 | 0.312 |
|  |  | drugOrder | 0.019 | 0.028 | 0.682 | 0.499 |
|  |  | fiveDASC | 0.001 | 0.012 | 0.111 | 0.912 |
| **LossCue > NeutralCue** | **Nucleus accumbens** | measurement | -0.000 | 0.028 | -0.004 | 0.997 |
|  |  | drug | 0.229 | 0.140 | 1.634 | 0.109 |
|  |  | ketLvl | 0.000 | 0.000 | -1.817 | 0.076 |
|  |  | sex | -0.019 | 0.041 | -0.476 | 0.636 |
|  |  | age | -0.047 | 0.027 | -1.718 | 0.092 |
|  |  | drugOrder | 0.038 | 0.039 | 0.969 | 0.338 |
|  |  | fiveDASC | 0.036 | 0.017 | 2.136 | 0.038* |
| **LossCue > NeutralCue** | **Insula** | measurement | 0.033 | 0.036 | 0.917 | 0.364 |
|  |  | drug | 0.136 | 0.185 | 0.738 | 0.464 |
|  |  | ketLvl | 0.000 | 0.000 | -0.757 | 0.453 |
|  |  | sex | 0.042 | 0.065 | 0.638 | 0.527 |
|  |  | age | -0.041 | 0.043 | -0.934 | 0.355 |
|  |  | drugOrder | -0.006 | 0.062 | -0.090 | 0.929 |
|  |  | fiveDASC | 0.012 | 0.023 | 0.541 | 0.591 |
| **LossCue > NeutralCue** | **Thalamus** | measurement | 0.018 | 0.019 | 0.973 | 0.335 |
|  |  | drug | 0.194 | 0.095 | 2.041 | 0.047* |
|  |  | ketLvl | 0.000 | 0.000 | -1.875 | 0.067 |
|  |  | sex | 0.040 | 0.029 | 1.376 | 0.175 |
|  |  | age | -0.011 | 0.019 | -0.544 | 0.589 |
|  |  | drugOrder | 0.019 | 0.028 | 0.694 | 0.491 |
|  |  | fiveDASC | 0.013 | 0.011 | 1.169 | 0.248 |
| **LossCue > NeutralCue** | **Amygdala** | measurement | -0.057 | 0.035 | -1.613 | 0.114 |
|  |  | drug | 0.182 | 0.175 | 1.040 | 0.304 |
|  |  | ketLvl | 0.000 | 0.000 | -1.385 | 0.173 |
|  |  | sex | 0.020 | 0.053 | 0.380 | 0.706 |
|  |  | age | -0.052 | 0.035 | -1.492 | 0.143 |
|  |  | drugOrder | -0.073 | 0.050 | -1.459 | 0.151 |
|  |  | fiveDASC | -0.035 | 0.021 | -1.680 | 0.100 |

| **Contrast** | **ROI** | **Predictor** | **β** | **SE** | **t** | **p** |
| --- | --- | --- | --- | --- | --- | --- |
| **SuccessFB > NeutralFB** | **Striatum** | measurement | 0.048 | 0.038 | 1.245 | 0.219 |
|  |  | drug | 0.096 | 0.190 | 0.504 | 0.617 |
|  |  | ketLvl | 0.000 | 0.000 | -0.765 | 0.448 |
|  |  | sex | -0.001 | 0.055 | -0.018 | 0.985 |
|  |  | age | 0.027 | 0.036 | 0.731 | 0.468 |
|  |  | drugOrder | 0.043 | 0.052 | 0.831 | 0.410 |
|  |  | fiveDASC | 0.020 | 0.023 | 0.880 | 0.383 |
| **SuccessFB > NeutralFB** | **Nucleus accumbens** | measurement | 0.064 | 0.053 | 1.220 | 0.229 |
|  |  | drug | 0.146 | 0.237 | 0.615 | 0.542 |
|  |  | ketLvl | 0.000 | 0.000 | -0.430 | 0.669 |
|  |  | sex | 0.051 | 0.056 | 0.905 | 0.370 |
|  |  | age | 0.047 | 0.036 | 1.295 | 0.202 |
|  |  | drugOrder | -0.005 | 0.052 | -0.090 | 0.928 |
|  |  | fiveDASC | -0.011 | 0.027 | -0.422 | 0.675 |
| **SuccessFB > NeutralFB** | **Insula** | measurement | 0.117 | 0.076 | 1.554 | 0.127 |
|  |  | drug | 0.020 | 0.383 | 0.053 | 0.958 |
|  |  | ketLvl | 0.000 | 0.000 | -0.150 | 0.882 |
|  |  | sex | 0.050 | 0.123 | 0.403 | 0.689 |
|  |  | age | 0.068 | 0.082 | 0.826 | 0.413 |
|  |  | drugOrder | 0.186 | 0.118 | 1.583 | 0.120 |
|  |  | fiveDASC | 0.042 | 0.046 | 0.909 | 0.368 |
| **SuccessFB > NeutralFB** | **Thalamus** | measurement | 0.011 | 0.034 | 0.315 | 0.754 |
|  |  | drug | -0.175 | 0.157 | -1.113 | 0.271 |
|  |  | ketLvl | 0.000 | 0.000 | 0.916 | 0.364 |
|  |  | sex | -0.058 | 0.039 | -1.467 | 0.149 |
|  |  | age | 0.026 | 0.026 | 1.003 | 0.321 |
|  |  | drugOrder | 0.027 | 0.037 | 0.740 | 0.463 |
|  |  | fiveDASC | 0.012 | 0.018 | 0.655 | 0.516 |
| **SuccessFB > NeutralFB** | **Amygdala** | measurement | -0.046 | 0.062 | -0.732 | 0.468 |
|  |  | drug | -0.210 | 0.301 | -0.698 | 0.489 |
|  |  | ketLvl | 0.000 | 0.000 | 0.538 | 0.593 |
|  |  | sex | -0.073 | 0.082 | -0.884 | 0.381 |
|  |  | age | 0.029 | 0.054 | 0.534 | 0.596 |
|  |  | drugOrder | 0.155 | 0.078 | 1.991 | 0.052 |
|  |  | fiveDASC | 0.010 | 0.035 | 0.291 | 0.772 |

| **Contrast** | **ROI** | **Predictor** | **β** | **SE** | **t** | **p** |
| --- | --- | --- | --- | --- | --- | --- |
| **FailureFB > NeutralFB** | **Striatum** | measurement | 0.072 | 0.037 | 1.978 | 0.054 |
|  |  | drug | 0.150 | 0.182 | 0.822 | 0.415 |
|  |  | ketLvl | 0.000 | 0.000 | -0.961 | 0.342 |
|  |  | sex | 0.019 | 0.055 | 0.346 | 0.731 |
|  |  | age | 0.054 | 0.036 | 1.480 | 0.146 |
|  |  | drugOrder | 0.052 | 0.052 | 0.996 | 0.324 |
|  |  | fiveDASC | 0.017 | 0.022 | 0.786 | 0.436 |
| **FailureFB > NeutralFB** | **Nucleus accumbens** | measurement | 0.072 | 0.066 | 1.102 | 0.276 |
|  |  | drug | 0.019 | 0.294 | 0.065 | 0.948 |
|  |  | ketLvl | 0.000 | 0.000 | 0.109 | 0.914 |
|  |  | sex | 0.031 | 0.069 | 0.443 | 0.660 |
|  |  | age | 0.089 | 0.045 | 1.961 | 0.056 |
|  |  | drugOrder | 0.004 | 0.065 | 0.056 | 0.956 |
|  |  | fiveDASC | -0.046 | 0.034 | -1.386 | 0.173 |
| **FailureFB > NeutralFB** | **Insula** | measurement | 0.160 | 0.067 | 2.373 | 0.022* |
|  |  | drug | -0.003 | 0.348 | -0.009 | 0.993 |
|  |  | ketLvl | 0.000 | 0.000 | -0.122 | 0.903 |
|  |  | sex | 0.017 | 0.120 | 0.141 | 0.888 |
|  |  | age | 0.116 | 0.080 | 1.444 | 0.156 |
|  |  | drugOrder | 0.150 | 0.115 | 1.302 | 0.199 |
|  |  | fiveDASC | 0.054 | 0.043 | 1.280 | 0.207 |
| **FailureFB > NeutralFB** | **Thalamus** | measurement | 0.059 | 0.035 | 1.699 | 0.096 |
|  |  | drug | -0.235 | 0.156 | -1.501 | 0.140 |
|  |  | ketLvl | 0.000 | 0.000 | 1.282 | 0.206 |
|  |  | sex | -0.023 | 0.037 | -0.631 | 0.531 |
|  |  | age | 0.027 | 0.024 | 1.125 | 0.266 |
|  |  | drugOrder | 0.048 | 0.034 | 1.393 | 0.170 |
|  |  | fiveDASC | 0.000 | 0.018 | 0.018 | 0.985 |
| **FailureFB > NeutralFB** | **Amygdala** | measurement | 0.008 | 0.063 | 0.134 | 0.894 |
|  |  | drug | -0.163 | 0.306 | -0.533 | 0.596 |
|  |  | ketLvl | 0.000 | 0.000 | 0.371 | 0.713 |
|  |  | sex | -0.132 | 0.084 | -1.578 | 0.121 |
|  |  | age | 0.100 | 0.055 | 1.805 | 0.078 |
|  |  | drugOrder | 0.122 | 0.079 | 1.537 | 0.131 |
|  |  | fiveDASC | 0.030 | 0.036 | 0.823 | 0.415 |

**Table S7. Scan-level median values of the SLOMOCO-derived approximate FD-equivalent motion metric by measurement session and treatment condition.**

| **Participant** | **M1 (mm)** | **M2 (mm)** | **Ketamine session** | **Ketamine (mm)** | **Placebo (mm)** |
| --- | --- | --- | --- | --- | --- |
| **1** | 0.1260 | 0.1485 | 1 | 0.1260 | 0.1485 |
| **2** | 0.1050 | 0.0750 | 1 | 0.1050 | 0.0750 |
| **3** | 0.0790 | 0.0690 | 1 | 0.0790 | 0.0690 |
| **4** | 0.1490 | 0.1365 | 2 | 0.1365 | 0.1490 |
| **5** | 0.1030 | 0.0995 | 2 | 0.0995 | 0.1030 |
| **6** | 0.1210 | 0.1020 | 1 | 0.1210 | 0.1020 |
| **7** | 0.1060 | 0.0920 | 2 | 0.0920 | 0.1060 |
| **8** | 0.2575 | 0.1545 | 2 | 0.1545 | 0.2575 |
| **9** | 0.0750 | 0.0770 | 2 | 0.0770 | 0.0750 |
| **10** | 0.1505 | 0.1700 | 1 | 0.1505 | 0.1700 |
| **11** | 0.2150 | 0.1730 | 1 | 0.2150 | 0.1730 |
| **12** | 0.0900 | 0.0885 | 2 | 0.0885 | 0.0900 |
| **13** | 0.1200 | 0.1690 | 1 | 0.1200 | 0.1690 |
| **14** | 0.1360 | 0.1445 | 1 | 0.1360 | 0.1445 |
| **15** | 0.1040 | 0.1085 | 1 | 0.1040 | 0.1085 |
| **16** | 0.1050 | 0.1200 | 1 | 0.1050 | 0.1200 |
| **17** | 0.0995 | 0.0980 | 2 | 0.0980 | 0.0995 |
| **18** | 0.0990 | 0.1115 | 2 | 0.1115 | 0.0990 |
| **19** | 0.0860 | 0.1190 | 2 | 0.1190 | 0.0860 |
| **20** | 0.0620 | 0.0640 | 1 | 0.0620 | 0.0640 |
| **21** | 0.1940 | 0.2050 | 1 | 0.1940 | 0.2050 |
| **22** | 0.0590 | 0.0780 | 2 | 0.0780 | 0.0590 |
| **23** | 0.1450 | 0.1360 | 1 | 0.1450 | 0.1360 |
| **24** | 0.1340 | 0.1190 | 2 | 0.1190 | 0.1340 |
| **25** | 0.0940 | 0.1370 | 2 | 0.1370 | 0.0940 |
| **26** | 0.1240 | 0.1250 | 1 | 0.1240 | 0.1250 |
| **27** | 0.0910 | 0.0865 | 1 | 0.0910 | 0.0865 |
| **28** | 0.1220 | 0.2050 | 2 | 0.2050 | 0.1220 |

**Supplementary Figures:**

**Figure S1. Monetary Incentive Delay (MID) task visualization** Visualization of the MID task phases (Cue, Target, Feedback and Interstimulus interval) from the subject’s perspective in the scanner.

A

B

**Figure S2.** **Plasma concentration–time curves of ketamine (A, red) and norketamine (B, blue) following a 40-min intravenous infusion of racemic ketamine (0.5 mg/kg).** Each line represents an individual subject (n = 28). Ketamine concentrations peak at infusion stop, while norketamine shows a delayed peak due to metabolic conversion.

**Figure S3.** **Individual 5D-ASC dimension scores for placebo (left) and ketamine (right) conditions (n = 27).** Line plots depict individual summed scores for the five canonical dimensions of the 5-Dimensional Altered States of Consciousness Rating Scale (5D-ASC): Oceanic Boundlessness (OBN), Anxious Ego Dissolution (AED), Visionary Restructuralization (VIS), Auditory Alterations (AUD), and Reduction of Vigilance (VIG), as well as total 5D-ASC scores (“Sum”). Same colours are used for the same subjects across the dimensions. Few individual dimension scores, e.g., OBN and VIG, of two subjects were higher in the placebo condition, which was their first measurement. One of these subjects suspected to have initially received ketamine, the second participant was unable to decide. The second treatment session, where they received ketamine, was later correctly identified by both. The 5D-ASC sum scores were higher for verum than placebo in both individuals.

**Figure S4.** **Mean reaction times during the MID task for placebo and ketamine conditions.** Reaction times did not significantly differ between ketamine and placebo sessions, irrespective of session order.

**Figure S5. Group-level whole-brain activation maps for the expectation–outcome congruency contrast are shown separately for the placebo (top) and ketamine (bottom) conditions.** Statistical maps are thresholded at p < 0.001 (uncorrected) for visualization. Activation was primarily observed in occipital areas, with additional clusters in medial frontal regions.

**Figure S6. Distribution of head motion by treatment condition and measurement session.** Violin plots show the distributions of scan-level median values of the approximate framewise displacement (FD)-equivalent metric retrospectively derived from SLOMOCO motion estimates. Distributions are presented separately for placebo and ketamine scans (left) and for the first and second measurement sessions (M1 and M2; right).
